# Direct, Late‐Stage Mono‐*N*‐arylation of Pentamidine: Method Development, Mechanistic Insight, and Expedient Access to Novel Antiparastitics against Diamidine‐Resistant Parasites

**DOI:** 10.1002/cmdc.202100509

**Published:** 2021-09-02

**Authors:** Jack Robertson, Marzuq A. Ungogo, Mustafa M. Aldfer, Leandro Lemgruber, Fergus S. McWhinnie, Bela E. Bode, Katherine L. Jones, Allan J. B. Watson, Harry P. de Koning, Glenn A. Burley

**Affiliations:** ^1^ Department of Pure and Applied Chemistry University of Strathclyde 295 Cathedral Street Glasgow G1 1XL UK; ^2^ Institute of Infection, Immunity, and Inflammation College of Medical, Veterinary, and Life Sciences University of Glasgow Glasgow G12 8TA UK; ^3^ Glasgow Imaging Facility Institute of Infection, Immunity, and Inflammation College of Medical Veterinary and Life Sciences University of Glasgow Glasgow G12 8TA UK; ^4^ EaStCHEM School of Chemistry University of St Andrews North Haugh St Andrews Fife KY16 9ST UK; ^5^ GlaxoSmithKline Medicines Research Centre Gunnels Wood Road Stevenage Hertfordshire SG1 2NY UK

**Keywords:** antiparasitics, amidine, arylation, copper, medicinal chemistry

## Abstract

A selective mono‐*N*‐arylation strategy of amidines under Chan‐Lam conditions is described. During the reaction optimization phase, the isolation of a mononuclear Cu(II) complex provided unique mechanistic insight into the operation of Chan‐Lam mono‐*N*‐arylation. The scope of the process is demonstrated, and then applied to access the first mono‐*N*‐arylated analogues of pentamidine. Sub‐micromolar activity against kinetoplastid parasites was observed for several analogues with no cross‐resistance in pentamidine and diminazene‐resistant trypanosome strains and against *Leishmania mexicana*. A fluorescent mono‐*N*‐arylated pentamidine analogue revealed rapid cellular uptake, accumulating in parasite nuclei and the kinetoplasts. The DNA binding capability of the mono‐*N*‐arylated pentamidine series was confirmed by UV‐melt measurements using AT‐rich DNA. This work highlights the potential to use Chan‐Lam mono‐*N*‐arylation to develop therapeutic leads against diamidine‐resistant trypanosomiasis and leishmaniasis.

## Graphical abstract

**Accessing antiparasitic amidines**: A selective mono‐*N*‐arylation strategy of amidines under Chan‐Lam conditions was used to access the first mono‐*N*‐arylated analogues of pentamidine. Sub‐micromolar activity against kinetoplastid parasites was observed for several analogues with no cross‐resistance in pentamidine and diminazene‐resistant trypanosome strains and against *Leishmania mexicana*. This work highlights the potential of Chan‐Lam mono‐*N*‐arylation to develop therapeutic leads against diamidine‐resistant trypanosomiasis and leishmaniasis.

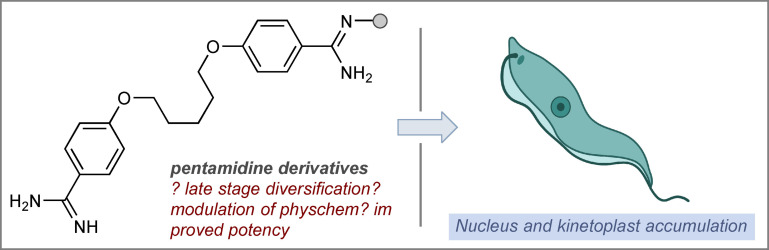

Amidines are essential functional groups used throughout medicinal chemistry.^[1]^ The versatility of this motif arises from their ability to form strong, bifurcated hydrogen bonds and electrostatic interactions with a range of hydrogen bond acceptors and conjugate bases.^[2]^ In addition, modulating the basicity of the amidine group (p*K*_*a*H_∼13–14) alters the overall physicochemical properties, potency, and selectivity of amidine‐containing therapeutics.^[3]^ A prominent exemplar of this approach is the development of the DNA‐binding antiparasitic pentamidine for the treatment of early‐stage *Trypanosoma brucei gambiense*‐related human African trypanosomiasis (HAT) or sleeping sickness,^[4]^ AIDS‐related pneumocystis pneumonia, and leishmaniasis (Scheme 1a).^[5]^ The broader importance of diamidine antiparasitics is further exemplified by their use as the main treatment for animal African trypanosomiasis (AAT), caused by the related parasite *Trypanosoma congolense*, which has a billion‐dollar adverse impact on emerging economies.^[6]^

**Scheme 1:**
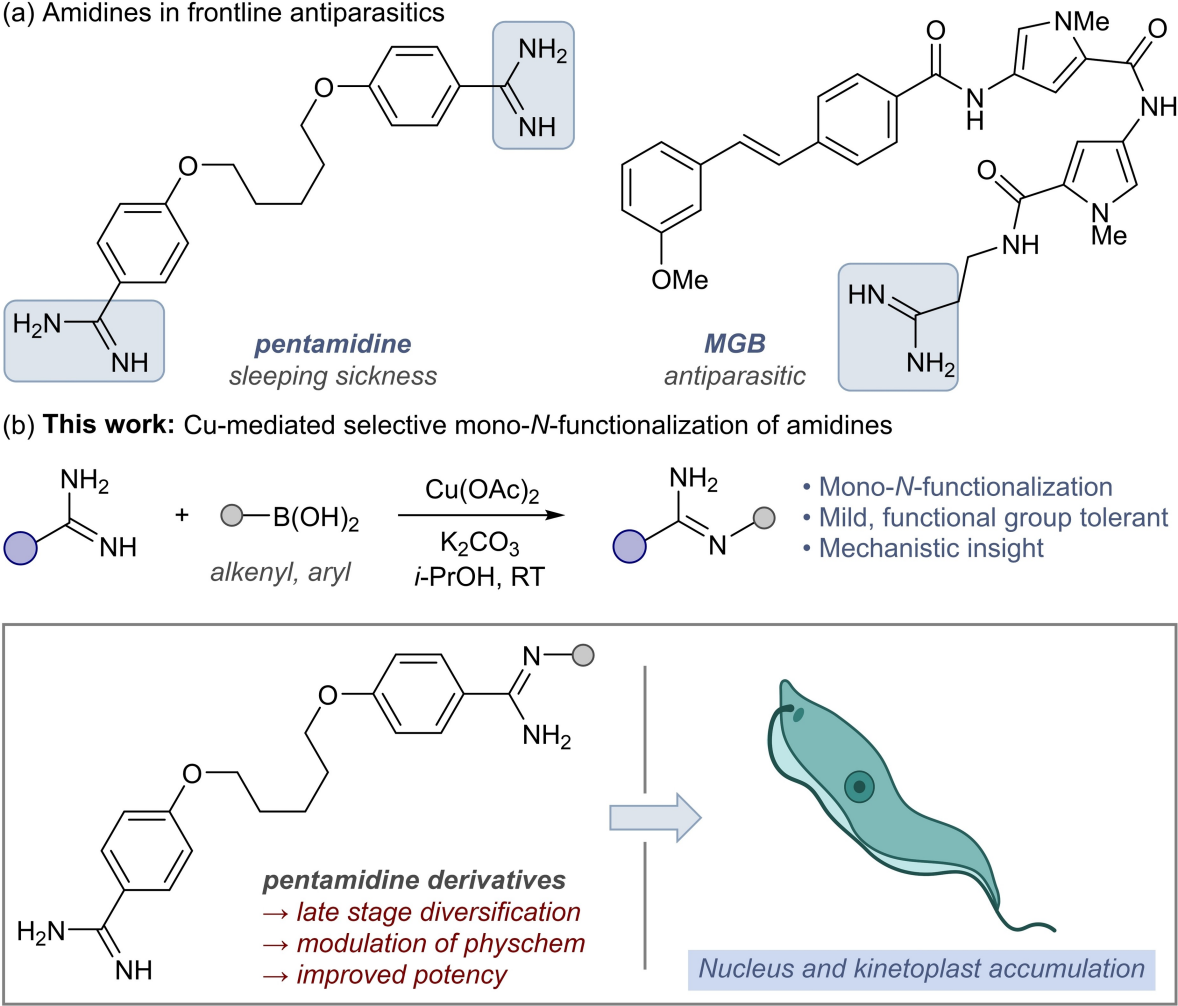
(a) Amidines in antiparasitic agents. (b) Selective amidine mono‐*N*‐arylation and application to development of pentamidine antiparasitics. MGB=minor groove binder.

Poor oral bioavailability and the emergence of pentamidine‐resistant strains has spurred the exploration of new DNA‐binding analogues (Scheme 1a).^[7]^ Modification of the essential amidine moiety is a nascent strategy to by‐pass resistance mechanisms in these parasites by modulating the affinity of diamidines for key drug transporters in *T. brucei* ^[8]^ [*e. g*., TbAT1/P2 aminopurine transporters,^[9]^ High Affinity Pentamidine Transporter (HAPT)^[10]^].^[11]^ However, expedient synthetic access to mono‐*N*‐arylated amidines has been hampered by the lack of robust synthetic methodology suitable for structure‐activity relationship profiling:^[12]^ existing Pinner‐style methodology^[13]^ is unsuited to this challenge due to forcing reaction conditions limiting functional group tolerance, and the lack of chemoselective control leading to competing di/tri‐*N*‐arylation.^[14]^ By virtue of readily‐available starting materials, Pd‐^[15]^ and Cu‐catalyzed cross couplings (*e. g*., Ullmann,[^3c^, ^16^] Chan‐Lam^[17]^) provide the opportunity to selectively access mono‐*N*‐functionalised amidine analogues.

Here we report a mild and chemoselective approach to exemplify amidine mono‐*N*‐arylation *via* the optimization of a Chan‐Lam strategy (Scheme 1b). We expand the substrate scope and demonstrate its application to access, for the first time, mono‐*N*‐arylated pentamidines as anti‐parasitic leads for treatment of HAT, AAT, and *leishmaniasis*.^[18]^

A prominent challenge of amidine functionalization is controlling mono‐ vs. di/tri‐*N*‐arylation.[^17b^, ^19^] Although selective amidine *N*‐arylation strategies have been reported, these are embedded within cascade processes alongside C−H activation events.[^17b^, ^20^] As such, the identification of reaction variables which influence mono‐ vs. di/tri‐*N*‐arylation was first established.

Under standard Chan‐Lam conditions,^[21]^ a model system using benzamidine (**1**) and PhB(OH)_2_ gave 73 % overall yield and *ca*. 4 : 1 selectivity in favor of the mono‐ (**2**) vs. *N*,*N’*‐diarylated product **3** (Entry 1, Table 1). Using an excess of PhB(OH)_2_ led to 82 % total yield with *ca*. 9 : 1 selectivity for **3** (Entry 2), which suggested selective mono‐*N*‐arylation vs. *N*,*N*’‐diarylation is possible by controlling reagent stoichiometry. No tri‐*N*‐functionalization products were observed. Vantourout's B(OH)_3_‐based conditions were less effective (Entry 3).^[22]^ Li's NaOPiv‐based conditions provided a high conversion and *ca*. 40 : 1 selectivity for **2** (Entry 4);^[17b]^ however, further exploration of these conditions did not improve the conversion to **2** (Table S5). The observation of a pronounced base effect led to a focused screen and identification of K_2_CO_3_ as the optimal additive (Entries 5–9). This screen ultimately provided selective mono‐*N*‐arylation set of conditions using 20 mol% Cu(OAc)_2_ giving **2** in 81 % isolated yield and without any observable generation of **3** (Entry 10).

**Table 1:** Reaction development.^[a]^

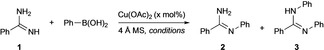		
Entry	Conditions	**2**/**3** (%)^[b]^
1^[c]^	Cu(OAc)_2_ (100 mol%), Et_3_N, CH_2_Cl_2_, RT, 16 h	59/14
2^[c,d]^	Cu(OAc)_2_ (100 mol%), Et_3_N, CH_2_Cl_2_, RT, 16 h	8/74
3	Cu(OAc)_2_ (100 mol%), B(OH)_3_, CH_2_Cl_2_, RT, 16 h	45/11
4^[d]^	Cu(OAc)_2_ (100 mol%), NaOPiv, DMF, 50 °C, 16 h	81/2
5	Cu(OAc)_2_ (100 mol%), K_2_CO_3_, CH_2_Cl_2_, RT, 16 h	40/8
6	Cu(OAc)_2_ (100 mol%), K_2_CO_3_, MeOH, RT, 16 h	59/2
7^[c]^	Cu(OAc)_2_ (100 mol%), K_2_CO_3_, *i*‐PrOH, RT, 2 h	83/5
8	Cu(OAc)_2_ (50 mol%), K_2_CO_3_, *i*‐PrOH, 50 °C, 24 h	76/2
**9** ^[c]^	**Cu(OAc)_2_ (20 mol%), K_2_CO_3_**, ***i*** **‐PrOH, 50 °C, 24 h**	**81/0**

[a] Using 2 : 1 **1** : PhB(OH)_2_ and 2 equiv. of additive unless noted. [b] Determined by HPLC analysis using standard concentration curves of **2** and **3**. [c] Isolated yield on 1 mmol scale. [d] **1** : PhB(OH)_2_=1 : 2. [e] **1** : PhB(OH)_2_=1 : 1.2.

In contrast to our experience with other Chan‐Lam processes,[^22^, ^23^] reactions using **1** and K_2_CO_3_ produced a purple precipitate within the first 5 minutes of the reaction (Figure S3). X‐ray crystallographic analysis revealed a *C2*‐symmetric mononuclear Cu(II) complex **4** (Scheme 2a) consisting of a bidentate carbonate ligand and two monodentate benzamidines. This unique complex suggests that K_2_CO_3_ plays a dual role in this system, acting both as a ligand and base. A dual ligand/base role has been speculated in some Cu(OAc)_2_‐based Chan‐Lam reactions;^[24]^ however, structural information has been limited to the observation of a single tetranuclear Cu(II) complex.^[22]^

**Scheme 2:**
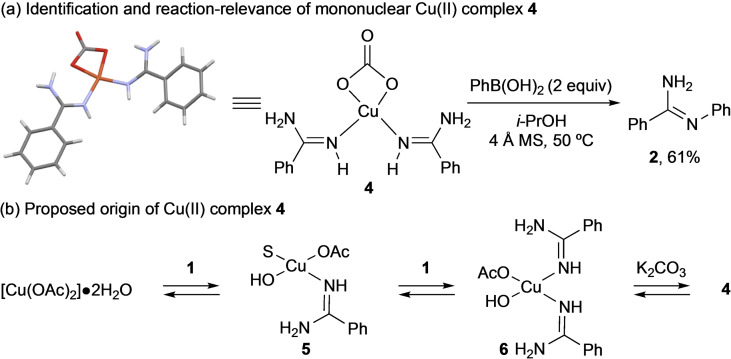
(a) Structure of Cu(II) complex **4**. (b) Chan‐Lam bond formation using complex 4. (c) Proposed mechanistic origin of complex **4**. S=solvent.

Exposing **4** to PhB(OH)_2_ in *i*‐PrOH afforded **2** in 61 % yield, suggesting **4** is a reaction‐relevant intermediate (Scheme 2a). The role of **4** was further investigated by EPR analysis of reaction mixtures. No EPR signals were observed when [Cu(OAc)_2_] ⋅ 2H_2_O was treated with K_2_CO_3_ or PhB(OH)_2_. In contrast, a paramagnetic species was observed upon addition of **1** (Figure S2). This suggests that **1** is responsible for paddlewheel denucleation, consistent with mechanistic proposals for the Chan‐Lam amination.[^22^, ^25^] Complex **4** could form after paddlewheel denucleation *via* ligand exchange processes at, for example, putative Cu(II) complexes **5** and **6** (Scheme 2b).

Supplementary to our main aim of using a Chan‐Lam strategy for the late‐stage mono‐*N*‐arylation of pentamidine, the generality of the approach was assessed using a range of amidines and arylboronic acids, all of which exclusively formed mono‐*N*‐arylated products (**2**–**29**, Scheme 3a, Table S6). The use of 20 mol% Cu(OAc)_2_ was comparable to stoichiometric Cu(OAc)_2_; however, for expediency, the latter was used across the series based on the faster reaction time (see Table 1, Entries 7 and 9) with comparable yields afforded for both processes (see yields for catalytic reactions in parentheses).

**Scheme 3:**
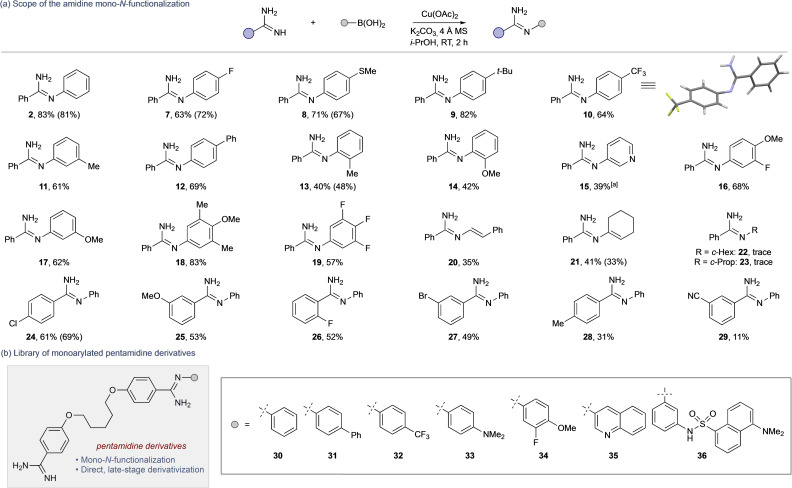
(a) Scope of mono‐*N*‐arylation. Yields in parentheses for reactions at 50 °C for 24 h using 20 mol% Cu(OAc)_2_. (b) Library of monoarylated pentamidine derivatives. [a] Isolated as the corresponding TFA salt.

Both mono‐ and bisamidines are known Cu‐chelators,^[26]^ which could hamper the wider utility of this approach to mono‐*N*‐arylate pentamidine for structure‐activity‐relationship (SAR) profiling. Indeed, standard Chan‐Lam conditions failed to deliver any *N*‐arylation of pentamidine. Gratifyingly, our reaction conditions were leveraged to directly access a series of exclusively mono‐*N*‐arylated pentamidines (**30**–**36**, Scheme 3b) in a single step from pentamidine.[^5b^, ^27^]

This focused series covered analogues ranging from unsubstituted (**30**), mono‐substituted (**31**–**33**) and disubstituted aromatics (**34**), a quinolone (**35**), and the bulky fluorescent analogue (**36**) suitable for cellular uptake studies.

Compounds (**30**–**36**) were then tested for activity against three kinetoplastid parasite species that are commonly treated with pentamidine (*T. brucei*, *L. mexicana*) or diminazene aceturate (*T. congolense*) (Table 2).^[28]^ EC_50_ values ranged from 0.19 μM to 1.1 μM, with **33** exhibiting the greatest potency followed by **34**. Indeed, for *T. brucei*, the SAR was quite flat, with quinoline (**35**) and biphenyl (**31**) analogues displaying similar EC_50_s to the phenyl analogue (**30**).

**Table 2:** Activity of mono‐*N*‐aryl pentamidine analogues **30**–**36** against kinetoplastid parasites.

Entry	Compound	*T. b. brucei*				*T. congolense*				*L. mexicana*
		EC_50_ [μM; n=3]		RF	t‐test	EC_50_ [μM; n=3]		RF	t‐test	EC_50_ [μM; n=4]
		WT	B48			WT	DA‐Res			
1	**30**	1.10±0.07	1.31±0.02	1.19	0.042	2.94±0.10	3.26±0.01	1.11	0.037	12.7±0.7
2	**31**	0.91±0.02	0.86±0.02	0.94	0.10	n.d.	n.d.	–	–	2.12±0.07
3	**32**	0.65±0.03	0.37±0.006	0.57	0.0007	1.26±0.03	1.56±0.06	1.23	0.012	9.71±0.56
4	**33**	**0.19±0.003**	**0.28±0.006**	**1.47**	**0.0002**	**0.64±0.02**	**0.75±0.003**	**1.23**	**0.0008**	**0.66±0.04**
5	**34**	0.51±0.004	0.51±0.02	1.01	0.82	n.d.	n.d.	–	–	>20
6	**35**	1.09±0.01	0.82±0.01	0.75	0.0001	n.d.	n.d.	–	–	>20
7	**36**	3.04±0.02	2.98±0.04	0.98	0.23	6.69±0.11	9.29±0.17	1.39	0.0002	7.54±0.59
8	**PMD**	0.0024±0.0002	0.28±0.002	116	3.5×10^−8^	1.31±0.02	1.42±0.01	1.08	0.013	2.04±0.04
9	**DA**	0.082±0.006	0.89±0.05	10.8	0.0001	0.27±0.008	1.71±0.02	6.4	2×10^−7^	n.d.

WT=wild‐type, PMD=pentamidine, DA=diminazene aceturate, DA‐Res=diminazene‐resistant cell line, RF=resistance factor being the ratio of EC_50_ (resistant line) over EC_50_(WT). Statistical difference between WT and resistant pairs of cell lines was established using a Student unpaired two‐tailed t‐test.

The compounds were also tested against the related kinetoplastid parasites *L. mexicana* and *T. congolense*. Here, **33** displayed >2‐fold and >3‐fold higher activity compared to pentamidine against *T. congolense* and *L. mexicana*, respectively. The SAR profile was considerably less flat than for *T. brucei*, with **33** being 4.6‐fold and 19.2‐fold more active than **30** against *T. congolense* and *L. mexicana*, respectively.

The potential for cross‐resistance with diamidines and melaminophenyl arsenicals (MPAs; including melarsoprol, cymelarsan) is particularly problematic in the development of next‐generation amidine antiparasitics.^[4]^ Resistance likely emerges from active drug transport [*e. g*., TbAT1/P2 aminopurine transporter;^[29]^ High Affinity Pentamidine Transporter (TbAQP2)] into the parasite's interior.^[30]^ Having studied the SAR of the transporter‐diamidine interactions, we hypothesized that mono‐*N*‐arylation of the pentamidine scaffold would abolish their ability to act as substrates for the *T. brucei* drug transporters, and therefore would not display cross‐resistance with pentamidine and MPAs.^[31]^ To test this hypothesis, **30**–**36** were also tested against a *T. b. brucei* clonal line, B48,^[32]^ lacking both the TbAT1/P2 and HAPT1/AQP2 transporters, and against a diminazene‐resistant *T. congolense* ^[11c]^ clone. Although minor differences in drug sensitivity were observed (±50 % of WT EC_50_), these variations were trivial compared to the resistance levels to pentamidine (116‐fold) and diminazene (6.4‐fold), respectively. We therefore conclude that mono‐*N*‐arylated pentamidine analogues are not cross‐resistant with un‐substituted diamidines, arising from differences in their mechanism of uptake.

To gain further insights into the uptake of the mono‐*N*‐arylated pentamidines, the fluorescent analogue (**36**) was used to monitor uptake in each of the three parasite species in real‐time (Figure 1). In all three species, **36** was taken up rapidly (3.3 μM and 10 μM), consistent with an EC_50_≈3–7 μM against the kinetoplastid species (Table 2). The rate of uptake of **36** was dose‐dependent and showed an approximately 2.3–10‐fold higher rate at 10 μM [**36**] than for 3.3 μM after 30 min (Figure 1). This shows that the uptake mechanism was not saturable in the lower μM range, unlike TbAT1/P2 and HAPT/TbAQP2, which display *K_m_* values of 0.26±0.03 and 0.036±0.06 μM, respectively, for pentamidine.^[30]^ Moreover, the rate of uptake of **36** was not substantially different in the presence of 10 μM pentamidine (within 10 % in all cases), clearly indicating that uptake did not involve a high affinity pentamidine transporter. Taken collectively, these data show that mono‐*N*‐arylated analogues such as **36** evade known pentamidine transporters TbAT1 and TbAQP2 in *T. brucei*.

**Figure 1:**
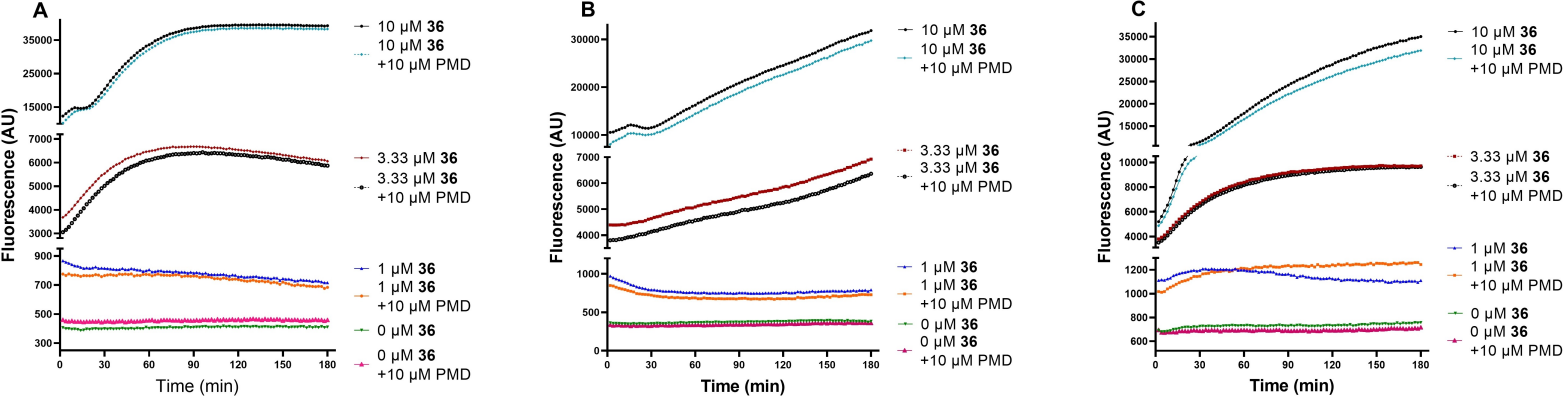
Real time fluorescence development of **36** with bloodstream forms of WT *T. b. brucei* (A), bloodstream forms of WT *T. congolense*, (B) and promastigotes of *L. mexicana* (C). Cells were incubated for 3 h in the presence of **36** at 0, 1, 3.3 or 10 μM, in the presence or absence of 10 μM pentamidine. Measurements were taken at 2‐minute intervals. A.U., artificial units of fluorescence intensity.

One of the putative mechanisms of action of pentamidine is the inhibition of replication *via* binding to A/T‐rich sequences of duplex DNA.^[33]^ Indeed, selective accumulation of **36** to the nucleus and kinetoplast of *T. brucei* was observed, as confirmed by co‐localization with Hoechst 33342 (Figure 2), consistent with DNA binding. UV melt analyses were undertaken to explore the ability of **33** and **36** to stabilize DNA duplexes relative to pentamidine (Table S7, Figure S4). A 7.0 °C stabilization of an A‐tract duplex was observed for **33**, relative to a 6.0 °C stabilization for pentamidine. Although a reduced level of duplex stabilization was observed using **36** for the same sequence (ΔT_m_ 4.6 °C), both analogues exhibited a similar binding bias for an A‐tract duplex relative to a duplex containing an alternating A‐T sequence.^[34]^ These data indicate that the likely mechanism of action of these analogues is *via* binding to DNA duplexes with a selectivity profile similar to that observed for pentamidine. The stronger DNA binding of **33** correlated with its stronger anti‐kinetoplastid activity compared to **36**.

**Figure 2:**
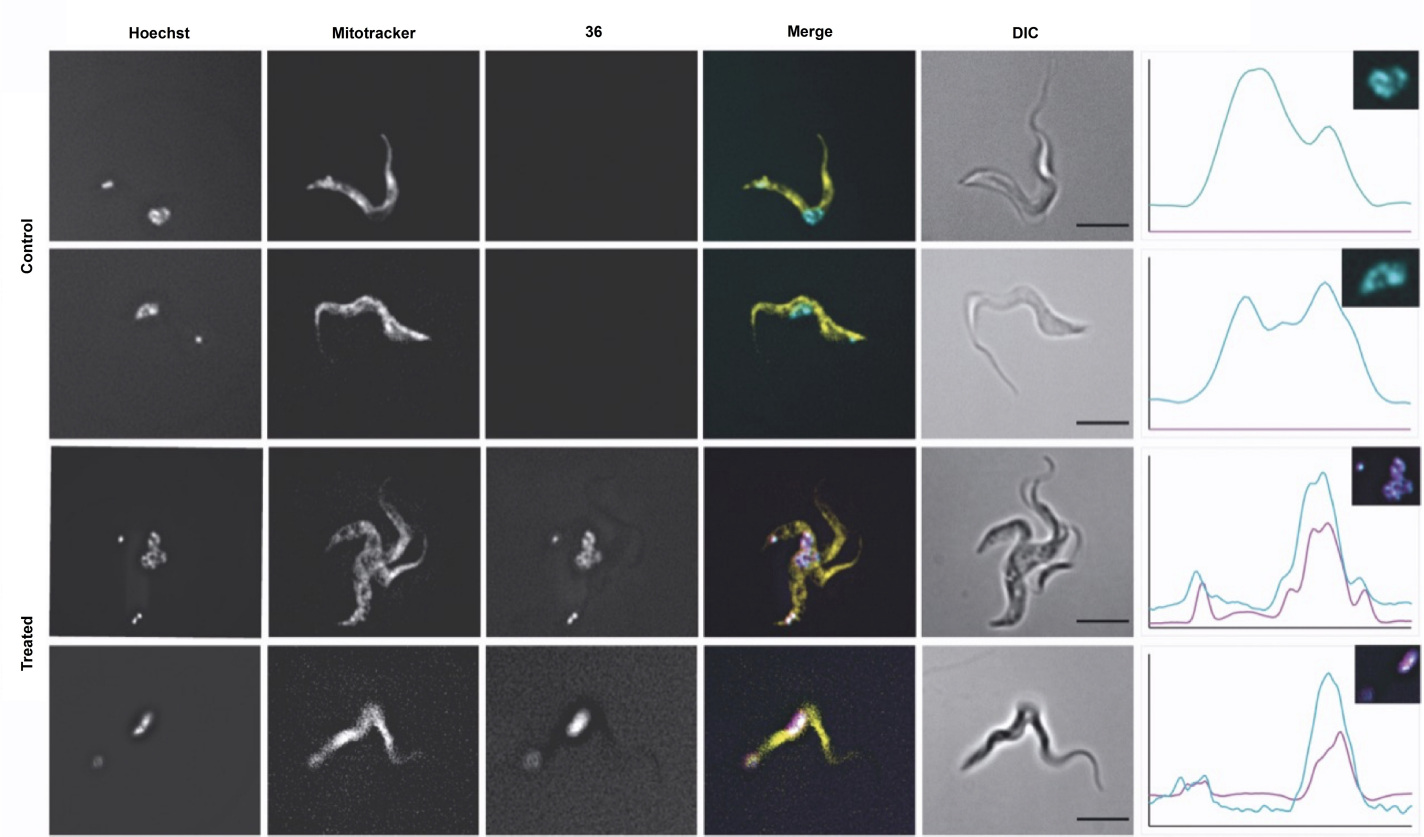
Selected immunofluorescence images of *T. brucei* labelled with a nuclear marker (Hoechst 33342), a dye for the mitochondrion (MitoTracker Green), and with **36** (30 min exposure, [10 μM]) or not (control). Charts show the intensity levels of the DAPI and red filter channels measured in the nuclei and kinetoplast. Scale bars are 5 μm.

In summary, we have developed a general approach to prepare mono‐*N*‐arylated amidines from mono‐ and bisamidine substrates based on Chan‐Lam cross‐coupling methodology. During our optimization phase, key mechanistic insights into Cu intermediates pertinent to the Chan‐Lam reaction were identified, and the procedure was broadly applicable to the formation of mono‐*N*‐arylated substrates. This methodology was used to directly prepare mono‐*N*‐arylated analogues of pentamidine, which displayed promising *in vitro* activity against three species of kinetoplast parasites of clinical and veterinary importance. Most importantly, the mono‐*N*‐arylated analogues were not cross‐resistant with pentamidine and diminazene, bypassing known drug transporters. In addition, **36** accumulated rapidly in all three kinetoplastid species and localized to the parasite nucleus and kinetoplast, consistent with a putative mechanism of action being a DNA minor groove binder. These findings highlight that the potential utility of mono‐*N*‐arylation of diamidines, and potentially their expansion to guanidine analogues,^[35]^ as an emerging class of therapeutic agents against neglected parasitic diseases.

## Conflict of interest

The authors declare no conflict of interest.

## Supporting information

As a service to our authors and readers, this journal provides supporting information supplied by the authors. Such materials are peer reviewed and may be re‐organized for online delivery, but are not copy‐edited or typeset. Technical support issues arising from supporting information (other than missing files) should be addressed to the authors.

Supporting Information
