# Supplementary material for: Direct, Late‐Stage Mono‐N‐arylation of Pentamidine: Method Development, Mechanistic Insight, and Expedient Access to Novel Antiparastitics against Diamidine‐Resistant Parasites
Source: ChemMedChem. 2021 Sep 2;16(22):3396–401. doi: 10.1002/cmdc.202100509 (PMC9291547; doi:10.1002/cmdc.202100509)
Supplement: Supplementary file 1 — Supporting Information [file CMDC-16-3396-s001.pdf]

ChemMedChem

Supporting Information

Direct, Late-Stage Mono-*N*-arylation of Pentamidine: Method Development, Mechanistic Insight, and Expedient Access to Novel Antiparastitics against Diamidine-Resistant Parasites

Jack Robertson, Marzuq A. Ungogo, Mustafa M. Aldfer, Leandro Lemgruber, Fergus S. McWhinnie, Bela E. Bode, Katherine L. Jones,* Allan J. B. Watson,* Harry P. de Koning,* and Glenn A. Burley*

Contents

General information 3

Analytical HPLC 3

Purification of products 3

Analysis of products 4

Sample preparation for EPR spectroscopy 4

EPR measurements 5

X-Ray crystalography 5

Synthetic procedures 5

 Chan-Lam procedure A 5

 Chan-Lam procedure B 6

 Chan-Lam procedure C 6

 Chan-Lam procedure D 6

 Reaction optimisation 6

 Cu source/benzamidine eq trials 9

Representative HPLC trace 10

EPR results 10

Precipitate formation in reactions 12

Compound characterisations 12

Trypanosoma experiments 27

 Resazurin-based drug sensitivity assay 27

 Real-time measurement of fluorescence in parasites 28

 Localisation of cellular targets using fluorescence microscopy 28

 DNA UV Melt Analysis 29

NMR spectra 31

Crystallographic data tables 68

References 87

General information

All reagents and solvents were obtained from commercial suppliers and were used without further purification. Pentamidine free base was synthesised from pentamidine isethionate by previously reported methods.[1] All glassware was oven-dried (150 °C) before use. All reactions were carried out under air. Reactions were monitored by thin layer chromatography (TLC) using Merck silica plates coated with fluorescent indicator UV254. TLC plates were analysed using 254/365 nm UV light or developed using potassium permanganate solution.

Analytical HPLC

RP-HPLC was carried out using an Aeris 3.6 µm RP 250 x 4.6 mm widepore XB C18 column using a DIONEX 3000 series HPLC system equipped with a VWD3400 photodiode array detector. Analysis was performed using the method outlined below.

The absorbance was detected at 254 nm. Solvent A: Water (0.1 % TFA) Solvent B: Acetonitrile (0.1 % TFA). Flow rate 1.0 mL/min

Table S1. Analytical HPLC method A.

Time (min)	Solvent A	Solvent B
0	99 %	1 %
5	99 %	1%
30	50 %	50 %
35	50 %	50 %
40	99 %	1 %
45	99 %	1 %

Purification of products

Normal-phase flash chromatography was carried out using ZEOprep 60 HYD 40-63 µm silica gel. Semi-preparative reversed-phase HPLC purification was carried out using a Phenomenex Clarity 5 µm RP 250 x 10.00 mm XB C18 column using a DIONEX 3000 series HPLC system equipped with a VWD3400 variable wavelength detector. Purifications were performed using 2 different systems using water (0.1% TFA) as Solvent A and acetonitrile (0.1% TFA) as Solvent B. The flow rate was set to 12.0 mL/min. RP-HPLC method **A**: The absorbance was detected at 254 nm. Solvent A: Water. Solvent B: Acetonitrile.

Table S2. RP-HPLC method A.

Time (min)	Solvent A	Solvent B
0	99 %	1 %
5	99 %	1%
45	50 %	50 %
50	50 %	50 %
55	99 %	1 %
60	99 %	1 %

RP-HPLC method **B**: The absorbance was detected at 254 nm. Solvent A: Water (0.1 % TFA). Solvent B: Acetonitrile (0.1 % TFA).

Table S3. RP-HPLC method B**.**

Time (min)	Solvent A	Solvent B
0	99 %	1 %
5	99 %	1%
25	50 %	50 %
30	50 %	50 %
35	10 %	90 %
40	10 %	90 %
45	99 %	1 %
50	99 %	1 %

Analysis of products

Fourier-Transform Infra-Red (FTIR) spectra were obtained on a Shimadzu IRAffinity-1 spectrometer. ^{19}F NMR spectra were obtained on a Bruker AV 400 spectrometer at 376 MHz. ^{1}H and ^{13}C NMR spectra were obtained on either a Bruker AV 400 at 400 MHz and 125 MHz, respectively, or Bruker DRX 500 at 500 MHz and 126 MHz, respectively. Chemical shifts are reported in ppm and coupling constants are reported in Hz. High-resolution mass spectra were recorded on an LTQ Orbitrap XL 1 mass spectrometer at the EPSRC UK National Mass Spectrometry Facility (Swansea) and a Thermo Exactive Orbitrap mass spectrometer at the University of St Andrews.

Sample preparation for EPR spectroscopy

To 10 mL glass vials was added $Cu(OAc)_2$ (36.0 mg, 0.20 mmol) in *i*-PrOH (5.20 mL) to give a solution of 38.5 mM $Cu(OAc)_2$. Appropriate additive quantities were used as detailed in Table S4.

Table S4. Samples prepared for EPR analysis**.**

Sample	Additive(s)	Mass, mmol
1	N/A	N/A
2	Benzamidine (**1**)	48.0 mg, 0.40 mmol
3	K_2CO_3	55.0 mg, 0.40 mmol
4	$PhB(OH)_2$	24.0 mg, 0.20 mmol
5	Benzamidine (**1**), K_2CO_3 and $PhB(OH)_2$	48.0 mg 0.40 mmol, 55.0 mg 0.40 mmol, 24.0 mg 0.20 mmol

EPR measurements

The EPR spectra were obtained using a Bruker EMX 10/12 spectrometer operating at ~9.5 GHz with 100 kHz modulation. Measurements were performed using an ELEXSYS Super High Sensitivity Probehead (Bruker ER4122SHQE). The EPR spectra were recorded at 293 K using 10 mW microwave power, 2000 G field sweep centred at 3300 G with 1024 points resolution. A time constant and conversion time of 40.96 ms was used with a modulation amplitude of 0.8 mT and a microwave frequency of 9.766 GHz. EPR measurements carried out by Dr Bela Bode, University of St. Andrews.

X-Ray crystallography

Data for compounds **4** and **10** were collected on Oxford Diffraction Gemini S or Xcalibur E instruments with graphite-monochromated Mo Kα (λ = 0.71073 Å) or Cu Kα (λ = 1.54184 Å) radiation. Data collection and processing used CrysalisPro software. All structures were solved and refined to convergence on F^2 for all independent reflections by the full-matrix least squares method using SHELXL-20144 or by the Gauss Newton algorithm using OLEX2.5. Crystal structure data for compounds **4** and **10** has been deposited at the Cambridge Crystallographic Data Centre under deposition numbers CCDC2101634 and CCDC 2101824, respectively

Synthetic procedures

Chan-Lam procedure A

To an oven-dried microwave vial was added the corresponding amidine (1.00 mmol), boronic acid (2.00 mmol), $Cu(OAc)_2$ (181 mg, 1.00 mmol), NEt_3 (278 µL, 2.00 mmol) and activated 4Å molecular sieves (1.00 g). Dichloromethane (3.25 mL) was then added to the solid mixture, sealed under air and stirred at room temperature for 16 h. The resulting suspension was filtered and the precipitate was washed with MeOH (3.00 mL). The resulting solution was concentrated *in vacuo* and the residue purified by silica gel chromatography.

Chan-Lam procedure B

To an oven-dried microwave vial was added the corresponding amidine (0.40 mmol), boronic acid (0.20 mmol), $Cu(OAc)_2$ (36.0 mg, 0.20 mmol), K_2CO_3 (55.0 mg, 0.40 mmol) and activated 4Å molecular sieves (200 mg). Isopropanol (0.65 mL) was then added to the solid mixture, sealed under air and stirred at room temperature for 2 h. The resulting suspension was filtered and the precipitate was washed with MeOH (5 × 1 mL). The resulting solution was concentrated *in vacuo* and the residue purified by either silica gel chromatography or RP-HPLC.

Chan-Lam procedure C

To an oven-dried microwave vial was added the corresponding amidine (0.40 mmol), boronic acid (0.20 mmol), $Cu(OAc)_2$ (7.2 mg, 0.04 mmol), K_2CO_3 (55 mg, 0.40 mmol) and activated 4Å molecular sieves (200 mg). Isopropanol (0.65 mL) was then added to the solid mixture, sealed under air and stirred in a sand bath at 50 °C for 24 h. The resulting suspension was filtered and the precipitate was washed with MeOH (5 × 1 mL). The resulting solution was concentrated *in vacuo* and the residue purified by either silica gel chromatography or RP-HPLC.

Chan-Lam procedure D

To an oven dried microwave vial was added pentamidine free base (68.0 mg, 0.20 mmol), boronic acid (0.20 mmol), $Cu(OAc)_2$ (36.0 mg, 0.20 mmol) and activated 4Å molecular sieves (200 mg). MeOH (0.65 mL) was then added to the solid mixture before final addition of Et_3N (56 μL, 0.40 mmol), sealed under air and stirred at room temperature for 16 h. The resulting suspension was filtered, and the precipitate was washed with MeOH (5 × 1.50 mL). The resulting solution was purified by semi-preparative RP-HPLC and lyophilised to afford the amidine product.

Reaction optimisation

To an oven dried 5 mL microwave vial was added benzamidine **1** (0.40 mmol), phenyl boronic acid (0.20 mmol), $Cu(OAc)_2$ (as per Table S5), base or additive (0.40 mmol) and activated 4Å molecular sieves (200 mg). Solvent (0.65 mL) was then added to the solid mixture, sealed under air and stirred at room temperature for a defined time period. A 50 μL aliquot of the reaction was removed and diluted with 950 μL of MeOH. The resulting sample was then analysed by HPLC using analytical HPLC method A. Conversion was calculated using standard concentration curves of *N'*-phenylbenzimidamide (**2**) TFA salt and *N,N'*-diphenylbenzimidamide free base (**3**).

Table S5. Chan-Lam amidination optimisation of conditions.

Entry	Solvent	Reaction time	Additive	$Cu(OAc)_2$ eq	Reaction temp	**2** conv.[a]	**3** conv.[a]

1	DCM	16 h	$B(OH)_3$	1.0	rt	45%	11%
2	DCM	16 h	Et_3N	1.0	rt	58% (59%)[b]	14%
3	MeOH	16 h	K_2CO_3	1.0	rt	59%	2%
4	MeOH	16 h	K_2CO_3	0.5	rt	58%	2%
5	MeOH	16 h	K_2CO_3	1.0	80°C	36%	<1%
6	DMSO	16 h	Et_3N	1.0	rt	47%	2%
7	DMSO	16 h	K_2CO_3	1.0	rt	59%	<1%
8	MeCN	16 h	K_2CO_3	1.0	rt	52%	8%
9	*i*-PrOH	16 h	$B(OH)_3$	1.0	rt	38%	14%
**10**	***i*-PrOH**	**16 h**	**K_2CO_3**	**1.0**	**rt**	**70% (72%)[b]**	**4%**
11	*i*-PrOH	16 h	Et_3N	1.0	rt	70%	6%
12	*i*-PrOH	16 h	K_2CO_3	0.5	rt	7%	0%
13	*i*-PrOH	16 h	K_2CO_3	1.0	80°C	61%	0%
14	*i*-PrOH	16 h	K_2CO_3	1.2	rt	62%	4%
15	*i*-PrOH	16 h	K_2CO_3	1.5	rt	60%	4%
16	*i*-PrOH	16 h	K_2CO_3	2.0	rt	59%	4%
17	*i*-PrOH	16 h	K_2CO_3	1.0	0-10°C	63%	5%
18	*i*-PrOH	16 h	Pyridine	1.0	rt	59%	3%
19	*i*-PrOH	8 h	K_2CO_3	1.0	rt	63%	4%
20	*i*-PrOH	4 h	K_2CO_3	1.0	rt	63%	3%
**21**	***i*-PrOH**	**2 h**	**K_2CO_3**	**1.0**	**rt**	**76% (83%)[b]**	**5%**
22	*i*-PrOH	1 h	K_2CO_3	1.0	rt	58%	3%
23	*i*-PrOH	0.5 h	K_2CO_3	1.0	rt	60%	3%
24	*i*-PrOH	48 h	K_2CO_3	1.0	rt	69%	7%
25	*i*-PrOH	2 h	K_2CO_3	0.5	rt	2%	0%
26	*i*-PrOH	2 h	K_2CO_3 + MnO_2 (3 eq.)	0.5	rt	2%	0%
27	*i*-PrOH	2 h	None	1.0	rt	3%	0%
28	*i*-PrOH:MeOH (3:1)	16 h	K_2CO_3	1.0	rt	63%	<1%

29	*i*-PrOH:MeOH (1:1)	16 h	K_2CO_3	1.0	rt	52%	<1%
30	^{t}BuOH	16 h	K_2CO_3	1.0	rt	49%	10%
31	H_2O	16 h	K_2CO_3	1.0	rt	<1%	0%
32	DMF	2 h	K_2CO_3	1.0	rt	31%	2%
33	DMF	16 h	K_2CO_3	1.0	rt	55%	<1%
34	DCE	2 h	K_2CO_3	1.0	rt	40%	3%
35	MeOH	2 h	Et_3N	1.0	rt	51%	<1%
36	*i*-PrOH	2 h	K_2CO_3	0.5	50°C	56%	--
37	*i*-PrOH	2 h	K_2CO_3	0.2	50°C	62%	--
38	*i*-PrOH	2 h	K_2CO_3	0.1	50°C	18%	--
39	*i*-PrOH	24 h	K_2CO_3	0.5	50°C	76%	2%
**40**	***i*-PrOH**	**24 h**	$\mathbf{K_2CO_3}$	**0.2**	**50°C**	**81%**[b]	**--**
41	*i*-PrOH	24 h	K_2CO_3	0.1	50°C	64%	--
42	DMF	24 h	NaOPiv	0.5	50°C	72%[c,d]	7%[c,d]
43	DMF	24 h	NaOPiv	0.2	50°C	81%[c,d]	2%[c,d]
44	DMF	2 h	NaOPiv	0.2	50°C	54%[c,d]	--
45	*i*-PrOH	2 h	NaOPiv	1.0	rt	66%	4%
46	*i*-PrOH	2 h	NaOPiv	0.5	rt	64%	2%
47	*i*-PrOH	2 h	NaOPiv	0.2	rt	14%	--
48	*i*-PrOH	2 h	NaOPiv	1.0	50°C	71%	5%
49	*i*-PrOH	2 h	NaOPiv	0.5	50°C	68%	2%
50	*i*-PrOH	2 h	NaOPiv	0.2	50°C	47%	--
51	*i*-PrOH	24 h	NaOPiv	1.0	50°C	63%	5%
52	*i*-PrOH	24 h	NaOPiv	0.5	50°C	69%	3%
53	*i*-PrOH	24 h	NaOPiv	0.2	50°C	47%	--
54	DCM	16h	K_2CO_3	1.0	rt	40%	8%
55	MeOH	16h	K_2CO_3	1.0	rt	59%	2%

a) Conversion calculated using standard calibration curve b) Isolated yield on 1.00 mmol scale c) 0.25 M reaction concentration. d) **1**:$PhB(OH)_2$ =1:1.2 used.

Cu source/benzamidine eq trials

To an oven dried 5 mL microwave vial was added benzamidine **1** (as per Table S6), phenyl boronic acid (0.20 mmol), Cu source (0.20 mmol), base or additive (0.40 mmol) and activated 4Å molecular sieves (200 mg). Solvent (0.65 mL) was then added to the solid mixture, sealed under air and stirred at room temperature for desired time. A 50 µL aliquot of the reaction was removed and diluted with 950 µL of MeOH. The resulting sample was then analysed by HPLC using analytical HPLC method A. Conversion was calculated using standard concentration curves of *N'*-phenylbenzimidamide (**2**) TFA salt and *N,N'*-diphenylbenzimidamide (**3**).

Table S6. Reaction optimisation using altering Cu source and eq of benzamidine.

Entry	Solvent	Reaction time	Additive	Cu source	**1** eq.	**2** conv.[a]	**3** conv.[a]
1	MeOH	16 h	K_2CO_3	$Cu(OAc)_2$	1.5	49%	2%
2	MeOH	16 h	K_2CO_3	$Cu(OAc)_2$	1.2	43%	2%
3	DCM	16 h	$B(OH)_3$	$Cu(OAc)_2$	2.0	26%[b]	5%[b]
4	*i*-PrOH	16 h	None	$Cu_2(OH)_2CO_3$	2.0	0%	0%
5	*i*-PrOH	16 h	K_2CO_3	$Cu(OAc)_2$	1.5	63%	5%
6	*i*-PrOH	16 h	K_2CO_3	$Cu_2(OH)_2CO_3$	2.0	<1%	0%
7	*i*-PrOH	16 h	K_2CO_3	$Cu(OAc)_2$	1.0	46%	6%
8	*i*-PrOH	2 h	K_2CO_3	$[Cu(MeCN)]PF_6$ (0.5 eq)	2.0	5%	0%
9	*i*-PrOH	16 h	K_2CO_3	$[Cu(MeCN)]PF_6$	2.0	35%	3%
10	*i*-PrOH	2 h	K_2CO_3	CuOAc	2.0	2%	0%
11	MeOH	2 h	K_2CO_3	CuOAc	2.0	14%	0%
12	*i*-PrOH	2 h	K_2CO_3 + KOAc (1 eq)	CuOAc	2.0	<1%	0%
13	*i*-PrOH	2 h	K_2CO_3	Complex **4**	2.0	2%	0%
14	*i*-PrOH	2 h	K_2CO_3	Complex **4** (0.5 eq), $Cu(OAc)_2$ (0.5 eq)	2.0	2%	0%
15	*i*-PrOH	2 h	K_2CO_3	Complex **4**	2.0	18%[c]	0%
16	*i*-PrOH	24 h	K_2CO_3	Complex **4**	2.0	61%[c]	0%
17	DCM	16 h	Et_3N	$Cu(OAc)_2$	1.0	8%[b,d]	74%[b,d]

a) Conversion calculated using standard calibration curve b) Isolated yield on 1.00 mmol scale c) Reaction heated to 50 °C. d) **1**:$PhB(OH)_2$ =1:2.

Representative HPLC trace

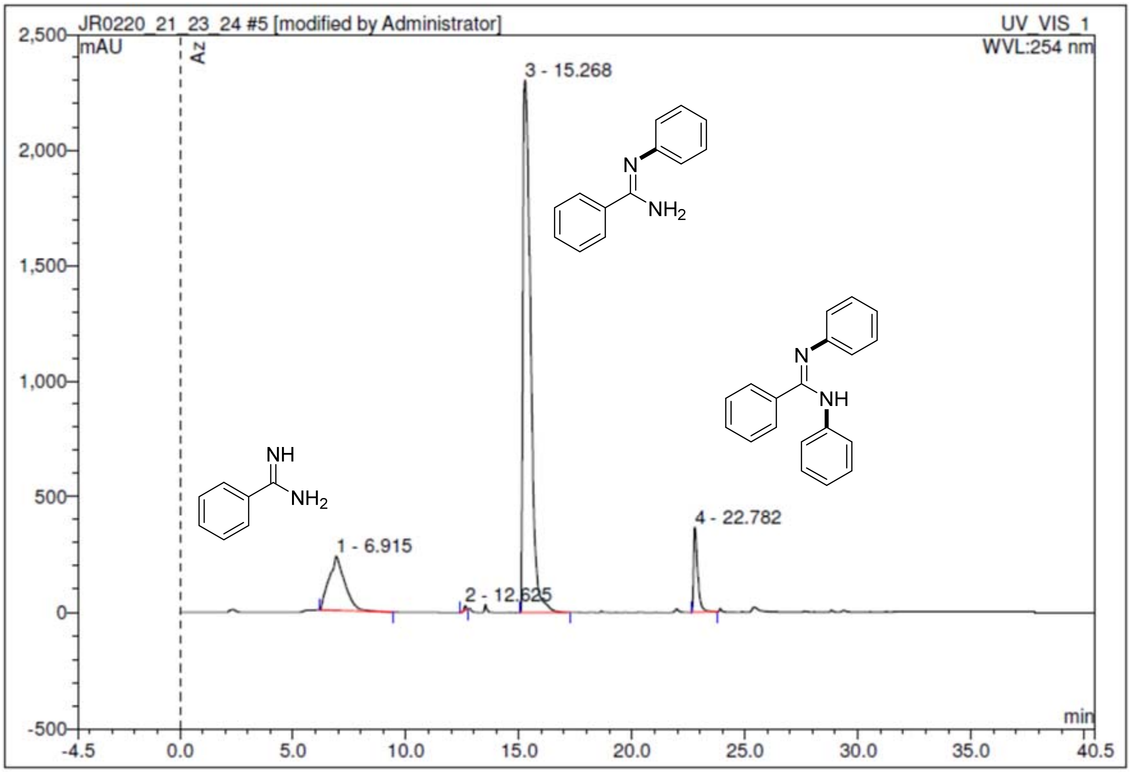

Figure S1. HPLC trace (Table **S4**, entry 21) showing starting material **1** (7.2 min), major product **2** (15.4 min) and minor product **3** (22.9 min).

EPR results

Results detailed in Figure S2 illustrate de-nucleation of the $Cu(OAc)_2$ dimer in the presence of benzamidine (green) to give a mononuclear Cu^{II} signal. $Cu(OAc)_2$ gives no signal (black) and addition of K_2CO_3 (red) or $PhB(OH)_2$ (blue) provides no de-nucleation. Mononuclear Cu^{II} signal again observed in the presence of amidine with K_2CO_3 and phenyl boronic acid (purple). Note precipitate formation for the sample containing all reagents shows a less intense signal (purple).

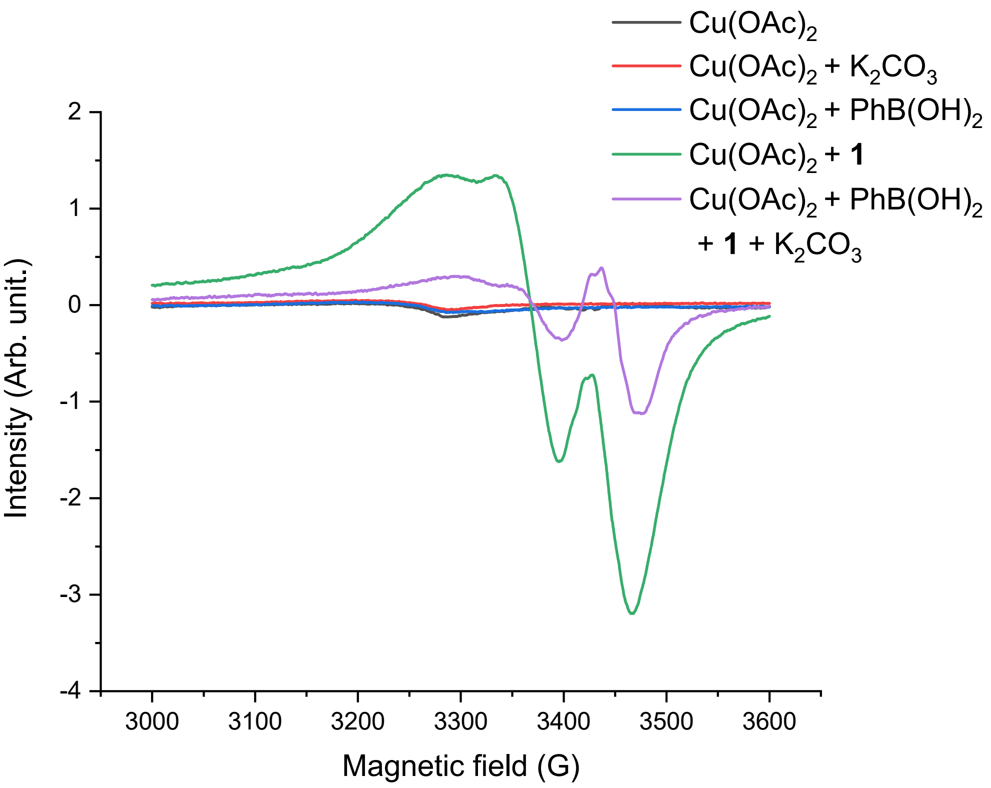

Figure S2. EPR spectra showing the de-nucleation of $Cu(OAc)_2$ with the different substrate combinations.

Precipitate formation in reactions

Images of precipitate formed during substoichiometric reactions.

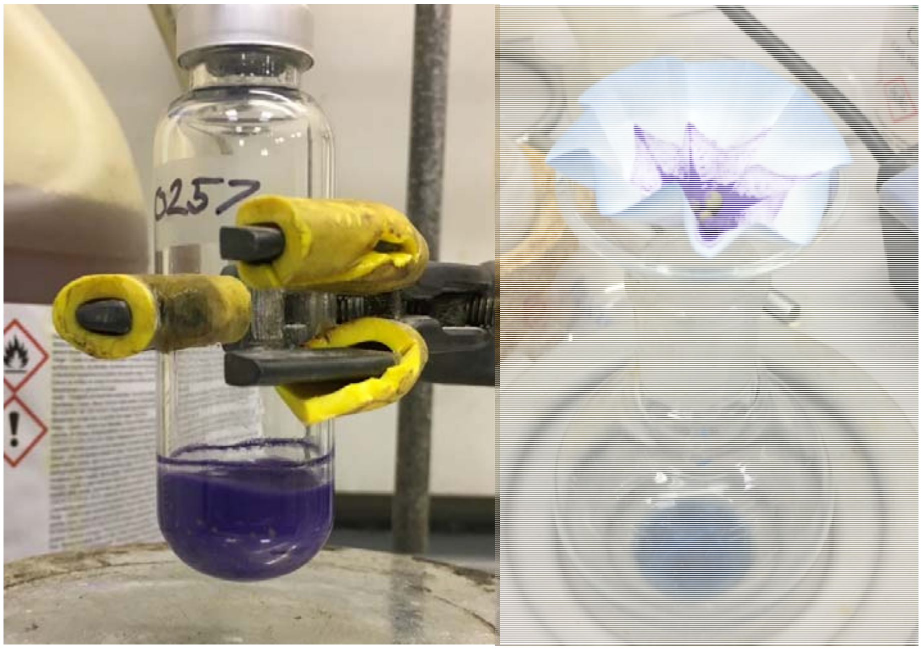

Figure S3. Purple precipitate formed during reactions using substoichiometric copper. Conditions: $Cu(OAc)_2$ (50 mol%), K_2CO_3 (2 equiv.), 4 Å MS, *i*-PrOH, RT, 2 h.

Compound characterisations

(*Z*)-*N*'-phenylbenzimidamide (**2**)

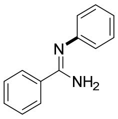

Chan-Lam procedure A (1.00 mmol scale boronic acid). Purification was carried out using a gradient of 0% to 50% EtOAc in petroleum ether (40-60) with 1% Et_3N modifier, affording a white powder (8 mg, 9 %).

Chan-Lam procedure B (1.00 mmol scale boronic acid). Purification was carried out using a gradient of 0% to 50% EtOAc in petroleum ether (40-60) with 1% Et_3N modifier, affording a white powder (162 mg, 83 %).

Chan-Lam procedure C (1.00 mmol scale boronic acid). Purification was carried out using a gradient of 0% to 50% EtOAc in petroleum ether (40-60)with 1% Et_3N modifier, affording a colourless solid (159 mg, 81 %).

mp 108-112 °C.

^{1}H NMR (500 MHz, $CDCl_3$): δ/ppm: 7.87 (d, 2H, *J* = 6.9 Hz, N=C_q-C_q-C*H* × 2), 7.43-7.51 (m, 3H, N=C_q-C_q-CH-C*H* × 2-C*H*), 7.35-7.39 (m, 2H, N-C_q-CH-C*H* × 2), 7.05-7.09 (m, 1H, N-C_q-CH-CH-C*H*), 7.00-7.02 (m, 2H, N-C_q-C*H* × 2), 4.85 (br s, 2H, N*H_2*).

$^{13}C\{^1H\}$ NMR (125 MHz, $CDCl_3$): δ/ppm: 154.2 (C_q), 151.1(C_q), 136.4 (C_q), 130.5 (*C*-H), 129.7 (2 × *C*-H), 128.4 (2 × *C*-H), 127.5 (2 × *C*-H), 122.3 (*C*-H), 122.0 (2 × *C*-H).

IR (neat): **ν_{max}/cm^{-1}**: 3565 (N-H stretch), 3344 (N-H stretch), 3051 (C-H stretch), 3040 (C-H stretch), 1615 (C=N stretch), 1589 (C=C stretch), 1569 (C=C stretch).

HRMS (ESI): Calc. for $C_{13}H_{13}N_2^+$. Theoretical: 197.1073 Observed: 197.1072.

(*Z*)-*N,N'*-diphenylbenzimidamide (**3**)

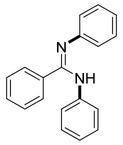

Chan-Lam procedure A (1.00 mmol scale boronic acid). Purification was carried out using a gradient of 0% to 60% EtOAc in petroleum ether (40-60) with 1% Et_3N modifier afforded a colourless solid (201 mg, 74 %).

Chan-Lam procedure B (1.00 mmol scale boronic acid). Purification was carried out using a gradient 0% to 50% EtOAc in petroleum ether (40-60) with 1% Et_3N modifier, affording a colourless solid (8 mg, 3%).

mp 140-143 °C.

^1H NMR (500 MHz, DMSO-d6): δ/ppm: 9.18 (br s, 1H, N*H*), 7.88 (d, 2H, *J* = 7.3 Hz, N=C_q-C_q-C*H* × 2), 7.26-7.33 (m, 7H, 7 × C*H*) 7.96-7.06 (m, 3H, 3 ×C*H*), 6.73-6.77 (m, 1H, N-C_q-CH-CH-C*H*), 6.58 (d, 2H, *J* = 7.1 Hz, N-C_q-C*H* × 2).

$^{13}C\{^1H\}$ NMR (125 MHz, DMSO-d6): δ/ppm: 154.2 (C_q), 150.6 (C_q), 141.3 (C_q), 134.7 (C_q), 128.9 (*C*-H), 128.9 (*C*-H), 128.8 (2 × *C*-H), 128.3 (2 × *C*-H), 127.9 (2 × *C*-H), 122.2 (2 × *C*-H), 121.8 (2 × *C*-H), 120.9 (*C*-H), 119.5 (2 × *C*-H).

IR (neat): **ν_{max}/cm^{-1}**: 3290 (N-H stretch), 3053 (C-H stretch), 3025 (C-H stretch), 1627 (C=N stretch), 1587 (C=C stretch), 1578 (C=C stretch), 1528 (C=C stretch).

HRMS (ESI): Calc. for $C_{19}H_{17}N_2^+$. Theoretical: 273.1386 Observed: 273.1387.

(*Z*)-*N'*-(4-fluorophenyl)benzimidamide (**7**)

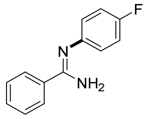

Chan-Lam procedure B (0.20 mmol scale boronic acid). Purification was carried out using a gradient 0% to 50% EtOAc in petroleum ether (40-60) with 1% Et_3N modifier, affording a white powder (27 mg, 63%)

Chan-Lam procedure C (0.40 mmol scale boronic acid). Purification was carried out using a gradient 0% to 50% EtOAc in petroleum ether (40-60) with 1% Et_3N modifier, affording a colourless solid (61 mg, 71%).

mp 113-117 °C.

^{1}H NMR (500 MHz, $CDCl_3$): δ/ppm: 7.84 (br s, 2H, N=C_q-C_q-C*H* × 2), 7.43-7.50 (m, 3H, N=C_q-C_q-CH-C*H* × 2-C*H*), 7.03-7.06 (m, 2H, F-C_q-C*H* × 2), 6.92-6.95 (m, 2H, F-C_q-CH-C*H* × 2), 4.84 (br s, 2H, NH_2).

^{13}C{^{1}H} NMR (125 MHz, $CDCl_3$): δ/ppm: 158.6 (d, *J* = 241.4 Hz, *C*-F), 154.9 (C_q), 144.9 (C_q), 135.0 (C_q), 130.2 (*C*-H), 128.1 (2 × *C*-H), 126.3 (2 × *C*-H), 122.3 (d, *J* = 7.9 Hz, 2 × *C*-H), 115.6 (d, *J* = 22.2 Hz, 2 × *C*-H).

^{19}F{^{1}H} NMR (470 MHz, $CDCl_3$): δ/ppm: -121.3.

IR (neat): **ν_{max} /cm^{-1}**: 3465 (N-H stretch), 3335 (N-H stretch), 3056 (C-H stretch), 1613 (C=N stretch), 1600 (C=C stretch), 1569 (C=C stretch), 1498 (C=C stretch).

HRMS (ESI): Calc. for $C_{13}H_{12}N_2F^+$. Theoretical: 215.0979 Observed: 215.0978.

(*Z*)-*N*'-(4-(methylthio)phenyl)benzimidamide (**8**)

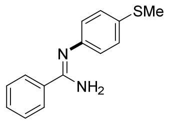

Chan-Lam procedure B (0.40 mmol scale boronic acid). Purification was carried out using a gradient 0% to 40% EtOAc in petroleum ether (40-60) with 1% Et_3N modifier, affording a colourless solid (69 mg, 71%).

Chan-Lam procedure C (0.04 mmol scale boronic acid). Purification was carried out using a gradient 0% to 40% EtOAc in petroleum ether (40-60) with 1% Et_3N modifier, affording a colourless solid (65 mg, 67%).

mp 123-127 °C.

^{1}H NMR (500 MHz, $CDCl_3$): δ/ppm: 7.82 (d, 2H, *J* = 6.8, N=C_q-C_q-C*H* × 2), 7.42-7.49 (m, 3H, N=C_q-C_q-CH-C*H* × 2-C*H*), 7.28 (d, 2H, *J* = 8.4 Hz, S-C_q-C*H* × 2), 6.93 (d, 2H, *J* = 8.4 Hz, S-C_q-CH-C*H* × 2), 4.85 (br s, 2H, NH_2) 2.48 (s, 3H, S-CH_3).

^{13}C{^{1}H} NMR (125 MHz, $CDCl_3$): δ 155.3 (C_q), 147.2 (C_q), 135.5 (C_q), 131.9 (C_q), 130.7 (*C*-H), 129.0 (2 × *C*-H), 128.6 (2 × *C*-H), 126.8 (2 × *C*-H), 122.4 (2 × *C*-H), 17.0 (S-*C*H_3).

IR (neat): **ν_{max}/cm^{-1}**: 3465 (N-H stretch), 3324 (N-H stretch), 2915 (C-H alkyl stretch), 1621 (C=N stretch), 1600 (C=C stretch), 1589 (C=C stretch), 1558 9C=C stretch).

HRMS (ESI): Calc. for $C_{14}H_{15}N_2S^+$. Theoretical: 243.0950 Observed: 243.0945.

(*Z*)-*N*'-(4-(tert-butyl)phenyl)benzimidamide (**9**)

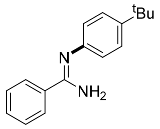

Chan-Lam procedure B (0.20 mmol scale boronic acid). Purification was carried out using a gradient 0% to 50% EtOAc in petroleum ether (40-60) with 1% Et_3N modifier, affording an off-white powder (41 mg, 82%).

mp 153-155 °C.

^{1}H NMR (500MHz, $CDCl_3$): δ/ppm: 7.86 (d, 2H, *J* = 5.0 Hz, N=C_q-C_q-C*H* × 2), 7.42-7.49 (m, 3H, N=C_q-C_q-CH-C*H* × 2-C*H*) 7.35 (d, 2H, *J* = 8.5 Hz, ^{t}Bu-C_q- C*H* × 2), 6.92 (d, 2H, *J* = 8.5 Hz, ^{t}Bu-C_q-CH-C*H* × 2), 4.92 (br s, 2H, N*H*$_2$), 1.32 (s, 9H, C*H*$_3$ × 3).

^{13}C{^{1}H} NMR (125 MHz, $CDCl_3$): δ /ppm: 154.9 (C_q), 145.5 (C_q), 135.1 (C_q), 130.1 (*C*-H), 128.1 (2 × *C*-H), 126.4 (2 × *C*-H), 125.8 (2 × *C*-H), 122.3 (C_q), 120.7 (2 × *C*-H), 33.8 (C_q), 30.9 (3 × *C*H$_3$).

IR (neat): **ν_{max}/cm^{-1}**: 3439 (N-H stretch), 3121 (N-H stretch), 3054 (C-H stretch), 2898 (C-H alkyl stretch), 2863 (C-H alkyl stretch), 1632 (C=N stretch), 1597 (C=C stretch), 1571 (C=C stretch).

HRMS (ESI): Calc. for $C_{17}H_{21}N_2^+$. Theoretical : 253.1699 Observed : 253.1701.

(*Z*)-*N*'-(4-(trifluoromethyl)phenyl)benzimidamide (**10**)

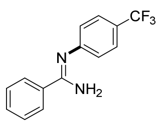

Chan-Lam procedure B (0.40 mmol scale boronic acid). Purification was carried out using a gradient 0% to 50% EtOAc in petroleum ether (40-60) with 1% Et_3N modifier, affording a colourless solid (68 mg, 64%).

mp 143-145 °C.

^{1}H NMR (500 MHz, $CDCl_3$): δ/ppm: 7.86 (br s, 2H, N=C_q-C_q-C*H* × 2), 7.61 (d, 2H, *J* = 8.2 Hz, F_3C-C_q- C*H* × 2), 7.44-7.53 (m, 3H, N=C_q-C_q-CH-C*H* × 2-C*H*), 7.08 (d, 2H, *J* = 8.2, F_3C-C_q-CH-C*H* × 2), 4.86 (br s, 2H, N*H*$_2$).

^{13}C{^{1}H} NMR (101 MHz, $CDCl_3$): δ/ppm: 155.1 (C_q), 152.1 (C_q), 134.4 (C_q), 130.6 (*C*-H), 128.2 (2 × *C*-H), 126.4 (2 × *C*-H), 126.2 (q, *J* = 3.5 Hz, 2 × *C*-H), 124.7 (q, *J* = 32.9, C_q), 124.0 (q, *J* = 270.6 Hz, *C*F$_3$), 121.5 (2 × *C*-H).

^{19}F{^{1}H} NMR (376 MHz, $CDCl_3$): δ/ppm: -61.8.

IR (neat): **ν_{max} /cm^{-1}**: 3463 (N-H stretch), 3330 (N-H stretch), 3049 (C-H stretch), 1619 (C=N stretch), 1599 (C=C stretch), 1573 (C=C stretch).

HRMS (ESI): Calc. for $C_{14}H_{11}N_2F_3^+$. Theoretical: 265.0947 Observed: 265.0947.

(*Z*)-*N*'-(*m*-tolyl)benzimidamide (**11**)

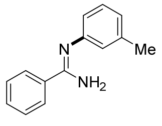

Chan-Lam procedure B (0.40 mmol scale boronic acid). Purification was carried out using a gradient 0% to 50% EtOAc in petroleum ether (40-60) with 1% Et_3N modifier, affording a colourless solid (51 mg, 61%).

mp 98-102 °C.

^{1}H NMR (500 MHz, $CDCl_3$): δ/ppm: 7.86 (d, 2H, *J* = 7.0 Hz, N=C_q-C_q-C*H* × 2), 7.42-7.49 (m, 3H, N=C_q-C_q-CH-C*H* × 2-C*H*) 7.22-7.25 (m, 1H, N-C_q-CH-C*H*), 6.88 (d, 1H, *J* = 7.5 Hz, N-C_q-CH-CH-C*H*), 6.83 (s, 1H, N-C_q-C*H*-C_q-CH$_3$), 6.79 (d, 1H, *J* = 7.9 Hz, N-C_q-C*H*-CH) 4.84 (br s, 2H, N*H*$_2$), 2.35 (s, 3H, C*H*$_3$).

^{13}C{^{1}H} NMR (125 MHz, $CDCl_3$): δ/ppm 154.4 (C_q), 148.9 (C_q), 138.8 (C_q), 135.3 (C_q), 130.1 (*C*-H), 128.8 (*C*-H), 128.0 (2 × *C*-H), 126.3 (2 × *C*-H), 123.4 (*C*-H), 121.8 (*C*-H), 118.1 (*C*-H), 21.0 (*C*H$_3$).

IR (neat): $\mathbf{v_{max}/cm^{-1}}$: 3434 (N-H stretch), 3285 (N-H stretch), 3127 (C-H stretch), 1625 (C=N stretch), 1604 (C=C stretch), 1591 (C=C stretch), 1571 (C=C stretch).

HRMS (ESI): Calc. for $C_{14}H_{15}N_2^+$. Theoretical: 211.1230 Observed: 211.1224.

(*Z*)-*N'*-([1,1'-biphenyl]-4-yl)benzimidamide (**12**)

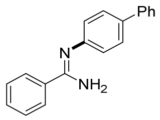

Chan-Lam procedure B (1.00 mmol scale boronic acid). Purification was carried out using a gradient 0% to 50% EtOAc in petroleum ether (40-60) with 1% Et_3N modifier, affording an off-white powder (188 mg, 69%).

mp 173-177 °C.

^{1}H NMR (500 MHz, $CDCl_3$): δ/ppm: 7.88 (d, 2H, *J* = 5.8 Hz, N=C_q-C_q-C*H* × 2), 7.60-7.62 (m, 4H, 4 × C*H*), 7.42-7.51 (m, 5H, 5 × C*H*), 7.31-7.34 (m, 1H, N-C_q-CH-CH-C_q-CH-CH-C*H*), 7.07 (d, 2H, *J* = 8.4 Hz, 2 × C*H*), 4.91 (br s, 2H, N*H*$_2$).

^{13}C{^{1}H} NMR (125 MHz, $CDCl_3$): δ/ppm: 155.0 (C_q), 148.9 (C_q), 141.0 (C_q), 135.9 (C_q), 135.7 (C_q), 130.7 (*C*-H), 128.8 (2 × *C*-H), 128.6 (2 × *C*-H), 128.2 (2 × *C*-H), 126.9 (*C*-H), 126.8 (2 × *C*-H), 126.7 (2 × *C*-H), 122.1 (2 × *C*-H).

IR (neat): $\mathbf{v_{max}/cm^{-1}}$: 3463 (N-H stretch), 3344 (N-H stretch), 3056 (C-H stretch), 3028 (C-H stretch), 1610 (C=N stretch), 1589 (C=C stretch), 1569 (C=C stretch).

HRMS (ESI): Calc. for $C_{19}H_{17}N_2^+$. Theoretical: 273.1386 Observed: 273.1386.

(*Z*)-*N'*-(*o*-tolyl)benzimidamide (**13**)

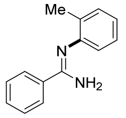

Chan-Lam procedure B (0.20 mmol scale boronic acid). Purification was carried out using a gradient 0% to 50% EtOAc in petroleum ether (40-60) with 1% Et_3N modifier, affording an off-white powder (17 mg, 40%).

Chan-Lam procedure C (0.40 mmol scale boronic acid). Purification was carried out using a gradient 0% to 50% EtOAc in petroleum ether (40-60) with 1% Et_3N modifier, affording an off-white powder (40 mg, 48%).

mp 115-118 °C.

^{1}H NMR (500 MHz, $CDCl_3$): δ/ppm: 7.89 (br s, 2H, N=C_q-C_q-C*H* × 2), 7.43-7.50 (m, 3H, N=C_q-C_q-CH-C*H* × 2-C*H*), 7.22 (d, 1H, *J* = 7.4, N-C_q-C_q-C*H*), 7.16-7.19 (m, 1H, N-C_q-CH-C*H*), 6.97-7.00 (m, 1H, N-C_q-C_q-CH-C*H*), 6.88 (d, 1H, *J* = 7.4 Hz, N-C_q-C*H*), 4.71 (br s, 2H, N*H*$_2$), 2.22 (s, 3H, C*H*$_3$).

^{13}C{^{1}H} NMR (125 MHz, $CDCl_3$): δ/ppm: 153.0 (C_q), 147.0 (C_q), 134.7 (C_q), 129.8 (*C*-H), 129.7 (*C*-H), 128.7 (C_q), 127.6 (2 × *C*-H), 125.9 (2 × *C*-H), 125.8 (*C*-H), 122.3 (*C*-H), 120.2 (*C*-H), 16.7 (*C*H$_3$).

IR (neat): $\mathbf{v_{max}/cm^{-1}}$: 3436 (N-H stretch), 3162 (N-H stretch), 3056 (C-H stretch), 2917(C-H alkyl stretch), 1630 (C=N stretch), 1595 (C=C stretch), 1563 (C=N stretch).

HRMS (ESI): Calc. for $C_{14}H_{15}N_2^+$. Theoretical: 211.1230 Observed: 211.1229.

(*Z*)-*N*'-(2-methoxyphenyl)benzimidamide (**14**)

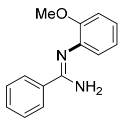

Chan-Lam procedure B (0.20 mmol scale boronic acid). Purification was carried out using a gradient 0% to 50% EtOAc in petroleum ether (40-60) with 1% Et_3N modifier, affording an off-white powder (39 mg, 42%).

mp 109-113 °C.

^{1}H NMR (500 MHz, $CDCl_3$): δ/ppm: 7.86 (d, 2H, *J* = 6.2 Hz, N=C_q-C_q-C*H* × 2), 7.42-7.50 (m, 3H, N=C_q-C_q-CH-C*H* × 2-C*H*), 7.19 (br s, 1H, N-C_q-C*H*), 7.03-7.08 (m, 1H, N-C_q-C_q-CH-C*H*), 6.94-6.99 (m, 2H, N-C_q-CH-C*H,* N-C_q-C_q-C*H*), 4.72 (br s, 2H, N*H₂*), 3.83 (s, 3H, O-C*H*₃).

^{13}C{^{1}H} NMR (125 MHz, $CDCl_3$): δ/ppm: 154.0 (C_q), 151.3 (C_q), 139.8 (C_q), 136.3(C_q), 130.4 (*C*-H), 128.4 (2 × *C*-H), 127.6 (2 × *C*-H), 123.2 (*C*-H), 122.9 (*C*-H), 121.5 (*C*-H), 112.8 (*C*-H), 55.8 (O-*C*H₃).

IR (neat) ν_{max} (neat): 3434 (N-H stretch), 3294 (N-H stretch), 3054 (C-H stretch), 2826 (C-H alkyl stretch), 1640 (C=N stretch), 1569 (C=C stretch).

HRMS (ESI): $C_{14}H_{15}N_2O^+$. Theoretical: 227.1179 Observed: 227.1171.

(*Z*)-*N*'-(pyridin-3-yl)benzimidamide trifluoroacetate (**15**)

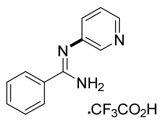

Chan-Lam procedure B (0.40 mmol scale boronic acid). Purification was carried out using RP-HPLC method A, affording a white amorphous solid (47 mg, 38%).

^{1}H NMR (500 MHz, DMSO-*d₆*): δ/ppm: 8.19 (s, 1H, N=C*H*-CH), 8.13, (s, 1H, N=C*H*-C_q), 7.97 (d, 2H, *J* = 6.9 Hz, N=C_q-C_q-C*H* × 2), 7.45-7.49 (m, 3H, N=C_q-C_q-CH-C*H* × 2-C*H*), 7.26-7.49 (m, 2H, N-C_q-C*H*-C*H*), 6.59 (br s, 2H, N*H*₂).

^{13}C{^{1}H} NMR (125 MHz, DMSO-*d₆*): δ/ppm: 155.2 (C_q), 146.5 (*C*-H), 143.6 (C_q), 142.8 (*C*-H), 135.5 (C_q), 130.3 (*C*-H), 128.7 (*C*-H), 128.0 (2 × *C*-H), 127.1 (2 × *C*-H), 123.9 (*C*-H).

^{19}F{^{1}H} NMR (470 MHz, DMSO-d₆): δ/ppm: -73.5.

IR (neat): **ν_{max}/cm^{-1}**: 3376 (N-H stretch), 3318 (N-H stretch), 3185 (C-H stretch), 1647 (C=N stretch), 1610 (C=N stretch), 1573 (C=C stretch).

HRMS (ESI): Calc. for $C_{12}H_{12}N_3^+$. Theoretical: 198.1026 Observed: 198.1022.

(*Z*)-*N*'-(3-fluoro-4-methoxyphenyl)benzimidamide (**16**)

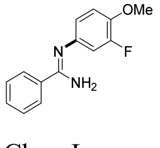

Chan-Lam procedure B (0.20 mmol scale boronic acid). Purification was carried out using a gradient

0% to 50% EtOAc in petroleum ether (40-60) with 1% Et_3N modifier, affording a colourless solid (67 mg, 68%).

mp 119-123 °C.

^{1}H NMR (500 MHz, $CDCl_3$): δ/ppm: 7.83 (br s, 2H, N=C_q-C_q-C*H* × 2), 7.43-7.50 (m, 3H, N=C_q-C_q-CH-C*H* × 2-C*H*), 6.95 (m, 1H, N-C_q-C*H*-CH), 6.78 (d, 1H, *J* = 12.4 Hz, N-C_q-C*H*-CF), 6.70 (d, 1H, *J* = 8.5 Hz N-C_q-CH-C*H*), 4.91 (br s, 2H, NH_2) 3.88 (s, 3H, O-CH_3).

^{13}C{^{1}H} NMR (125 MHz, $CDCl_3$): δ/ppm: 155.7 (C_q), 153.0 (d, *J* = 246.8 Hz, C-F), 143.5 (d, *J* = 10.8, C_q), 143.0 (C_q), 135.5 (C_q), 130.7 (*C*-H), 128.6 (2 × *C*-H), 126.8 (2 × *C*-H), 117.1 (*C*-H), 114.7 (d, *J* = 2.1Hz, *C*-H), 110.2 (d, *J* = 18.9 Hz, *C*-H), 56.7 (O-*C*H_3).

^{19}F{^{1}H} NMR (376 MHz, $CDCl_3$): δ/ppm: -133.5.

IR (neat): **ν_{max}/cm^{-1}**: 3458 (N-H stretch), 3287 (N-H stretch), 3135 (C-H stretch), 3017 (C-H stretch), 2842 (C-H alkyl stretch), 1627 (C=N stretch), 1593 (C=C stretch), 1569 (C=C stretch).

HRMS (ESI): Calc. for $C_{14}H_{14}N_2FO^+$. Theoretical: 245.1085 Observed: 245.1078.

(*Z*)-*N*'-(3-methoxyphenyl)benzimidamide (**17**)

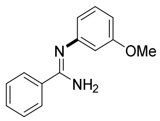

Chan-Lam procedure B (0.20 mmol scale boronic acid). Purification was carried out using a gradient 0% to 50% EtOAc in petroleum ether (40-60) with 1% Et_3N modifier, affording a colourless solid (28 mg, 62%).

mp 108-112 °C.

^{1}H NMR (500 MHz, $CDCl_3$): δ/ppm: 7.86 (d, 2H, *J* = 5.4 Hz, N=C_q-C_q-C*H* × 2), 7.43-7.50 (m, 3H, N=C_q-C_q-CH-C*H* × 2-C*H*) 7.24-7.27 (m, 1H, , N-C_q-CH-C*H*), 6.63 (dd, 1H, *J* = 8.4 Hz, 2.3 Hz, N-C_q-CH-CH-C*H*), 6.56-6.59 (m, 2H, N-C_q-C*H* × 2,) 4.89 (br s, 2H, NH_2), 3.80 (s, 3H, CH_3).

^{13}C{^{1}H} NMR (125 MHz, $CDCl_3$): δ/ppm: 160.3 (C_q), 154.6 (C_q), 150.4 (C_q), 135.11 (C_q), 130.2 (*C*-H), 129.8 (*C*-H), 128.1 (2 × *C*-H), 126.3 (2 × *C*-H), 113.4 (*C*-H), 108.6 (*C*-H), 106.7 (*C*-H), 54.7 (O-*C*H_3).

IR (neat): **ν_{max}/cm^{-1}**: 3432 (N-H stretch), 3279 (N-H stretch), 3095 (C-H stretch), 2934 (C-H alkyl stretch), 2831 (C-H alkyl stretch), 1636 (C=N stretch), 1587 (C=C stretch), 1573 (C=C stretch).

HRMS (ESI): Calc. for $C_{14}H_{15}N_2O^+$. Theoretical: 227.1178 Observed: 227.1179.

(*Z*)-*N*'-(4-methoxy-3,5-dimethylphenyl)benzimidamide (**18**)

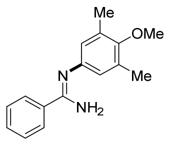

Chan-Lam procedure B (0.40 mmol scale boronic acid). Purification was carried out using a gradient 0% to 30% EtOAc in petroleum ether (40-60) with 1% Et_3N modifier, affording an off-white powder. (85 mg, 83%).

mp 140-143 °C.

^{1}H NMR (500 MHz, $CDCl_3$): δ/ppm: 7.84 (d, 2H, *J* = 6.9 Hz, N=C_q-C_q-C*H* × 2), 7.41-7.47 (m, 3H, N=C_q-C_q-CH-C*H* × 2-C*H*), 6.64 (s, 2H, N-C_q-C*H* × 2), 4.86 (br s, 2H, NH_2), 3.72 (s, 3H, O-CH_3), 2.27 (s, 6H, C_q-CH_3 × 2).

^{13}C{^{1}H} NMR (125 MHz, $CDCl_3$): δ/ppm: 154.2 (C_q), 152.3 (C_q), 144.7 (C_q), 135.5 (C_q), 131.3 (C_q), 130.0 (*C*-H), 128.0 (2 × *C*-H), 126.2 (2 × *C*-H), 121.0 (2 × *C*-H), 59.3 (O-CH_3), 15.7 (2 × CH_3).

IR (neat) ν_{max}/cm^{-1}: 3413 (N-H stretch), 3341 (N-H stretch), 3207 (C-H stretch), 2930 (C-H alkyl stretch), 2921 (C-H alkyl stretch), 2854 (C-H alkyl stretch), 1638 (C=N stretch), 1569 (C=C stretch).

HRMS (ESI): Calc. for $C_{16}H_{19}N_2O^+$. Theoretical: 255.1492 Observed: 255.1487.

(*Z*)-*N*'-(3,4,5-trifluorophenyl)benzimidamide (**19**)

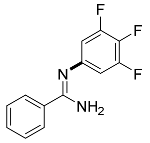

Chan-Lam procedure B (0.40 mmol scale boronic acid). Purification was carried out using a gradient 0% to 30% EtOAc in petroleum ether (40-60) with 1% Et_3N modifier, affording an off-white powder (57 mg, 57%).

mp 108-110 °C.

^{1}H NMR (500 MHz, $CDCl_3$): δ/ppm: 7.79 (br s, 2H, N=C_q-C_q-C*H* × 2), 7.50-7.53 (m, 1H, N=C_q-C_q-CH-CH-C*H*), 7.44-7.47 (m, 2H, N=C_q-C_q-CH-C*H* × 2), 6.58-6.62 (m, 2H, N-C_q-C*H* × 2), 5.99 (br s, 2H, NH_2).

^{13}C{^{1}H} NMR (125 MHz, $CDCl_3$): δ/ppm: 154.5 (C_q), 150.9 (ddd, *J* = 248.4 Hz, 10.5 Hz, 4.9 Hz, 2 × *C*-F), 144.6 (ddd, *J* = 13.4 Hz, 9.1 Hz, 3.6 Hz, *C*-F), 135.9 (t, *J* = 15.8 Hz, C_q), 134.0 (C_q), 130.1 (*C*-H), 127.7 (2 × *C*-H), 125.7 (2 × *C*-H), 104.8 (dd, *J* = 16.7 Hz, 4.9 Hz, 2 × *C*-H).

^{19}F{^{1}H} NMR (470 MHz, $CDCl_3$): δ/ppm: -133.9 (d, *J* = 19.2 Hz), -168.2 (t, *J* = 19.2 Hz).

IR (neat): **ν_{max}/cm^{-1}**: 3426 (N-H stretch), 3277 (N-H stretch), 3116 (C-H stretch), 1636 (C=N stretch) 1574 (C=C stretch), 1517 (C=C stretch).

HRMS (ESI): Calc. for $C_{13}H_{10}F_3N_2^+$. Theoretical: 251.0791 Observed: 251.0782.

(*Z*)-*N*'-((*E*)-styryl)benzimidamide (**20**)

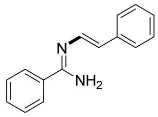

Chan-Lam procedure B (0.40 mmol scale boronic acid). Purification was carried out using a gradient 0% to 50% EtOAc in petroleum ether (40-60) with 1% Et_3N modifier, affording a pale-yellow powder (31 mg, 35 %).

mp 117-121°C

^{1}H NMR (500 MHz, DMSO-d6): δ/ppm: 7.91 (d, 2H, *J* = 6.9 Hz, N=C_q-C_q-C*H* × 2), 7.86 (d, 1H, *J* = 13.4 Hz, N-CH=C*H*-C_q), 7.44-7.46 (m, 2H, N-CH=CH-C_q-C*H* × 2), 7.38-7.43 (m, 3H, N=C_q-C_q-CH-C*H* × 2-C*H*), 7.24-7.27 (m, 2H, N-CH=CH-C_q-CH-C*H* × 2), 7.07-7.13 (m, 3H, NH_2, CH=CH-C_q-CH-

CH-C*H*), 6.32 (d, 1H, J = 13.4 Hz, N-C*H*=CH-C_q).

$^{13}C\{^1H\}$ NMR (125 MHz, DMSO-d6): δ/ppm: 155.6 (C_q), 138.9 (C_q), 136.6 (C_q), 136.0 (*C*-H), 130.4 (*C*-H), 128.9 (2 × *C*-H), 128.5 (2 × *C*-H), 127.4 (2 × *C*-H), 126.0 (*C*-H), 125.9 (2 × *C*-H), 121.2 (*C*-H).

IR (neat): **ν_{max}/cm^{-1}**: 3430 (N-H stretch), 3285 (N-H stretch), 3054 (C-H stretch), 3019 (C-H stretch), 1627 (C=N stretch), 1587 (C=C stretch), 1548 (C=C stretch).

HRMS (ESI): Calc. for $C_{15}H_{15}N_2^+$. Theoretical: 223.1230 Observed: 223.1225.

(*Z*)-*N*'-(cyclohex-1-en-1-yl)benzimidamide (**21**)

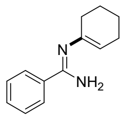

Chan-Lam procedure B (1.0 mmol scale boronic acid). Purification was carried out using a gradient 0% to 30% EtOAc in petroleum ether (40-60) with 1% Et_3N modifier, affording an off-white powder (83 mg, 41%).

Chan-Lam procedure C (1.00 mmol scale boronic acid). Purification was carried out using a gradient 0% to 30% EtOAc in petroleum ether (40-60) with 1% Et_3N modifier, affording a white powder (66 mg, 33%).

mp 103-105 °C.

^1H NMR (500 MHz,$CDCl_3$): δ/ppm: 7.76 (d, 2H, J = 7.1 Hz, N=C_q-C_q-C*H* × 2), 7.37-7.44 (m, 3H, N=C_q-C_q-CH-C*H* × 2-C*H*), 5.14 (br s, 1H, N-C_q=C*H*), 5.00 (br s, 2H, N*H*$_2$), 2.10-2.13 (m, 4H, N-C_q=CH-C*H*$_2$, N-C_q-C*H*$_2$), 1.72-1.77 (m, 2H, N-C_q-CH_2-C*H*$_2$), 1.60-1.65 (m, 2H, N-C_q-CH_2-CH_2-C*H*$_2$).

$^{13}C\{^1H\}$ NMR (125 MHz, $CDCl_3$): δ/ppm: 153.2 (C_q), 144.0 (C_q), 135.7 (C_q), 129.7 (*C*-H), 127.9 (2 × *C*-H), 126.1 (2 × *C*-H), 109.5 (*C*-H), 27.5 (*C*H_2), 23.9 (*C*H_2), 22.6 (*C*H_2), 22.0 (*C*H_2).

IR (neat): **ν_{max}/cm^{-1}**: 3458 (N-H stretch), 3292 (N-H stretch), 3025 (C-H stretch), 2928 (C-H alkyl stretch), 2909 (C-H alkyl stretch), 2850 (C-H alkyl stretch), 2831 (C-H alkyl stretch), 1651 (C=N stretch), 1574 (C=C stretch).

HRMS (ESI): Calc. for $C_{13}H_{17}N_2^+$. Theoretical: 201.1386 Observed: 201.1381.

(*Z*)-4-chloro-*N*'-phenylbenzimidamide (**24**)

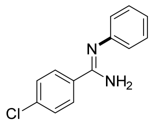

Chan-Lam procedure B (0.40 mmol scale boronic acid). Purification was carried out using a gradient 0% to 40% EtOAc in petroleum ether (40-60) with 1% Et_3N modifier, affording an off-white powder (56 mg, 61%).

Chan-Lam procedure C (0.40 mmol scale boronic acid). Purification was carried out using a gradient 0% to 40% EtOAc in petroleum ether (40-60) with 1% Et_3N modifier, affording a white powder (63 mg, 69 %).

mp 129-132 °C.

^{1}H NMR (500 MHz, $CDCl_3$): δ/ppm: 7.78 (d, 2H, *J* = 7.6 Hz, N=C_q-C_q-C*H* × 2), 7.41 (m, 2H, Cl-C_q-C*H* × 2) 7.34-7.37 (m, 2H, N-C_q-CH-C*H* × 2), 7.06-7.09 (m, 1H, N-C_q-CH-CH-C*H*), 6.96 (d, 2H, *J* = 7.4 Hz, N-C_q-C*H* × 2) 4.80 (br s, 2H, N*H*$_2$).

^{13}C{^{1}H} NMR (125 MHz, $CDCl_3$): δ/ppm: 154.3 (C_q), 149.1 (C_q), 136.8 (C_q), 134.0 (C_q), 129.6 (2 × *C*-H), 128.8 (2 × *C*-H), 128.3 (*C*-H), 123.4 (2 × *C*-H), 121.6 (2 × *C*-H).

IR (neat): **ν_{max}/cm^{-1}**: 3456 (N-H stretch), 3304 (N-H stretch), 3067 (C-H stretch), 3026 (C-H stretch), 1621 (C=N stretch), 1591 (C=C stretch), 1582 (C=C stretch), 1558 (C=C stretch).

HRMS (ESI): $C_{13}H_{12}ClN_2^+$. Theoretical: 231.0684 Observed: 231.0677.

(*Z*)-3-methoxy-*N*'-phenylbenzimidamide (**25**)

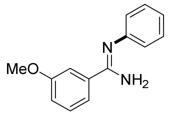

Using HCl salt of amidine following Chan-Lam procedure B (0.40 mmol scale boronic acid). Purification was carried out using a gradient 0% to 50% EtOAc in petroleum ether (40-60) with 1% Et_3N modifier, affording an off-white powder (48 mg, 53%).

mp 104-107 °C.

^{1}H NMR (500 MHz, $CDCl_3$): δ/ppm: 7.43 (br s, 1H, N=C_q-C_q-C*H*-CH), 7.32-7.37 (m, 4H, 4 × C*H*), 7.05-7.09 (m, 1H, N-C_q-CH-CH-C*H*), 6.98-7.04 (m, 3H, 3 × C*H*), 5.06 (br s, 2H, N*H*$_2$), 3.86 (s, 3H, O-C*H*$_3$).

^{13}C{^{1}H} NMR (125 MHz, $CDCl_3$): δ/ppm: 159.3 (C_q), 154.8 (C_q), 148.2 (C_q), 136.3 (C_q), 129.1 (2 × *C*-H), 129.0 (*C*-H), 122.8 (2 × *C*-H), 121.3 (*C*-H), 118.5 (*C*-H), 116.6 (*C*-H), 111.6 (*C*-H), 55.0 (O-*C*H$_3$).

IR (neat): **ν_{max}/cm^{-1}**: 3465 (N-H stretch), 3335 (N-H stretch), 3056 (C-H stretch), 2922 (C-H alkyl stretch), 2850 (C-H alkyl stretch), 2829 (C-H alkyl stretch), 1621 (C=N stretch), 1600 (C=C stretch), 1589 (C=C stretch), 1574 (C=C stretch).

HRMS (ESI): Calc. for $C_{14}H_{15}N_2O^+$. Theoretical: 227.1179 Observed: 227.1226.

(*Z*)-2-fluoro-*N*'-phenylbenzimidamide (**26**)

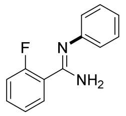

Chan-Lam procedure B (0.40 mmol scale boronic acid). Purification was carried out using a gradient 0% to 50% EtOAc in petroleum ether (40-60) with 1% Et_3N modifier, affording a colourless solid (45 mg, 52%).

mp 95-99 °C.

^{1}H NMR (500 MHz, $CDCl_3$): δ/ppm: 8.09 (br s, 1H, N=C_q-C_q-C*H*), 7.40-7.46 (m, 1H, F-C_q-C*H*), 7.33-7.37 (m, 2H, N-C_q-CH-C*H* × 2), 7.22-7.26 (m, 1H, F-C_q-CH-C*H*), 7.09-7.14 (m, 1H, F-C_q-CH-CH-C*H*), 7.05-7.08 (m, 1H, N-C_q-CH-CH-C*H*), 6.98 (d, 2H, *J* = 7.4 Hz, N-C_q-C*H* × 2), 5.16 (br s, 2H, N*H*$_2$).

$^{13}C\{^{1}H\}$ NMR (125 MHz, $CDCl_3$): δ/ppm: 160.1 (d, *J* = 248.1 Hz, *C*-F) 151.4 (C_q), 148.1 (C_q), 131.6 (d, *J* = 9.1 Hz, *C*-H), 130.6 (d, *J* = 1.3 Hz, *C*-H), 129.1 (2 × *C*-H), 124.1 (d, *J* = 3.4 Hz, *C*-H), 122.7 (2 × *C*-H), 122.2 (d, *J* = 10.1 Hz, C_q), 121.2 (*C*-H), 115.6 (d, *J* = 23.6 Hz, *C*-H).

$^{19}F\{^{1}H\}$ NMR (470 MHz, $CDCl_3$): δ/ppm: -115.2.

IR (neat): ν_{max}/cm^{-1}: 3428 (N-H stretch), 3277 (N-H stretch), 3028 (C-H stretch), 1632 (C=N stretch), 1619 (C=N stretch), 1606 (C=C stretch), 1589 (C=C stretch).

HRMS (ESI): Calc. for $C_{13}H_{12}FN_2^+$. Theoretical: 215.0979 Observed: 215.0970.

(*Z*)-3-bromo-*N*'-phenylbenzimidamide (**27**)

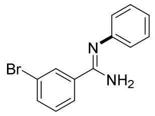

Chan-Lam procedure B using corresponding amidine HCl salt (1.00 mmol scale boronic acid). Purification was carried out using a gradient 0% to 50% EtOAc in petroleum ether (40-60) with 1% Et_3N modifier, affording an off-white powder (133 mg, 49 %).

mp 103-106 °C.

^{1}H NMR (500 MHz, $CDCl_3$): δ/ppm: 8.03 (br s, 1H, Br-C_q-C*H*-C_q), 7.73 (d, 1H, *J* = 7.2 Hz, N=C_q-C_q-C*H*-CH), 7.56 (d, 1H, *J* = 7.9 Hz, Br-C_q-C*H*-CH), 7.34-7.37 (m, 2H, N-C_q-CH-C*H* × 2), 7.27-7.30 (m, 1H, Br-C_q-CH-C*H*), 7.06-7.09 (m, 1H, N-C_q-CH-CH-C*H*), 6.96 (2H, d, *J* = 7.2 Hz, N-C_q-C*H* × 2), 4.88 (2H, br s, NH_2).

$^{13}C\{H\}$NMR (125 MHz, $CDCl_3$): δ/ppm: 153.5 (C_q), 149.2 (C_q), 137.9 (C_q), 133.5 (*C*-H), 130.2 (*C*-H), 130.0 (*C*-H), 129.6 (2 × *C*-H), 125.4, 123.3 (*C*-H), 122.7 (C_q), 121.5 (2 × *C*-H).

IR (neat): ν_{max}/cm^{-1}: 3434 (N-H stretch), 3276 (N-H stretch), 3110 (N-H stretch), 3056 (C-H stretch), 1634 (C=N stretch), 1604 (C=C stretch), 1586 (C=C stretch), 1558 (C=C stretch).

HRMS (ESI): Calc. for $C_{13}H_{12}BrN_2^+$. Theoretical: 275.0178 Observed: 275.0177.

(*Z*)-4-methyl-*N*'-phenylbenzimidamide (**28**)

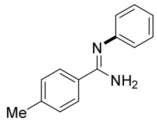

Chan-Lam procedure B (0.40 mmol scale boronic acid). Purification was carried out using a gradient 0% to 50% EtOAc in petroleum ether (40-60) with 1% Et_3N modifier, affording a colourless solid (26 mg, 31%).

mp 130-133 °C.

^{1}H NMR (500 MHz, $CDCl_3$): δ/ppm: 7.72 (d, 2H, *J* = 6.6 Hz, N=C_q-C_q-C*H* × 2), 7.32-7.35 (m, 2H, N-C_q-CH-C*H* × 2) 7.24 (m, 2H, H_3C-C_q-C*H* × 2), 7.05-7.08 (m, 1H, N-C_q-CH-CH-C*H*), 6.97-6.99 (d, 2H, *J* = 7.4 Hz, N-C_q-C*H* × 2), 5.01 (br s, 2H, NH_2), 2.40 (s, 3H, CH_3).

$^{13}C\{^{1}H\}$ NMR (125 MHz, $CDCl_3$): δ/ppm: 155.2 (C_q), 148.2 (C_q), 140.6 (C_q), 131.8 (C_q), 129.0 (2 × *C*-H), 128.8 (*C*-H), 126.4 (2 × *C*-H), 122.8 (2 × *C*-H), 121.4 (2 × *C*-H), 20.9 (CH_3).

IR (neat): ν_{max}/cm^{-1}: 3441 (N-H stretch), 3292 (N-H stretch), 3067 (C-H stretch), 3051 (C-H stretch),

2915 (C-H alkyl stretch), 1627 (C=N stretch), 1587 (C=C stretch), 1563 (C=C stretch), 1578 (C=C stretch).

HRMS (ESI): Calc. for $C_{14}H_{15}N_2^+$. Theoretical: 211.1230 Observed: 211.1224.

(*Z*)-3-cyano-*N*'-phenylbenzimidamide (**29**)

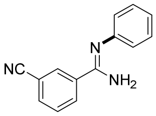

Chan-Lam procedure B (0.40 mmol scale boronic acid). Purification was carried out using a gradient 0% to 50% EtOAc in petroleum ether (40-60) with 1% Et_3N modifier, affording a white powder (10 mg, 11%).

mp 105-109 °C.

^{1}H NMR (500 MHz, $CDCl_3$): δ/ppm: 8.19 (br s, 1H, N≡C-C_q-C*H*-C_q), 8.10 (d, 1H, *J* = 7.6 Hz, N=C_q-C_q-C*H*-CH), 7.76 (m, 1H, N≡C-C_q-C*H*-CH) 7.55-7.59 (m, 1H, NC-C_q-CH-C*H*), 7.35-7.39 (m, 2H, N-C_q-CH-C*H* × 2), 7.09-7.12 (m, 1H, N-C_q-CH-CH-C*H*), 6.98 (d, 2H, *J* = 7.4 Hz, , N-C_q-C*H* × 2), 4.98 (br s, 2H, N*H*$_2$).

^{13}C{^{1}H} NMR (125 MHz, $CDCl_3$): δ/ppm: 152.6 (C_q), 148.0 (C_q), 136.2 (C_q), 133.5 (*C*-H), 130.7 (*C*-H), 130.3 (*C*-H), 129.2 (2 × *C*-H), 129.0 (*C*-H), 123.4 (2 × *C*-H), 121.0 (*C*-H), 117.7(*C*≡N), 112.5 (C_q).

IR (neat): **ν_{max}/cm^{-1}**: 3426 (N-H stretch), 3253 (N-H stretch), 3051 (C-H stretch), 3028 (C-H stretch), 2227 (C≡N stretch), 1640 (C=N stretch), 1619 (C=N stretch), 1589 (C=C stretch), 1578 (C=C stretch).

HRMS (ESI): Calc. for $C_{14}H_{12}N_3^+$. Theoretical: 222.1026 Observed: 222.1020.

(*Z*)-4-((5-(4-carbamimidoylphenoxy)pentyl)oxy)-*N*'-phenylbenzimidamide (**30**)

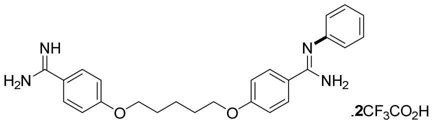

Using Chan-Lam procedure C (0.20 mmol scale boronic acid). Purification was carried out by semi-preparative RP-HPLC using method B, affording a white amorphous solid (31 mg, 24%).

^{1}H NMR (500 MHz, $CDCl_3$): δ/ppm: 11.21 (br s, 1H, CF_3-CO_2*H*), 9.65 (br s, 1H, CF_3-CO_2*H*), 9.13 (br s, 2H, N*H*$_2$), 8.84 (br s, 3H, 3 × N*H*), 7.89 (d, 2H, *J* = 8.6 Hz, C_q-N=C_q-C_q-C*H* × 2) 7.82 (d, 2H, *J* = 8.8 Hz, HN=C_q-C_q-C*H* × 2) 7.56-7.60 (m, 2H, N-C_q-C*H*× 2), 7.45-7.49 (m, 3H, N-C_q-CH-C*H*× 2-C*H*) 7.21 (d, 2H, *J* = 8.6 Hz, O-C_q-C*H* × 2), 7.17 (d, 2H, *J* = 8.8 Hz, O-C_q-C*H* × 2), 4.12-4.17 (m, 4H, O-C*H*$_2$ × 2), 1.81-1.88 (m, 4H, O-CH_2-C*H*$_2$ × 2), 1.57-1.65 (m, 2H, O-CH_2-CH_2-C*H*$_2$).

^{13}C{^{1}H} NMR (125 MHz, $CDCl_3$): δ/ppm: 164.7 (C_q), 163.1 (C_q), 163.0 (C_q), 162.3 (C_q), 134.8 (C_q), 130.8 (2 × *C*-H), 130.1 (2 × *C*-H), 129.9 (2 × *C*-H), 128.2 (*C*-H), 125.4 (2 × *C*-H), 120.0 (C_q), 119.4 (C_q), 114.7 (4 × *C*-H), 68.1 (*C*H$_2$), 68.0 (*C*H$_2$), 28.2 (2 × *C*H$_2$), 22.0 (*C*H$_2$).

^{19}F{^{1}H} NMR (470 MHz, $CDCl_3$): δ/ppm: -73.5.

IR (neat): **ν_{max}/cm^{-1}**:3326 (N-H Stretch), 3105 (CO_2H stretch), 2947 (CO_2H stretch), 1667 (C=O stretch), 1610 (C=N stretch), 1495 (C=C stretch).

HRMS (ESI): Calc. for $C_{25}H_{29}N_4O_2^+$. Theoretical: 417.2285 Observed: 417.2272.

(*Z*)-*N*'-([1,1'-biphenyl]-4-yl)-4-((5-(4-carbamimidoylphenoxy)pentyl)oxy)benzimidamide (**31**)

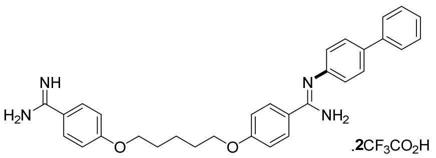

Using Chan-Lam procedure C (0.20 mmol scale boronic acid). Purification was carried out by semi-preparative RP-HPLC using method B, affording a white amorphous solid (29 mg, 20%).

^{1}H NMR (500 MHz, $CDCl_3$): δ/ppm: 11.27 (br s, 1H, CF_3-CO_2*H*), 9.70 (br s, 1H, CF_3-CO_2*H*), 9.13 (s, 2H, N*H*$_2$) 8.95 (br s, 1H, N*H*), 8.86 (s, 2H, N*H*$_2$) 7.87-7.92 (m, 4H, 4 × C*H*) 7.82 (d, 2H, *J* = 9.0 Hz, HN=C_q-C_q-C*H* × 2), 7.74 (d, 2H, *J* = 7.4 Hz, C_q-C_q-C*H* × 2), 7.51-7.56 (m, 4H, 4 × C*H*), 7.42-7.44 (m, 1H, C_q-C_q-CH-CH-C*H*), 7.23 (d, 2H, *J* = 8.7 Hz, O-C_q-C*H* × 2), 7.18 (d, 2H, *J* = 9.0 Hz, O-C_q-C*H* × 2), 4.13-4.18 (m, 4H, O-C*H*$_2$ × 2), 1.82-1.89 (m, 4H, O-CH_2-C*H*$_2$ × 2), 1.59-1.65 (m, 2H, O-CH_2-CH_2-C*H*$_2$).

^{13}C{^{1}H} NMR (125 MHz, $CDCl_3$): δ/ppm: 164.7 (C_q), 163.1 (C_q), 163.0 (C_q), 162.4 (C_q), 139.8 (C_q), 139.0 (C_q), 134.2 (C_q), 130.9 (2 × *C*-H), 130.2 (2 × *C*-H), 129.1 (2 × *C*-H), 128.1 (2 × *C*-H), 127.9 (*C*-H), 126.7 (2 × *C*-H), 125.9 (2 × *C*-H), 120.0 (C_q), 119.4 (C_q), 114.8 (4 × *C*-H), 68.1 (*C*H_2), 68.0 (*C*H_2), 28.2 (2 × *C*H_2), 22.1 (*C*H_2).

^{19}F{^{1}H} NMR (470 MHz, $CDCl_3$): δ/ppm: -73.5.

IR (neat): **ν_{max}/cm^{-1}**: 3320 (N-H stretch), 3125 (CO_2H stretch), 2945 (CO_2H stretch), 1671 (C=O stretch), 1610 (C=N stretch), 1493 (C=C stretch).

HRMS (ESI): Calc. for $C_{31}H_{33}N_4O_2^+$. Theoretical: 493.2598 Observed: 493.2590.

(*Z*)-4-((5-(4-carbamimidoylphenoxy)pentyl)oxy)-*N*'-(4-(trifluoromethyl)phenyl)benzimidamide (**32**)

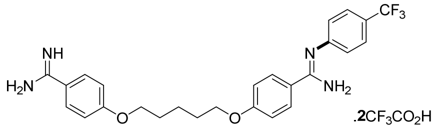

Using Chan-Lam procedure C (0.20 mmol scale boronic acid). Purification was carried out by semi-preparative RP-HPLC using method B, affording a white amorphous solid (30 mg, 21%).

^{1}H NMR (500 MHz DMSO-*d6*): δ/ppm: 11.43 (br s, 1H, CF_3-CO_2*H*), 9.82 (br s, 1H, CF_3-CO_2*H*), 9.12 (br s, 3H, 3 × N*H*), 8.81 (br s, 2H, N*H*$_2$), 7.94 (d, 2H, *J* = 8.5 Hz, F_3C-C_q-CH-C*H* × 2), 7.89 (d, 2H, *J* = 8.7 Hz, C_q-N=C_q-C_q-C*H* × 2), 7.81 (d, 2H, *J* = 8.9 Hz, HN=C_q-C_q-C*H* × 2), 7.67 (d, 2H, *J* = 7.8 Hz, F_3C-C_q-C*H* × 2), 7.22 (d, 2H, *J* = 8.9 Hz, O-C_q-C*H* × 2), 7.16 (d, 2H, *J* = 8.7 Hz, O-C_q-C*H* × 2), 4.12-4.16 (m, 4H, O-C*H*$_2$ × 2), 1.81-1.87 (m, 4H, O-CH_2-C*H*$_2$ × 2), 1.57-1.63 (m, 2H, O-CH_2-CH_2-C*H*$_2$).

^{13}C{^{1}H} NMR (125 MHz, DMSO-*d6*): δ/ppm: 164.6 (C_q), 163.3 (C_q), 163.0 (C_q), 162.7 (C_q), 139.0 (C_q), 131.1 (2 × C*H*), 130.2 (2 × C*H*), 128.0 (q , *J* = 31.2 Hz, C_q) 127.1 (q, *J* = 3.5 Hz, 2 × C*H*), 126.1 (2 × C*H*), 123.9 (q, *J* = 272.1 Hz, *C*F_3), 119.9 (C_q), 119.4 (C_q), 114.8 (4 × C*H*), 68.0 (C*H*$_2$), 68.1 (C*H*$_2$), 28.2 (2 × C*H*$_2$), 22.0 (C*H*$_2$).

^{19}F{^{1}H} NMR (470 MHz, DMSO-*d6*)**:** δ/ppm: -60.9, -73.6.

IR (neat): **ν_{max}/cm^{-1}**: 3328 (N-H stretch), 3105 (CO_2H stretch), 2947 (CO_2H stretch), 2874 (CO_2H stretch), 1667 (C=O stretch), 1608 (C=N stretch), 1495 (C=C stretch).

HRMS (ESI): $C_{26}H_{28}F_3N_4O_2^+$ Theoretical: 485.2159 Observed: 485.2149.

(*Z*)-4-((5-(4-carbamimidoylphenoxy)pentyl)oxy)-N'-(4-(dimethylamino)phenyl)benzimidamide (**33**)

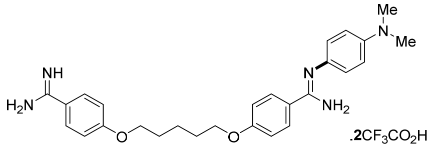

Using Chan-Lam procedure C (0.20 mmol scale boronic acid). Purification was carried out by semi-preparative RP-HPLC using method B, affording a pale green amorphous solid (27 mg, 20%).

^{1}H NMR (500 MHz DMSO-*d6*): δ/ppm: 10.90 (br s, 1H, CF_3-CO_2*H*), 9.45 (br s, 1H, CF_3-CO_2*H*), 9.12 (br s, 2H, NH_2), 8.78 (br s, 2H, NH_2), 8.49 (br s, 1H, N*H*), 7.86 (d, 2H, *J* = 8.9 Hz, C_q-N=C_q-C_q-C*H* × 2), 7.81 (d, 2H, *J* = 8.9 Hz, HN=C_q-C_q-C*H* × 2), 7.23 (d, 2H, *J* = 9.0 Hz, N-C_q-C*H* × 2), 7.16-7.24 (m, 4H, O-C_q-C*H* × 4), 6.85 (d, 2H, *J* = 8.7 Hz, $(H_3C)_2$N-C_q-C*H* × 2), 4.12-4.16 (m, 4H, O-CH_2 × 2), 2.97 (s, 6H, N(CH_3)$_2$), 1.81-1.87 (m, 4H, O-CH_2-CH_2 × 2), 1.59-1.64 (m, 2H, O-CH_2-CH_2-CH_2).

^{13}C{^{1}H} NMR (125 MHz, DMSO-*d6*): δ/ppm: 164.5 (C_q), 163.1 (C_q), 162.9 (C_q), 162.3 (C_q), 150.0 (C_q), 130.5 (2 × C*H*), 130.2 (2 × C*H*), 126.6 (2 × C*H*),), 122.7 (C_q), 120.1(C_q) 119.4 (C_q), 114.8 (2 × C*H*) 114.7 (2 × C*H*), 112.8 (2 × C*H*), 68.0 (CH_2), 68.1 (CH_2), 39.70 (N(*C*H_3)$_2$, Observed from a HSQC experiment), 28.2 (2 × CH_2), 22.1 (CH_2).

^{19}F{^{1}H} NMR (470 MHz, DMSO-*d6*): δ/ppm: -73.7.

IR (neat): **ν_{max}/cm^{-1}**: 3327 (N-H stretch), 3105 (CO_2H stretch), 2947 (CO_2H stretch), 2874 (CO_2H stretch), 1647 (C=O stretch), 1608 (C=N stretch), 1592 (C=C stretch), 1490 (C=C stretch).

HRMS (ESI): Calc. for $C_{27}H_{34}N_5O_2^+$. Theoretical: 460.2707 Observed: 460.2712.

(*Z*)-4-((5-(4-carbamimidoylphenoxy)pentyl)oxy)-*N*'-(3-fluoro-4-methoxyphenyl)benzimidamide (**34**)

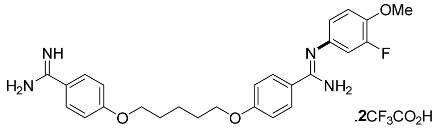

Using Chan-Lam procedure C (0.20 mmol scale boronic acid). Purification was carried out by semi-preparative RP-HPLC using method B, affording a white amorphous solid (28 mg, 20%).

^{1}H NMR (500 MHz, DMSO-*d6*): δ/ppm: 11.14, (br s, 1H, CF_3-CO_2*H*), 9.62 (br s, 1H, CF_3-CO_2*H*) 9.13 (br s, 2H, NH_2), 8.90 (br s, 2H, NH_2), 8.75 (br s, 1H, N*H*), 7.87 (d, 2H, *J* = 8.7 Hz, C_q-N=C_q-C_q-C*H* × 2), 7.82 (d, 2H, *J* = 8.9 Hz, HN=C_q-C_q-C*H* × 2), 7.45 (d, 1H, *J* = 10.7 Hz, N-C_q-C*H*-CF), 7.33-7.38 (m, 1H, N-C_q-C*H*-CH), 7.16-7.26 (m, 5H, 5 × C*H*), 4.12-4.16 (m, 4H, O-CH_2 × 2), 3.91 (s, 3H, CH_3), 1.83-1.86 (m, 4H, O-CH_2-CH_2 × 2), 1.57-1.65 (m, 2H, O-CH_2-CH_2-CH_2).

^{13}C{^{1}H} NMR (125 MHz, DMSO-*d6*): δ/ppm: 165.2 (C_q), 163.6 (C_q), 163.5 (C_q), 163.3 (C_q), 151.9 (d , *J* = 248.9 Hz, *C*-F), 147.6 (C_q), 131.2 (2 × *C*-H), 130.6 (2 × *C*-H), 127.6 (C_q), 123.2 (*C*-H), 120.4 (C_q), 119.9 (C_q), 115.3 (2 × *C*-H), 115.2 (2 × *C*-H), 115.0 (*C*-H) 114.8 (d, *J* = 20.0 Hz, *C*-H), 68.6 (CH_2), 68.5 (CH_2), 56.8 (CH_3), 28.7 (CH_2), 22.6 (CH_2).

^{19}F{^{1}H} NMR (470 MHz, DMSO-*d6*): δ/ppm: -73.7, -132.5.

IR (neat): **ν_{max}/cm^{-1}**: 3330 (N-H stretch), 3095 (CO_2H stretch), 2943 (CO_2H stretch), 2874 (CO_2H stretch), 1669 (C=O stretch), 1610 (C=N stretch), 1521 (C=C stretch), 1495 (C=C stretch).

HRMS (ESI): Calc. for $C_{26}H_{30}FN_4O_3^+$. Theoretical: 465.2296 Observed: 465.2289.

(*Z*)-4-((5-(4-carbamimidoylphenoxy)pentyl)oxy)-*N*'-(quinolin-3-yl)benzimidamide (**35**)

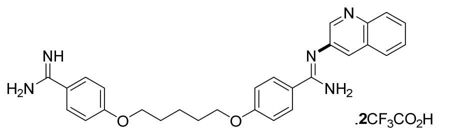

Using Chan-Lam procedure C (0.20 mmol scale boronic acid). Purification was carried out by semi-preparative RP-HPLC using method B, affording a white amorphous solid (21 mg, 15%).

^{1}H NMR (500 MHz, $CDCl_3$): δ/ppm: 11.6 (br s, 1H, CF_3-CO_2*H*), 9.83 (br s, 1H, CF_3-CO_2*H*), 9.14 (br s, 3H, 3 × N*H*), 8.98 (br s, 1H, N-C_q-C*H*-N), 8.93 (br s, 2H, NH_2), 8.52 (br s, 1H, N-C_q-C*H*-C_q), 8.09-8.14 (m, 2H, N-C_q-CH-C_q-C*H*-C*H*), 7.97 (d, 2H, *J* = 8.5 Hz, C_q-N=C_q-C_q-C*H* × 2), 7.86-7.90 (m, 1H, N-C_q-CH-N-C_q-C*H*), 7.83 (d, 2H, *J* = 8.9 Hz, HN=C_q-C_q-C*H* × 2), 7.71-7.74 (m, 1H, N-C_q-CH-N-C_q-CH-C*H*), 7.25 (d, 2H, *J* = 8.5 Hz, O-C_q-C*H* × 2), 7.17 (d, 2H, *J* = 8.9 Hz, O-C_q-C*H* × 2), 4.13-4.19 (m, 4H, O-CH_2 × 2), 1.82-1.90 (m, 4H, O-CH_2-CH_2 × 2), 1.58-1.66 (m, 2H, O-CH_2-CH_2-CH_2).

^{13}C{^{1}H} NMR (125 MHz, $CDCl_3$): δ/ppm: 164.6 (C_q), 163.4 (C_q), 163.3 (C_q), 163.0 (C_q), 158.1 (C_q), 157.8 (C_q), 148.2 (*C*-H), 146.6 (C_q), 132.5 (*C*-H), 130.9 (2 × *C*-H), 130.4 (*C*-H), 130.2 (2 × *C*-H), 128.8 (*C*-H), 128.4 (*C*-H), 127.6 (*C*-H), 119.8 (C_q), 119.4 (C_q), 114.8 (2 × *C*-H), 114.7 (2 × *C*-H), 68.1 (*C*H_2), 68.0 (*C*H_2), 28.2 (2 × *C*H_2), 22.1 (*C*H_2).

^{19}F{^{1}H} NMR (470 MHz, $CDCl_3$): δ/ppm: -73.7.

IR (neat): **ν_{max}/cm^{-1}**: 3326 (N-H stretch), 3093 (CO_2H stretch), 2943 (CO_2H stretch), 1667 (C=O stretch), 1608 (C=N stretch), 1493 (C=C stretch).

(Z)-4-((5-(4-carbamimidoylphenoxy)pentyl)oxy)-N'-(3-((5-(dimethylamino)naphthalene)-1-sulfonamido)phenyl)benzimidamide (**36**)

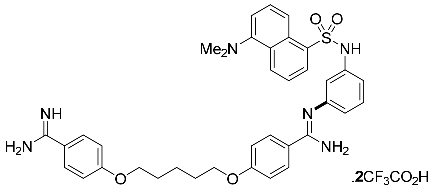

Using Chan-Lam procedure C (0.20 mmol scale boronic acid). Purification was carried out by semi-preparative RP-HPLC using method B, affording a white amorphous solid (6 mg, 5%).

^{1}H NMR (500 MHz, DMSO-*d6*)**:** δ 11.12 (br s, 1H, CF_3-CO_2*H*), 11.05 (br s, 1H, N*H*) 9.63 (br s, 1H, CF_3-CO_2*H*), 9.13 (s, 2H, NH_2), 8.90 (s, 2H, NH_2), 8.82 (br s, 1H, N*H*), 8.84 (d, 1H, *J* = 8.4 Hz, HN-SO_2-C_q-C_q-C_q-C*H*), 8.38 (d, 1H, *J* = 8.7 Hz, HN-SO_2-C_q-C_q-C*H*), 8.28 (d, 1H, *J* = 7.2 Hz, HN-SO_2-C_q-C*H*), 7.82 (d, 4H, *J* = 8.9 Hz, 4 × C*H*) 7.62-7.66 (m, 2H, 2 × C*H*), 7.33-7.37 (m, 1H, C=N-C_q-CH-C*H*), 7.27 (d, 1H, $(CH_3)_2$N-C_q-C*H*), 7.15-7.18 (m, 4H, 4 × C*H*), 7.09-7.11 (m, 2H, 2 × C*H*), 7.02 (d, 1H, *J* = 6.2 Hz, C=N-C_q-C*H*-CH), 4.12-4.15 (m, 4H, O-CH_2 × 2), 2.82 (s, 6H, N(CH_3)$_2$), 1.80-1.86 (m, 4H, O-CH_2-CH_2 × 2), 1.57-1.62 (m, 2H, O-CH_2-CH_2-CH_2).

^{13}C{^{1}H} NMR (125 MHz, DMSO-*d6*)**:** δ 164.6 (C_q), 163.1 (C_q), 163.0 (C_q), 158.2 (C_q), 157.9 (C_q), 151.5 (C_q), 139.3 (C_q) 135.6 (C_q), 134.7 (C_q), 130.9 (2 × *C*-H), 130.7 (C_q), 130.3 (*C*-H), 130.2 (2 × *C*-H), 129.7 (*C*-H), 129.0 (*C*-H), 128.9 (C_q), 123.5 (*C*-H), 119.8 (C_q), 119.6 (*C*-H), 118.5 (*C*-H), 117.2 (*C*-H), 115.3 (*C*-H), 114.7 (2 × *C*-H), 114.6 (2 × *C*-H), 68.1 (*C*H_2), 68.0 (*C*H_2), 45.0 (2 × *C*H_3) 28.2 (2 × *C*H_2), 22.0 (*C*H_2).

$^{19}F\{^1H\}$ NMR (470 MHz, DMSO-*d6*)**:** δ -73.9.

ν_{max} (neat): 3100 (CO_2H stretch), 3087 (CO_2H stretch), 1657 (C=O stretch), 1608 (C=N stretch), 1485 (C=C stretch).

HRMS (ESI): Calc. for $C_{37}H_{41}N_6O_4S^+$. Theoretical: 665.2905 Observed: 665.2896.

Complex **4**

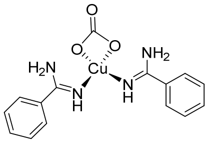

To an oven dried 100 mL round-bottomed flask was added was added benzamidine **1** (1.00 g, 8.32 mmol), $Cu(OAc)_2$ (785 mg, 4.32 mmol) and K_2CO_3 (1.15 g, 8.32 mmol). Isopropanol (18.1 mL) was then added to the solid mixture, sealed under air, and stirred at room temperature for 2 h. The resulting purple suspension was filtered, and the purple precipitate was dried under high vacuum to yield a purple powder-like solid (2.01 g). This purple powder was then recrystallised from MeCN and subjected to a hot filtration to afford deep purple crystals (80 mg, 5%). Crystallographic data in tables S8 – S13.

Trypanosoma experiments

Resazurin-based drug sensitivity assay

The synthesised pentamidine analogues were tested for anti-kinetoplastid activity using the resazurin ('alamar blue') assay as described previously.[2] The bloodstream forms (BSF) of *Trypanosoma brucei brucei* s427WT and the multidrug resistant strain B48 were cultured and maintained in HMI-9 medium supplemented with 10% heat inactivated Fetal Bovine Serum (FBS), 14 µL/L β-mercaptoethanol, and 3.0 g/L $NaHCO_3$ adjusted to pH 7.4, and incubated at 37 ºC in a 5% CO_2 atmosphere. BSF *T. congolense Tc*-IL3000 WT and diminazene-resistant *T. congolense* strain 6C3 were cultured as described previously [3,4], at 34 ºC/5% CO_2 in basal medium prepared with MEM medium (Sigma-Aldrich), 26 mM $NaHCO_3$, 25 mM HEPES, 5.6 mM D-glucose, 1 mM sodium pyruvate, 100 µM hypoxanthine, 40 µM adenosine, 16.5 µM thymidine, and 25 µM bathocuproine disulfonic acid disodium salt, supplemented with 1.6 mM glutamine, 100 units/mL penicillin, 0.1 mg/mL streptomycin, β-mercaptoethanol (0.0014% v/v), 15% goat serum (Gibco), and 5% Serum Plus II (Sigma Aldrich). The promastigote stage of *Leishmania mexicana* WT (strain MNY/BZ/62/M379) used in this study was cultured at 27 ºC in custom-made HOMEM medium (Gibco) supplemented with 10% FBS as described.[5]

Briefly, 200 µL of 40 µM of each test compound in the appropriate medium was placed in the first well of a row of a 96-well plate (Greiner Bio-one GmbH, Frickenhausen, Germany). 100 µL of fresh media was added to the 11 other wells in the row and a double serial dilution was performed while leaving the 12th well as drug-free. Pentamidine and diminazene were used as positive control.

The cells were then adjusted to a density of 2 × 10^5, 5 × 10^5 or 2 × 10^6 cells/ml in the appropriate medium for *T. brucei*, *T. congolense* and *L. mexicana* respectively and of these cell suspensions 100 µL was added to the appropriate wells. This was followed by incubation for 48 h for the *Trypanosoma* spp or 72 h for *Leishmania*, after which 20 µL of 125 µg/mL resazurin sodium salt (Sigma) in PBS was added. The assay is based on the ability of live cells to reduce blue, non-fluorescent resazurin to pink, fluorescent resorufin - a reaction that does not occur with dead cells - producing a fluorescent signal proportionate to cell numbers (Gould et al., 2008). The plates were further incubated for 24 h for the trypanosomes or 48 h for *Leishmania* under the same conditions. Fluorescence was then determined using a FLUOstar Optima (BMG Labtech, Durham, NC, USA) at λ_{exc} = 544 nM and λ_{em} = 590 nm, and a half maximal effective concentration (EC_{50}) was calculated for each compound by non-linear regression using an equation for a sigmoidal dose-response curve with variable slope (GraphPad Prism 5.0, GraphPad Software Inc., San Diego).

Real-time measurement of fluorescence in parasites

The uptake of compound **36** was measured in real time using a fluorimeter, essentially as described previously but with some modifications.[6] Cultures of *T. b. brucei, T. congolense* and *L. mexicana* were harvested at mid-log growth phase by centrifugation for 10 min at 2400 rpm. The cells were washed twice in assay buffer and resuspended at a density of 10^8 cells/mL; 100 µL/well of each cell suspension was transferred to wells of a white opaque 96-well culture plate. Different dilutions of **36** were prepared at twice the desired final concentration in assay buffer with or without pentamidine, and 100 µL of the drug preparation was then transferred to designated wells containing cells of the plate. The plate was inserted into a microplate reader (FLUOstar Optima, BMG Labtech) set at 37 ºC and 5% CO_2, and fluorescence was measured over time using 355/520 excitation and emission filters.

Localisation of cellular targets using fluorescence microscopy

Fluorescence microscopy was carried out to locate the target of **36** using DNA and mitochondrial markers as described previously (Cueto-Díaz et al., 2021). Briefly, about 2 × 10^6 *T. b. brucei* cells were incubated with 10 µM **36** in complete HMI-9 for 30 min, followed by the addition of MitoTracker Green FM (Invitrogen, M7514) to a final concentration of 100 nM and a by further incubation for 10 min (at 37ºC and 5% CO_2). The cells were then centrifuged at 2400 rpm for 10 min, and the pellet was washed once and resuspended in sterile 1× PBS, pH 7.4. Hoechst 33342 Staining Dye Solution (Abcam, ab228551) was then added to the sample at 5 µg/mL followed by incubation in the dark at room temperature for 10 min. The cells were washed again in ice-cold, sterile 1× PBS by centrifugation and the pellet re-suspended in 50 µL of ice-chilled PBS-primed CyGEL™ (Abcam, ab109204) for immobilisation. The sample was then transferred into a clean glass slide on ice, covered with coverslip and sealed with nail varnish. Images were acquired immediately, using a DeltaVision microscope (GE Healthcare) and softWoRx software, and processed with the ImageJ software package.

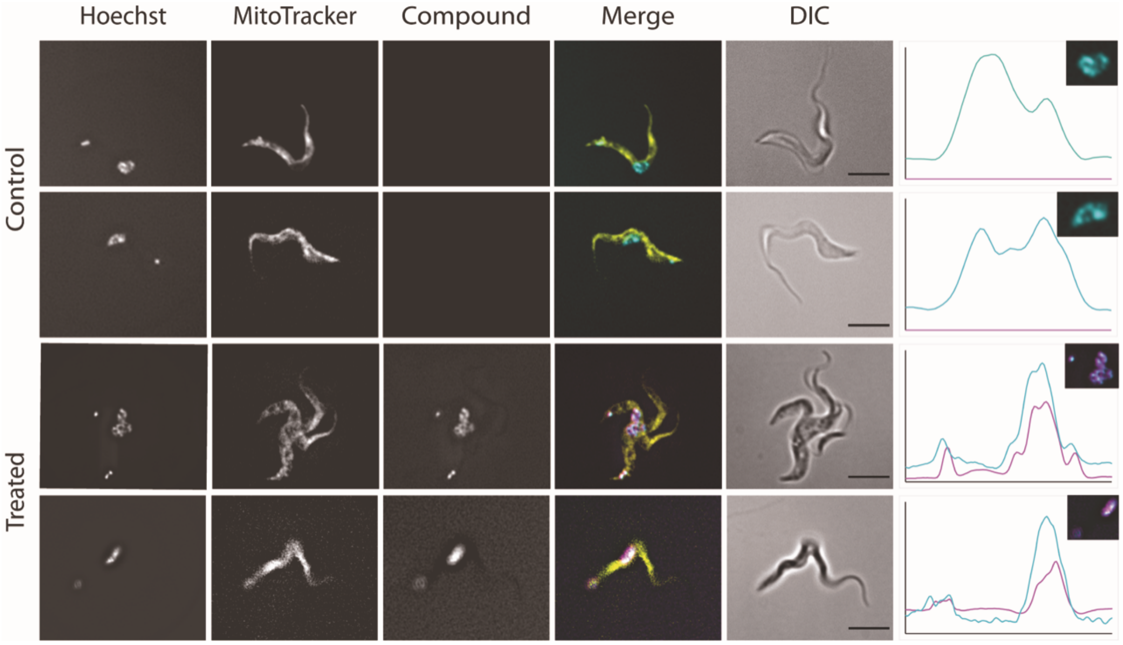

Figure S4. Fluorescence microscopy images of trypanosoma using pentamidine analogue **36**.

DNA UV Melt Analysis

DNA UV melt analyses were performed on a Shimadzu 1800 UV/Vis spectrometer equipped with a pelter temperature controller using a 1 cm pathlength cell. ON1/ON1c (5′ GGA AAT TTG C 3′)/(5′ GCA AAT TTC C 3′) and ON2/ON2c (5′ GGA TAT ATG C 3′)/(5′ GCA TAT ATC C 3′) were purchased from Eurogentec. ON pairs were analysed using a buffer containing 10 mM monobasic phosphate, 10 mM dibasic phosphate and 100 mM NaCl corrected to pH 7.0. Pentamidine analogues were dissolved in DMSO and added to give a DMSO content <0.1%. The final duplex ON concentration was 2 μM and each DNA ligand was added to an 8 μM (4 eq.) concentration. Denaturing profiles were recorded from 20 °C to 90 °C at a rate of 1 °C/min with three concordant melt profiles produced. Each ON pair was independently repeated 3 times. The reported melting temperatures were produced from a gaussian fit of the 1st derivative of the melt profile and averaged across the technical and biological repeats (n=9). Significance values were calculated using Student unpaired two-tailed t-test (**** P ≤ 0.0001).

Table S7. Calculated melt temperatures (T_m) and change in melt temperatures (ΔT_m).

	T_m (°C)				
	**Free ON**	**PMD**	**33**	**36**	**DA**
ON1/ON1c	38.29 ± 0.21	44.23 ± 0.16	45.19 ± 0.20	42.86 ± 0.45	51.16 ± 0.12
ON2/ON2c	32.49 ± 0.11	37.36 ± 0.12	37.97 ± 0.10	35.63 ± 0.61	44.22 ± 0.15
	ΔT_m (°C)				
ON1/ON1c		5.94 ± 0.26	6.89 ± 0.29	4.56 ± 0.50	12.90 ± 0.24
ON2/ON2c		4.86 ± 0.16	5.48 ± 0.15	3.14 ± 0.62	11.70 ± 0.19

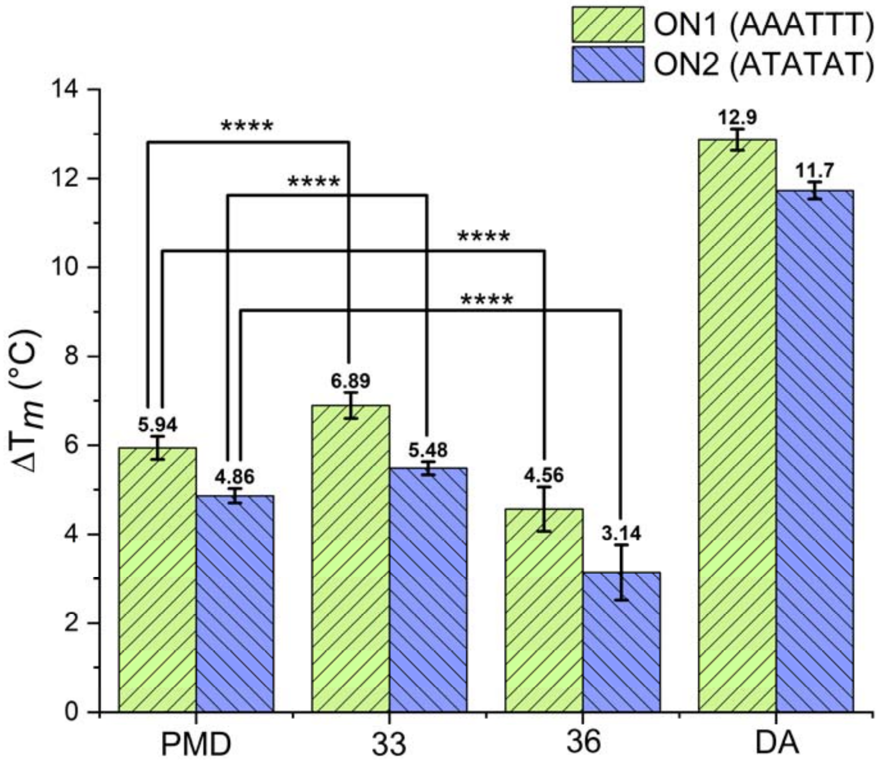

Figure S4. Comparison of the DNA duplex stabilisation (ΔT_m) for a range of minor groove binding ligands. PMD, pentamidine; DA, diminazene aceturate, **33** and **36**. Significance values were calculated using Student unpaired two-tailed t-test (**** $P \leq 0.0001$).

NMR spectra

(Z)-*N*'-phenylbenzimidamide (**2**) - ^{1}H NMR

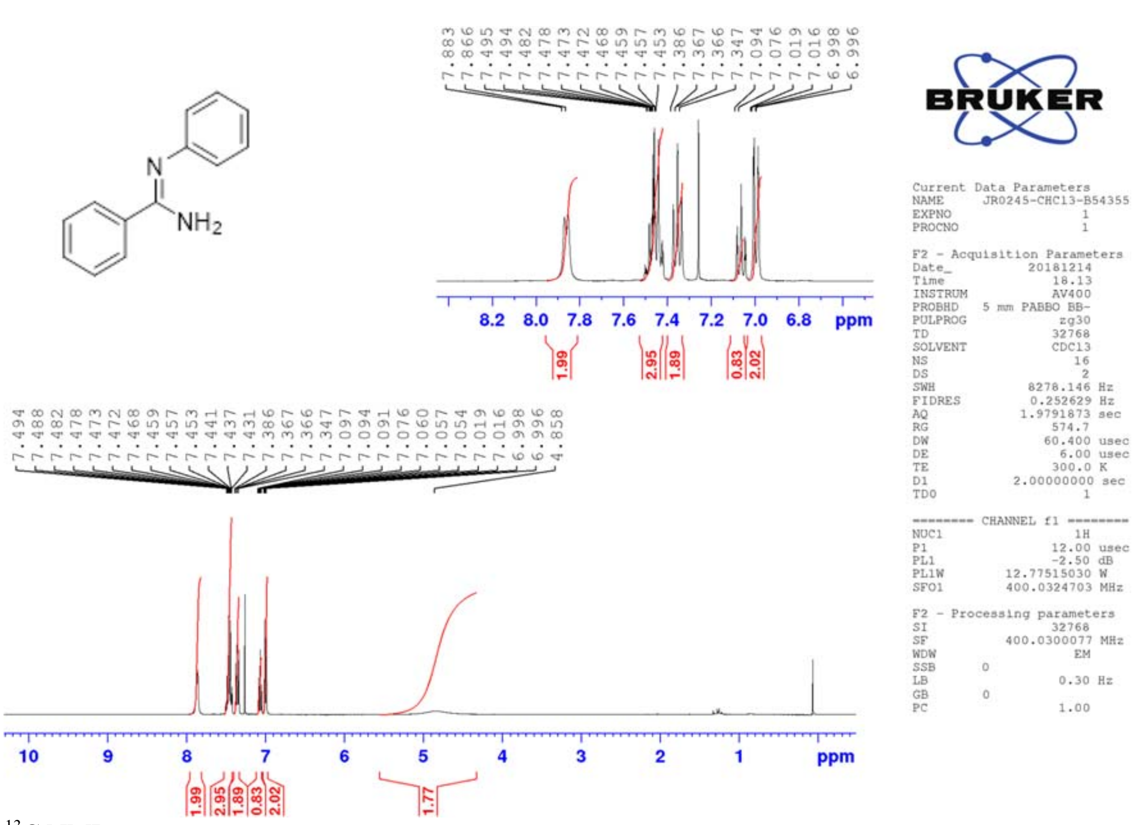

^{13}C NMR

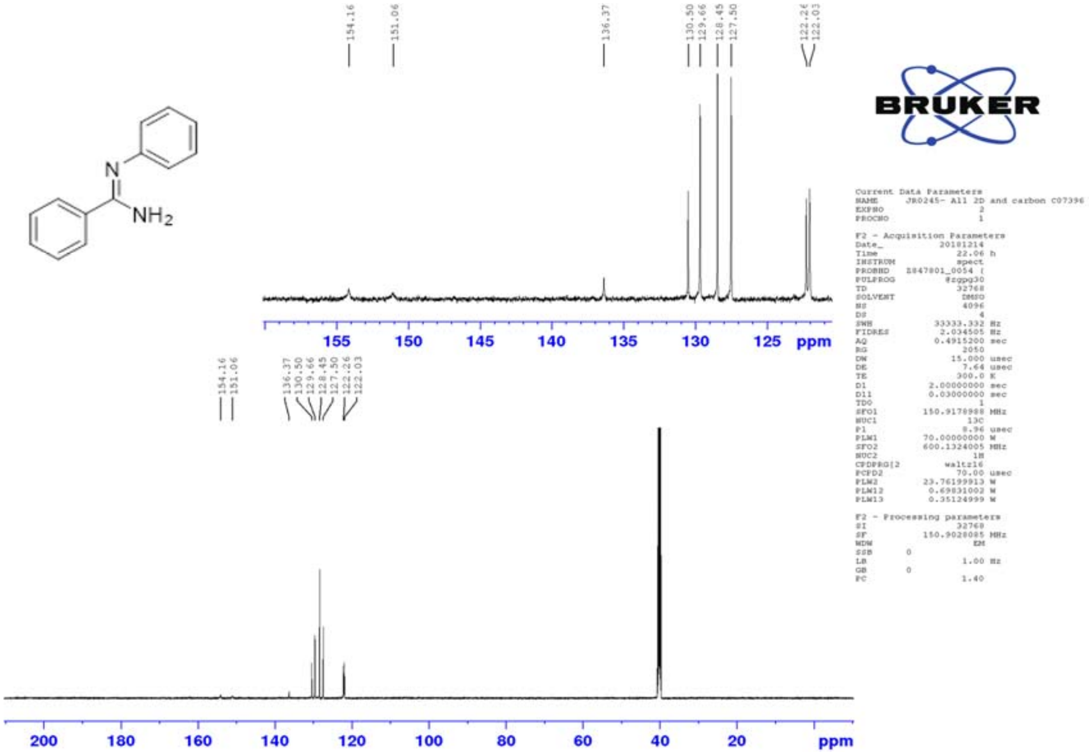

(*Z*)-*N*,*N'*-diphenylbenzimidamide (**3**) - ^{1}H NMR

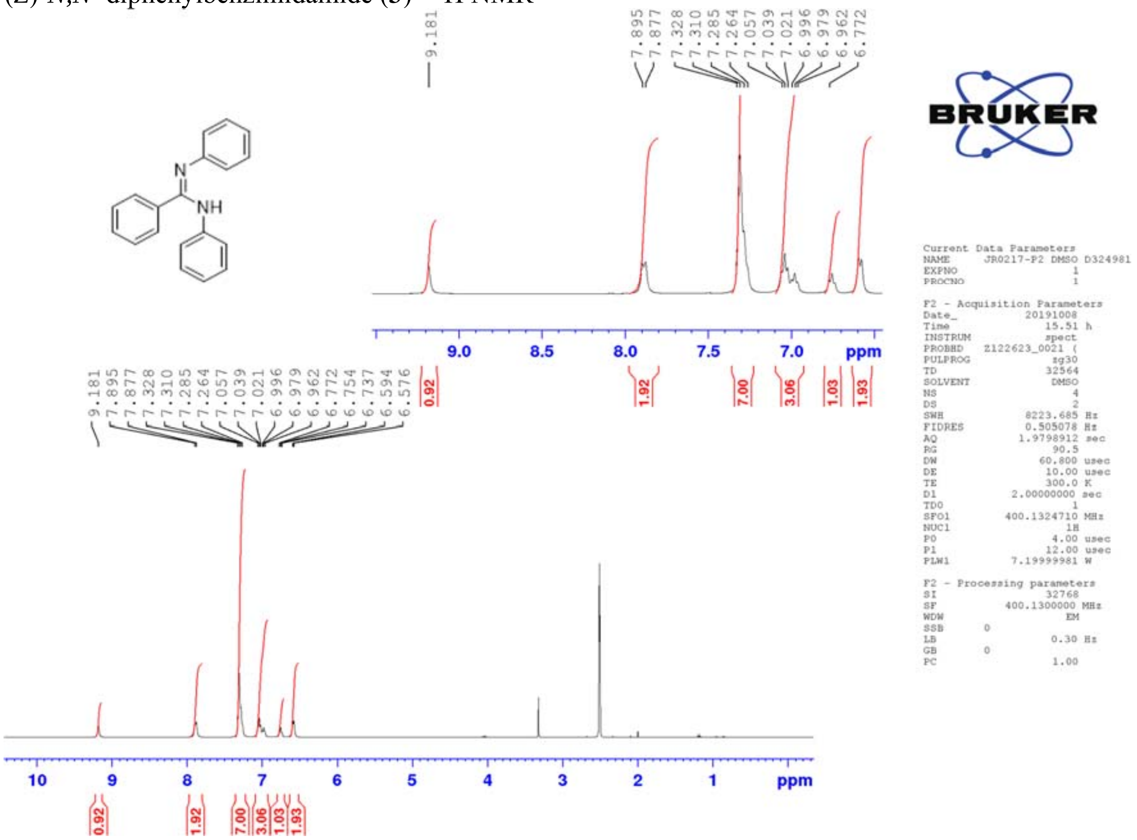

^{13}C NMR

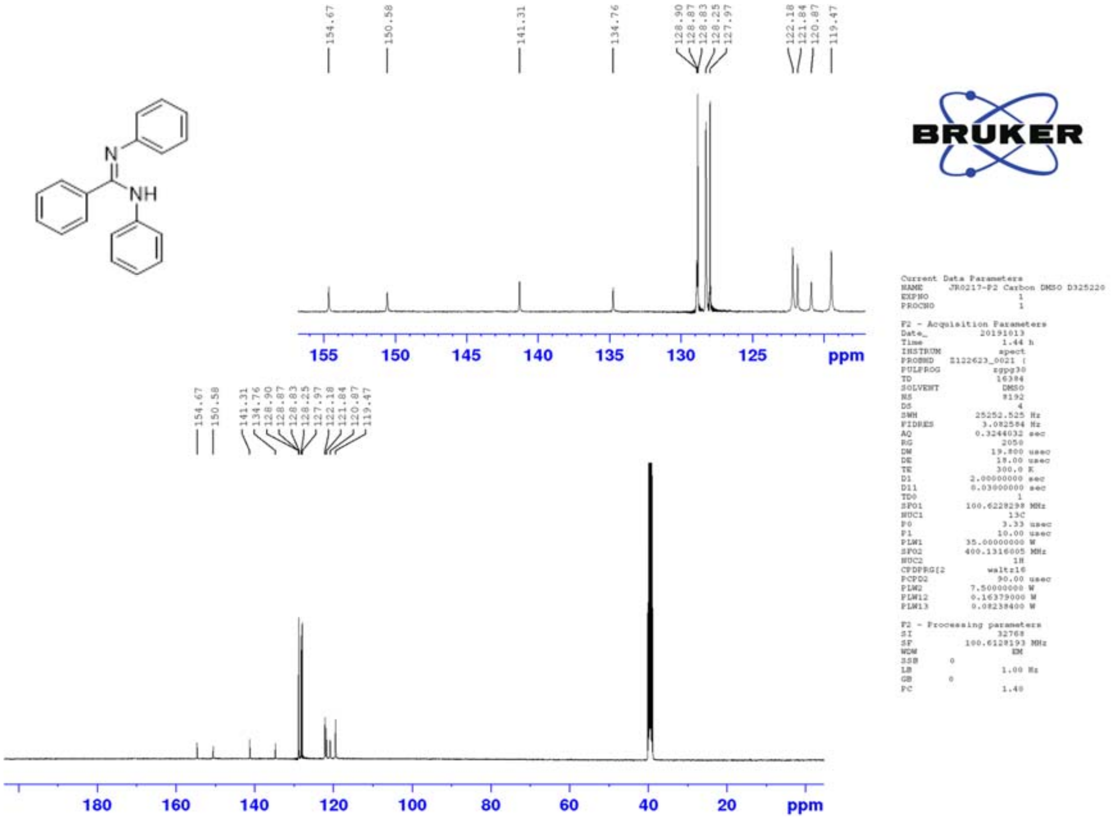

(Z)-*N'*-(4-fluorophenyl)benzimidamide (**7**) - ^{1}H NMR

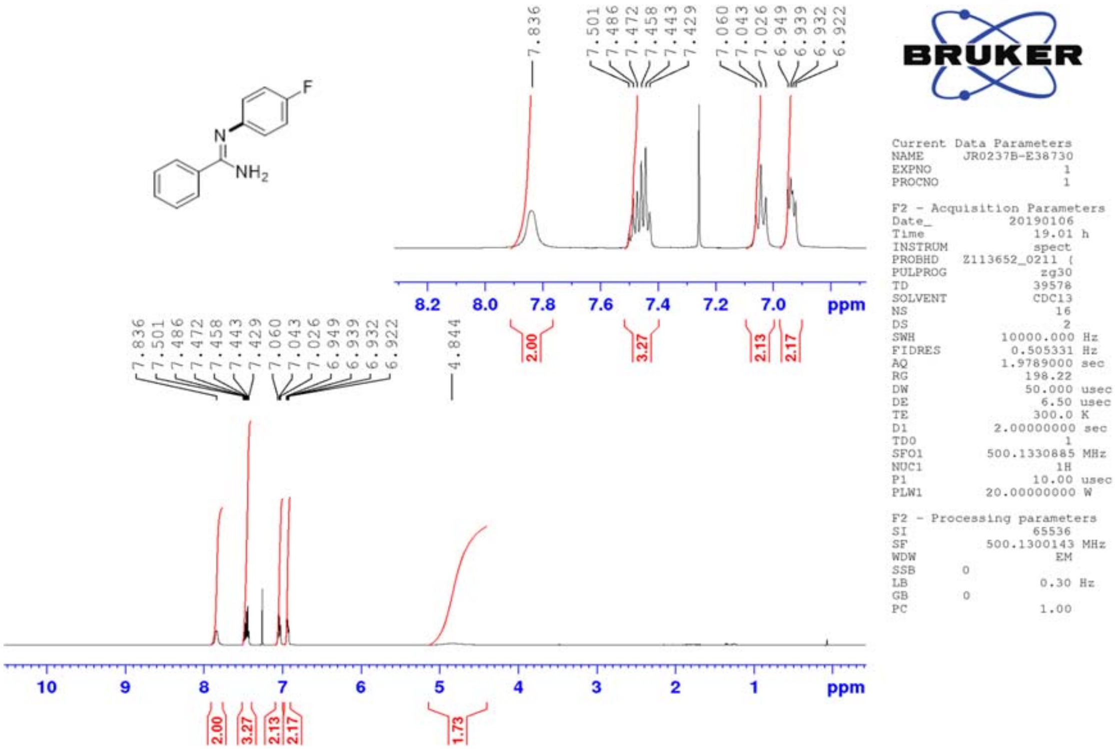

^{13}C NMR

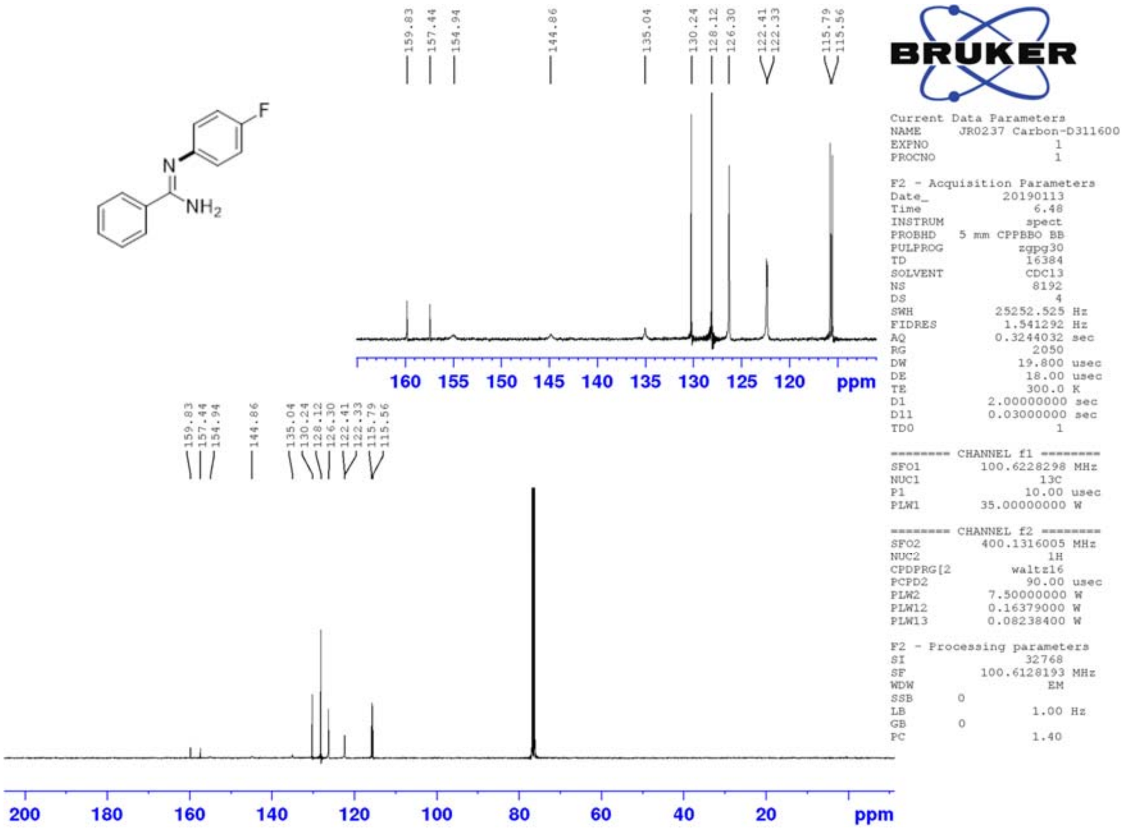

^{19}F NMR

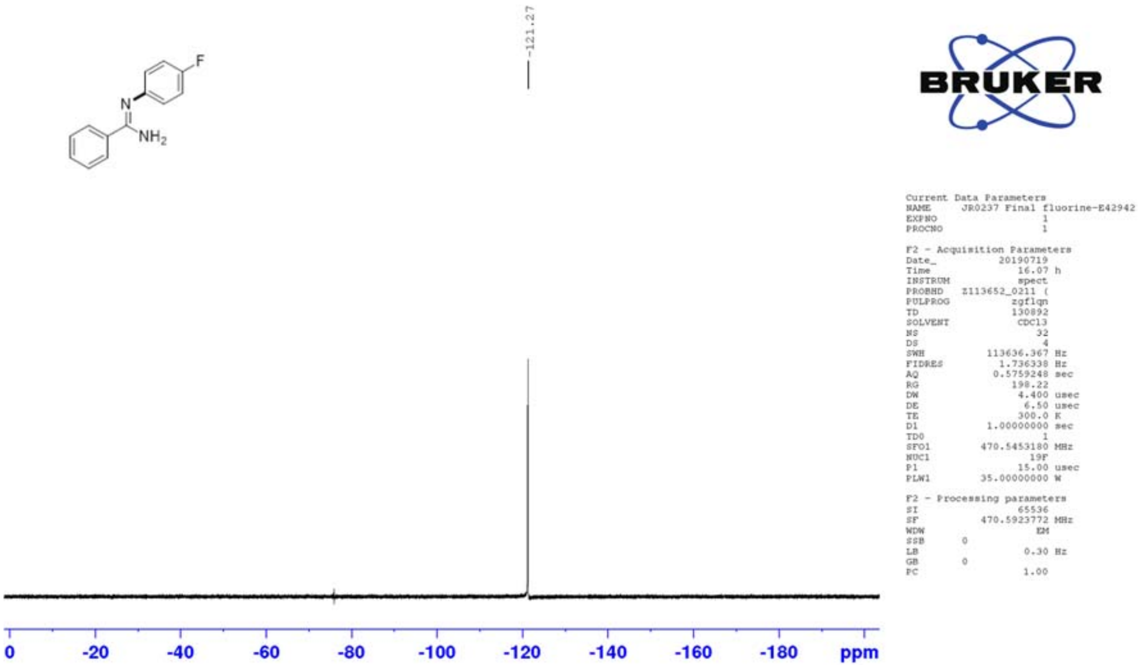

(*Z*)-*N'*-(4-(methylthio)phenyl)benzimidamide (**8**) - ^{1}H NMR

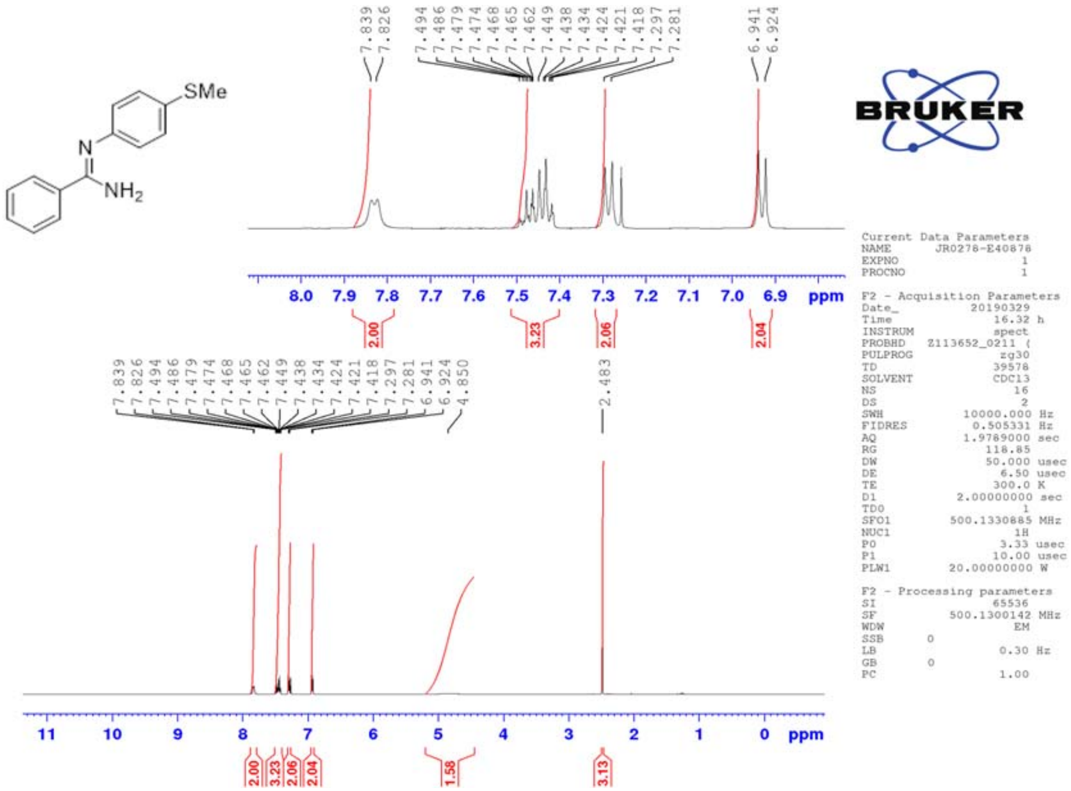

^{13}C NMR

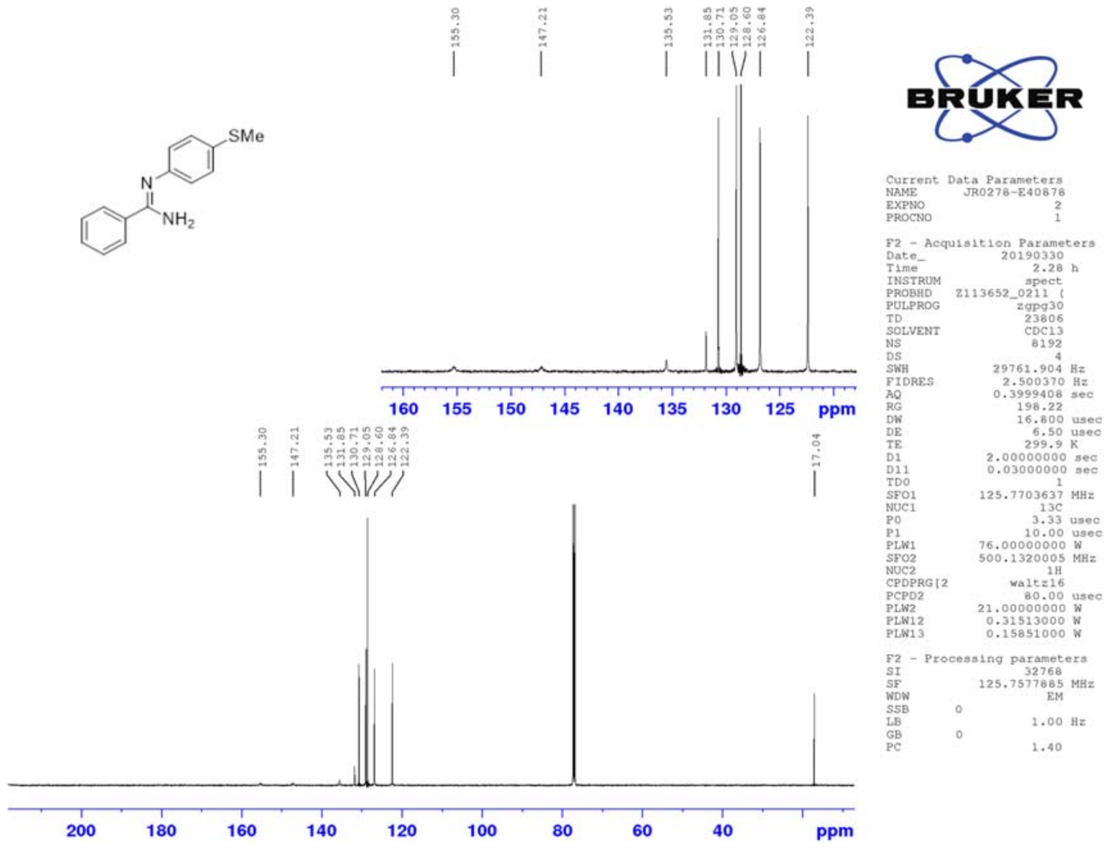

(*Z*)-*N'*-(4-(tert-butyl)phenyl)benzimidamide (**9**) - ^{1}H NMR

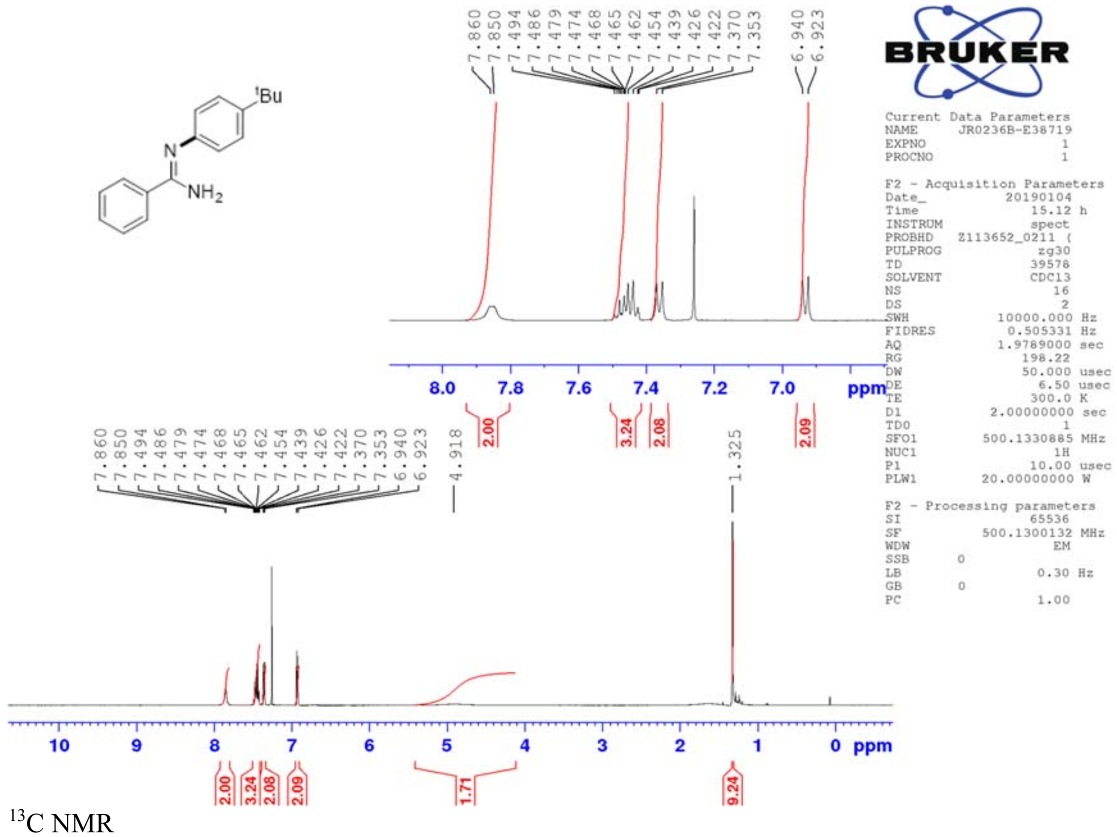

^{13}C NMR

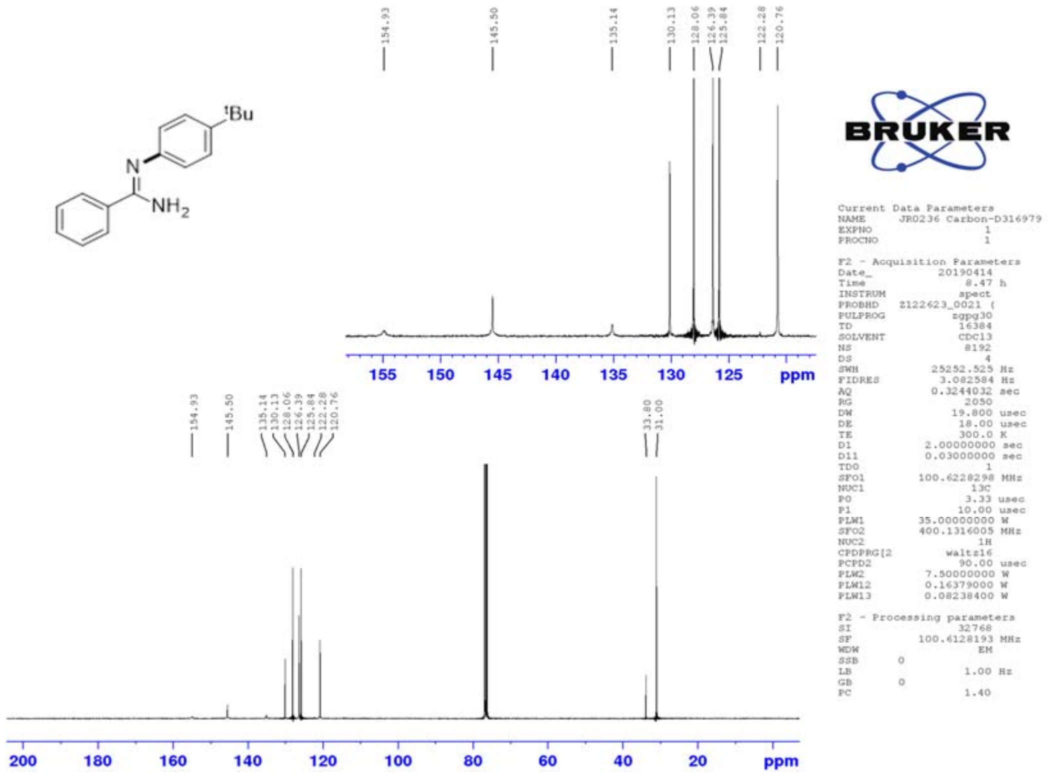

(*Z*)-*N*'-(4-(trifluoromethyl)phenyl)benzimidamide (**10**) - ^{1}H NMR

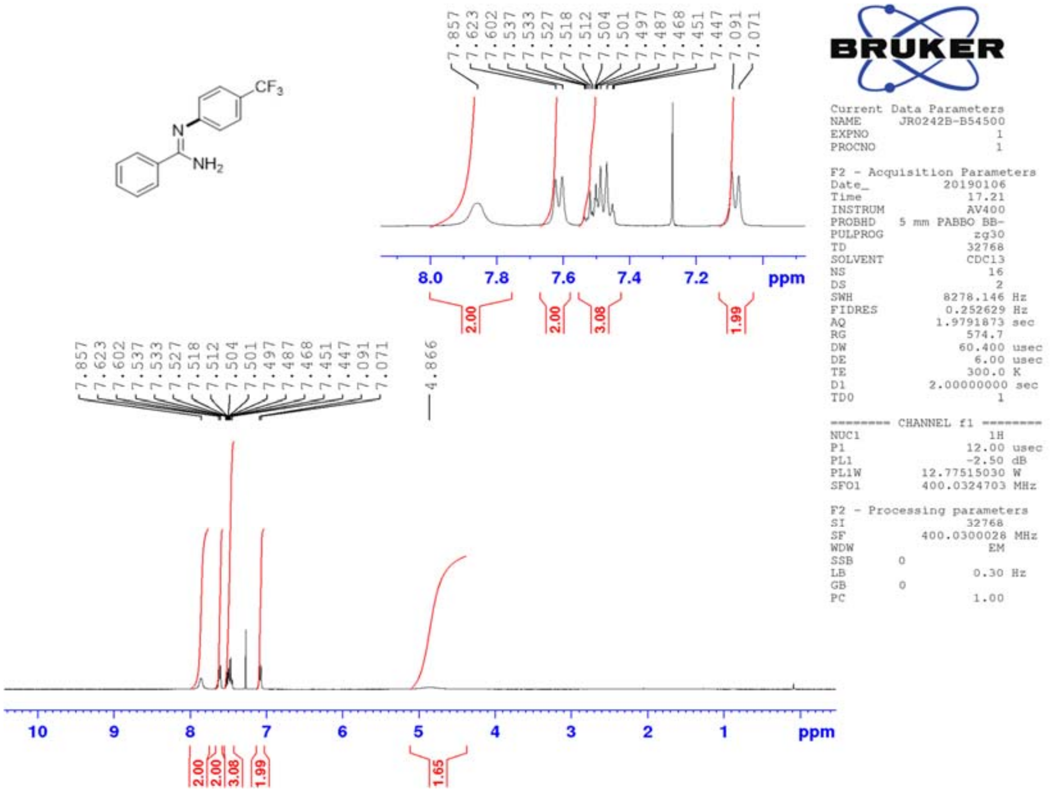

^{13}C NMR

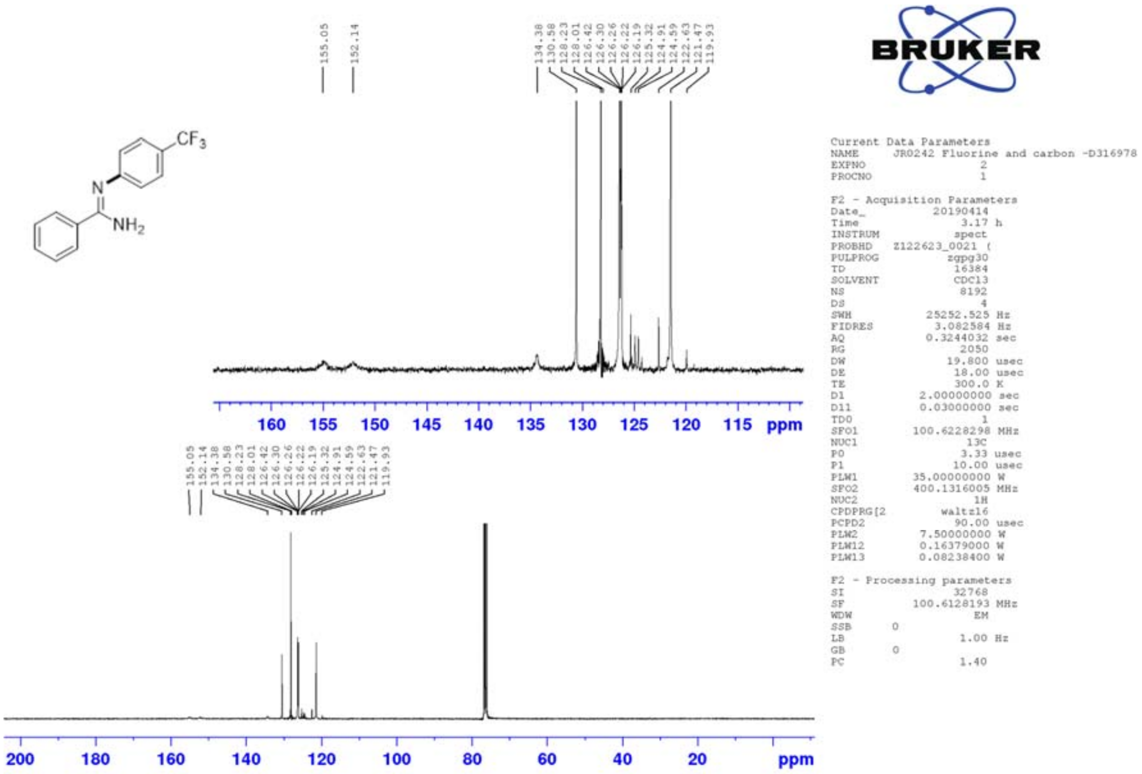

^{19}F NMR

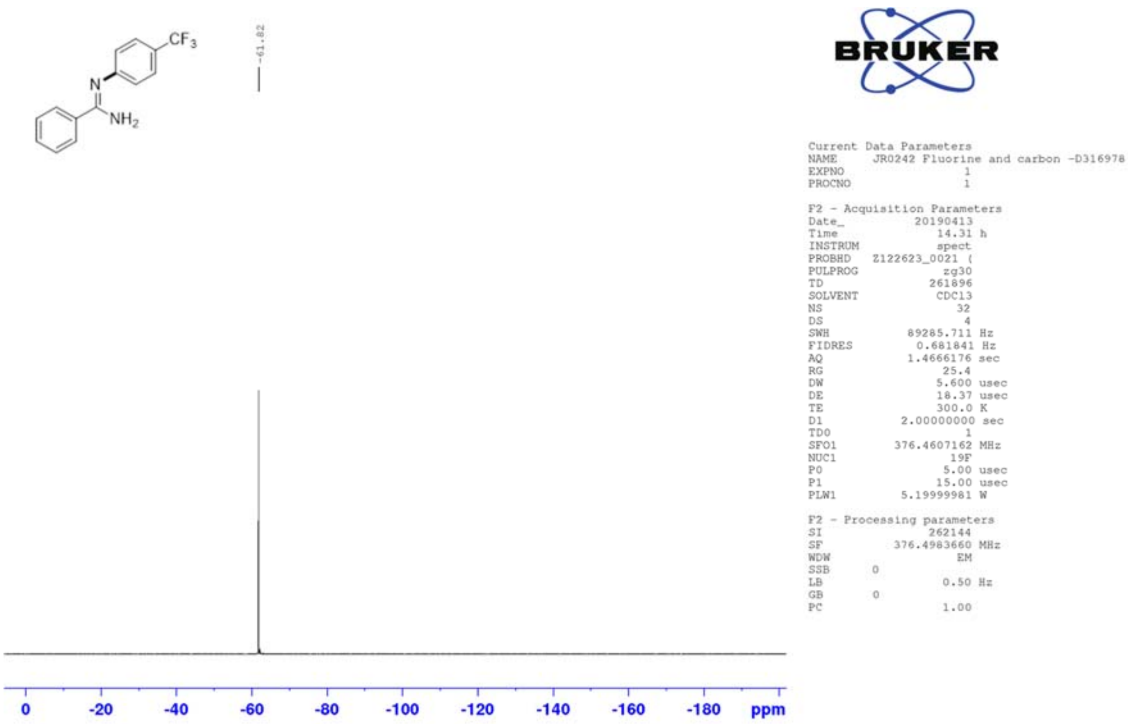

(Z)-*N'*-(m-tolyl)benzimidamide (**11**) - ^{1}H NMR

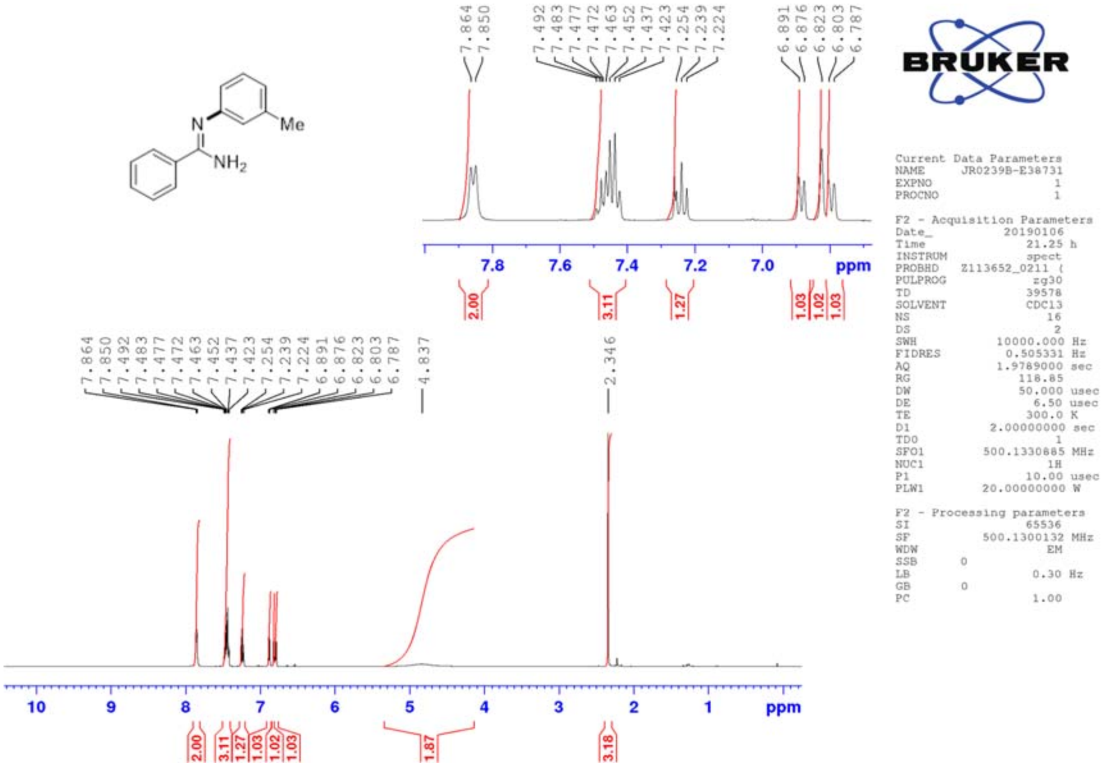

^{13}C NMR

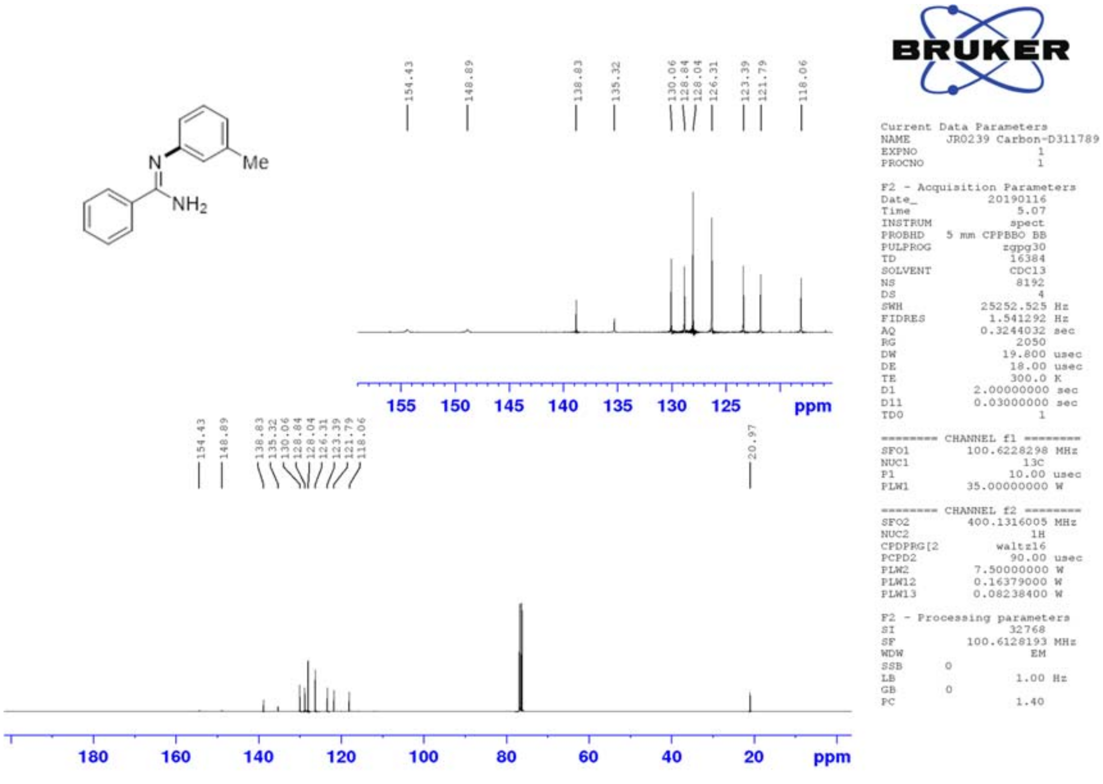

(Z)-*N'*-([1,1'-biphenyl]-4-yl)benzimidamide (**12**) - ^{1}H NMR

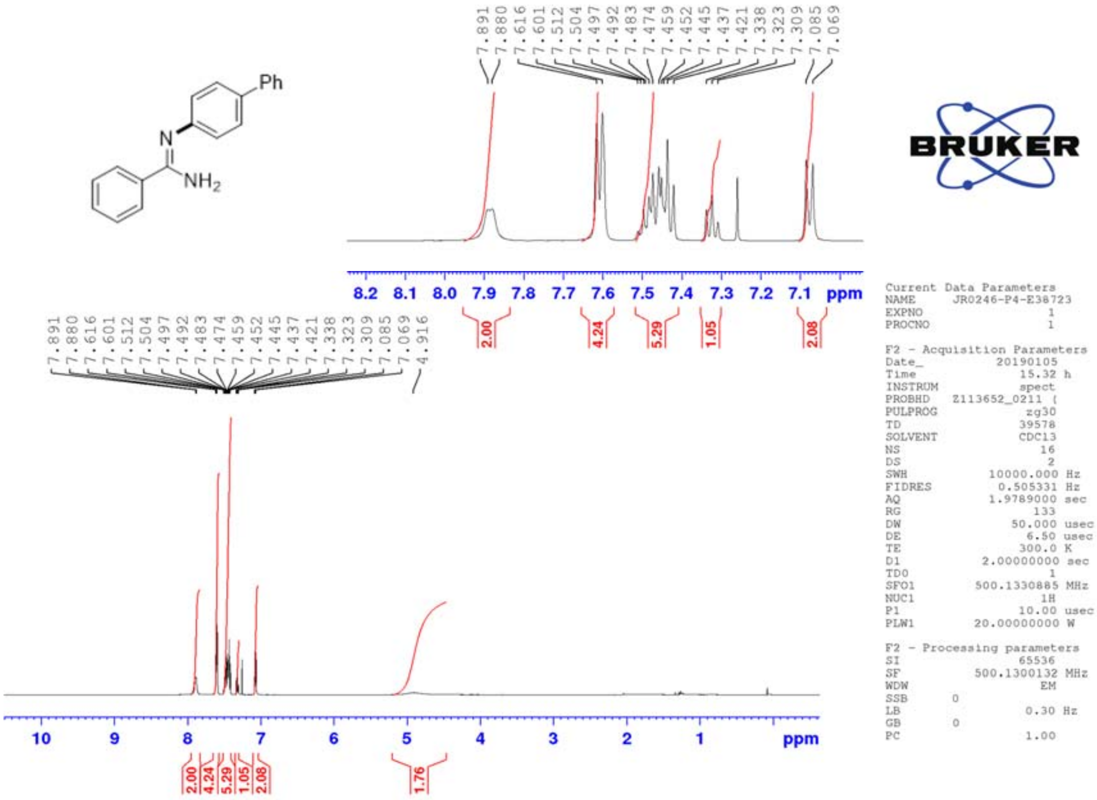

^{13}C NMR

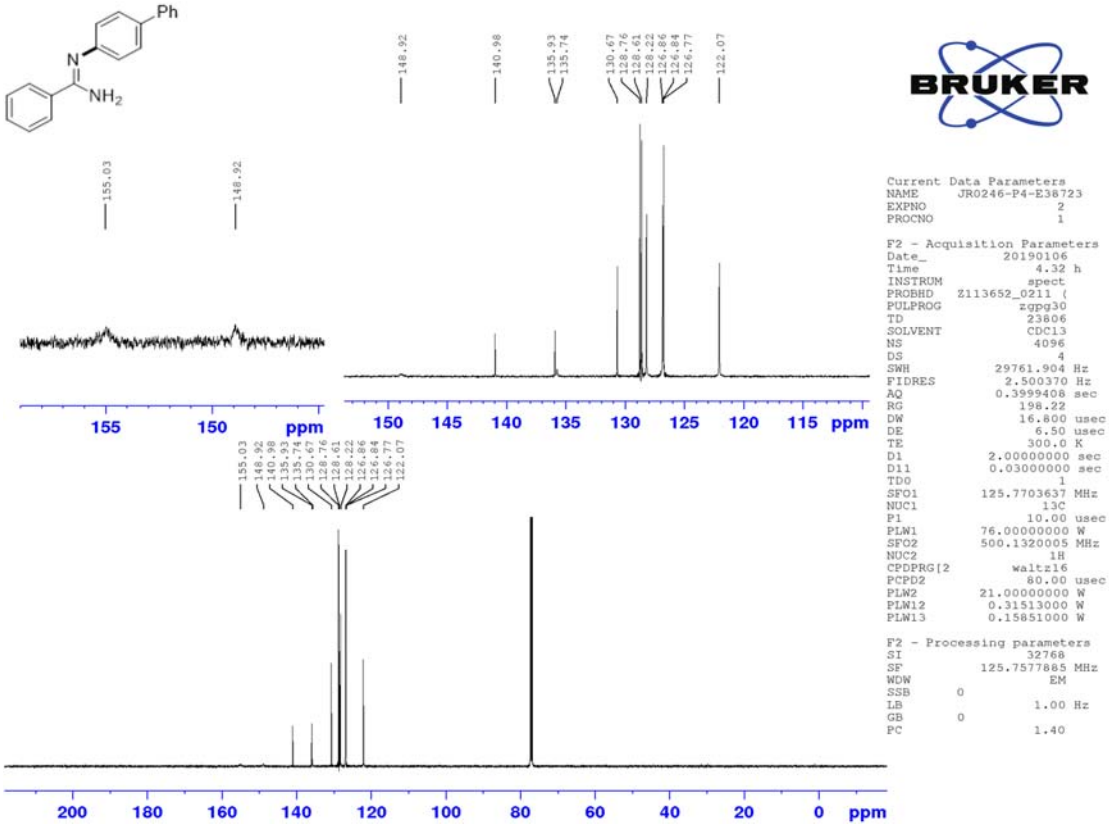

(Z)-*N'*-(o-tolyl)benzimidamide (**13**) - ^{1}H NMR

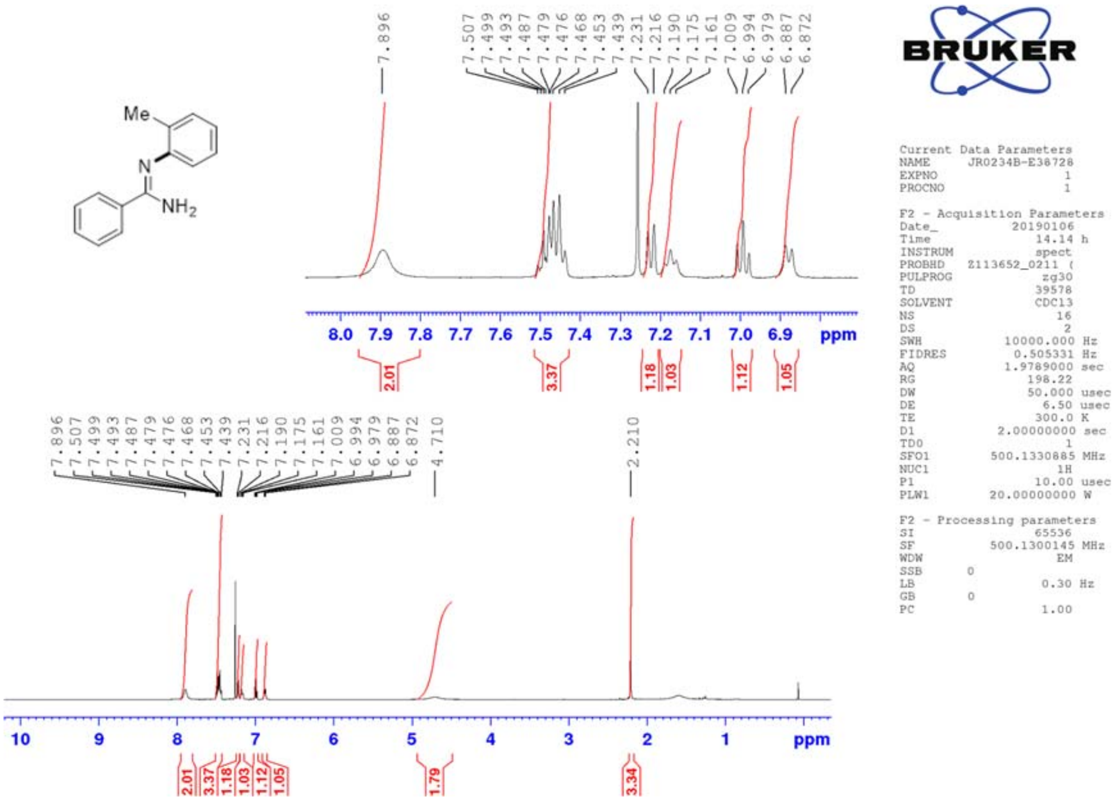

^{13}C NMR

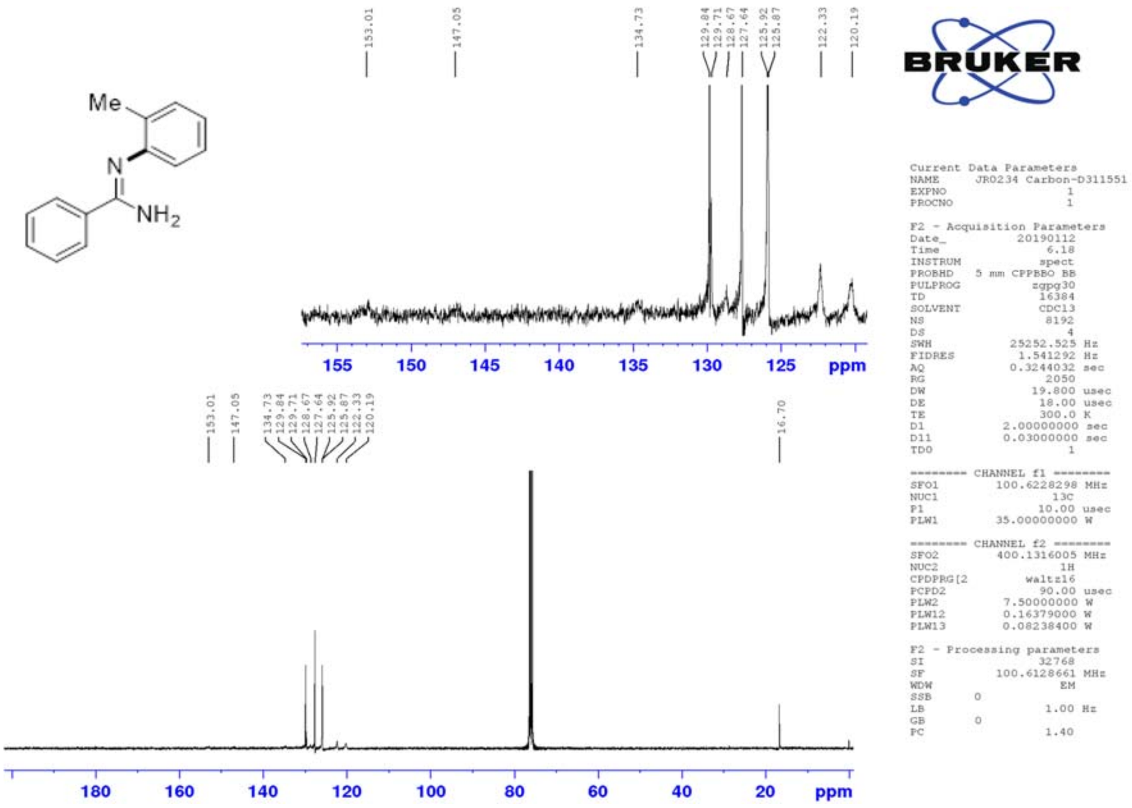

(Z)-*N'*-(2-methoxyphenyl)benzimidamide (**14**) - ^{1}H NMR

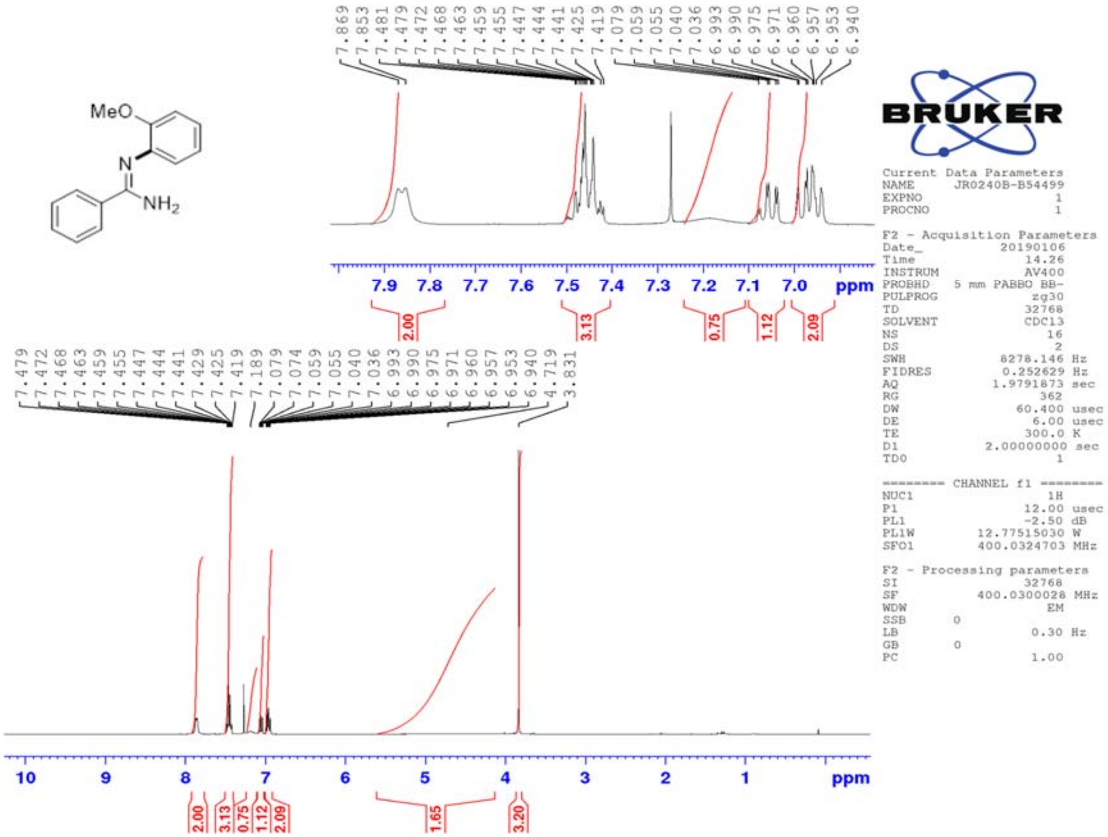

^{13}C NMR

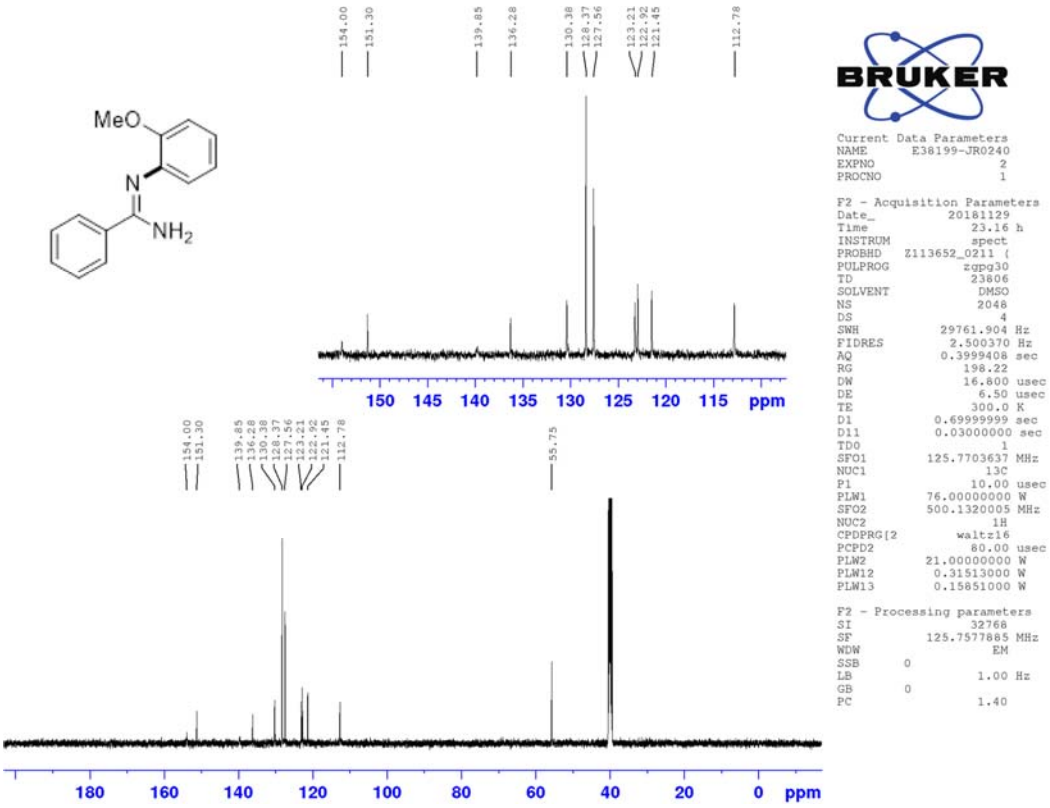

(*Z*)-*N'*-(pyridin-3-yl)benzimidamide trifluoroacetate (**15**) - ^{1}H NMR

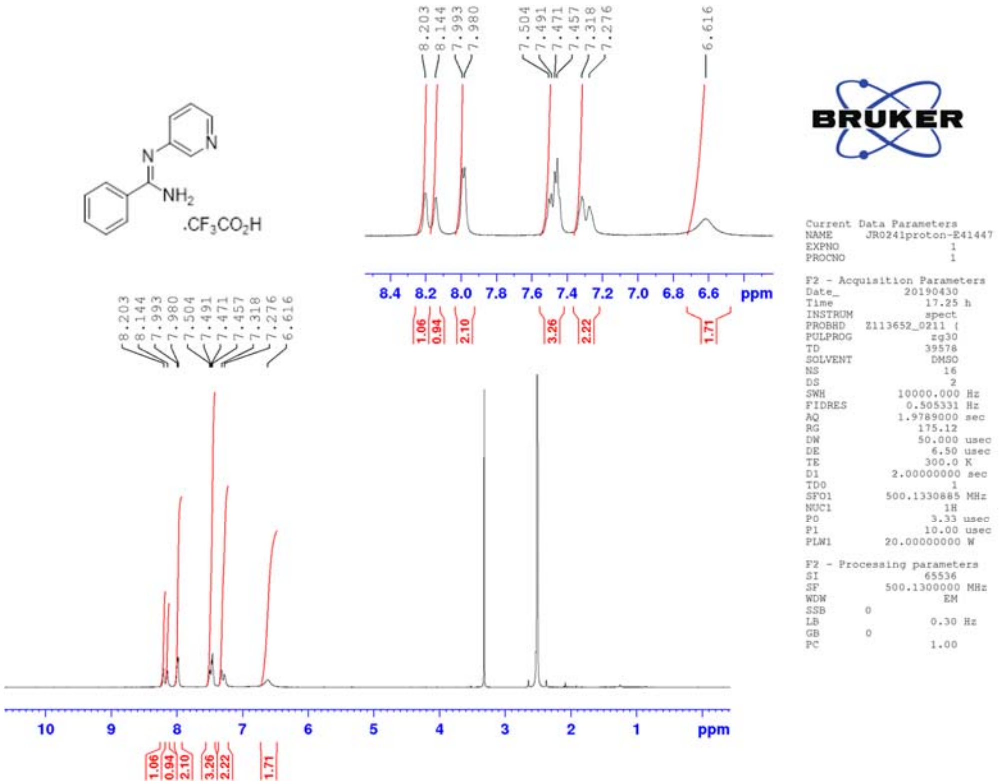

^{13}C NMR

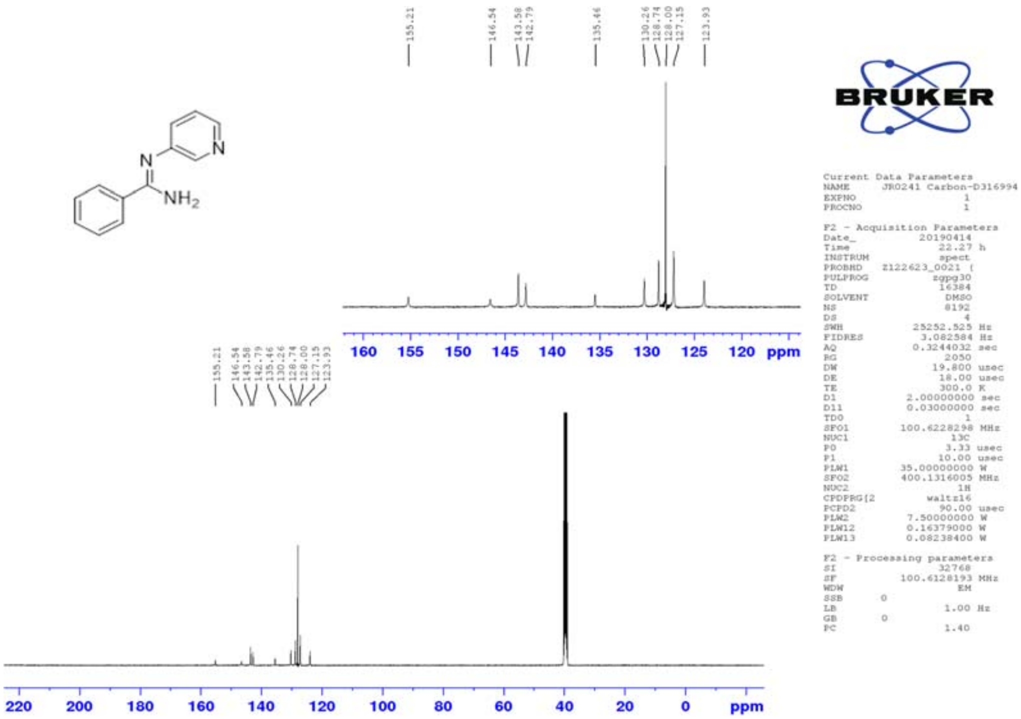

^{19}F NMR

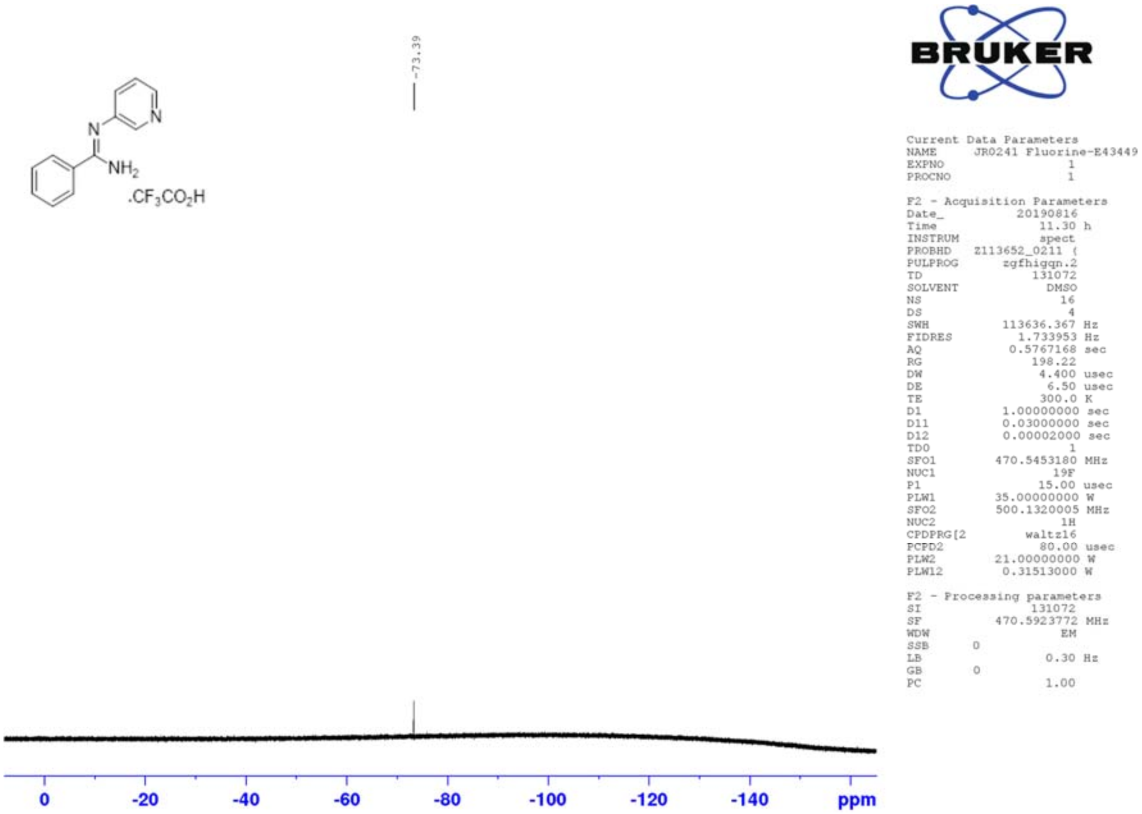

(*Z*)-*N'*-(3-fluoro-4-methoxyphenyl)benzimidamide (**16**) - ^{1}H NMR

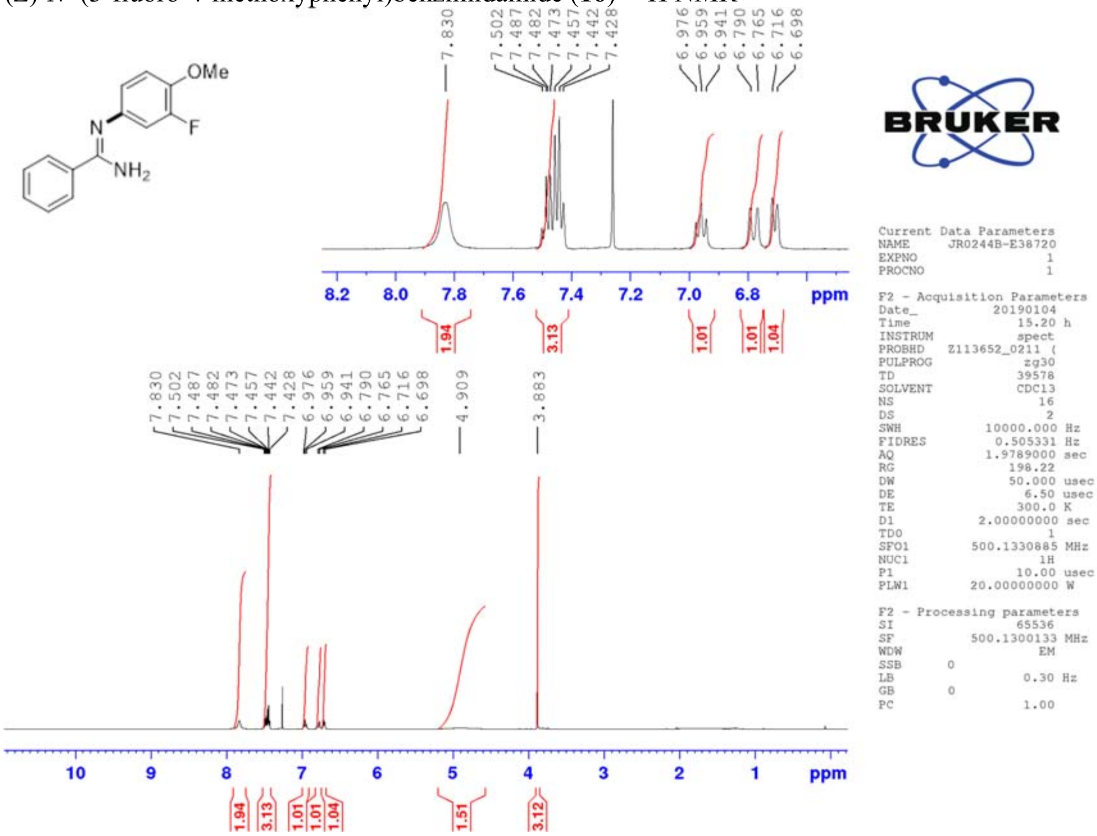

^{13}C NMR

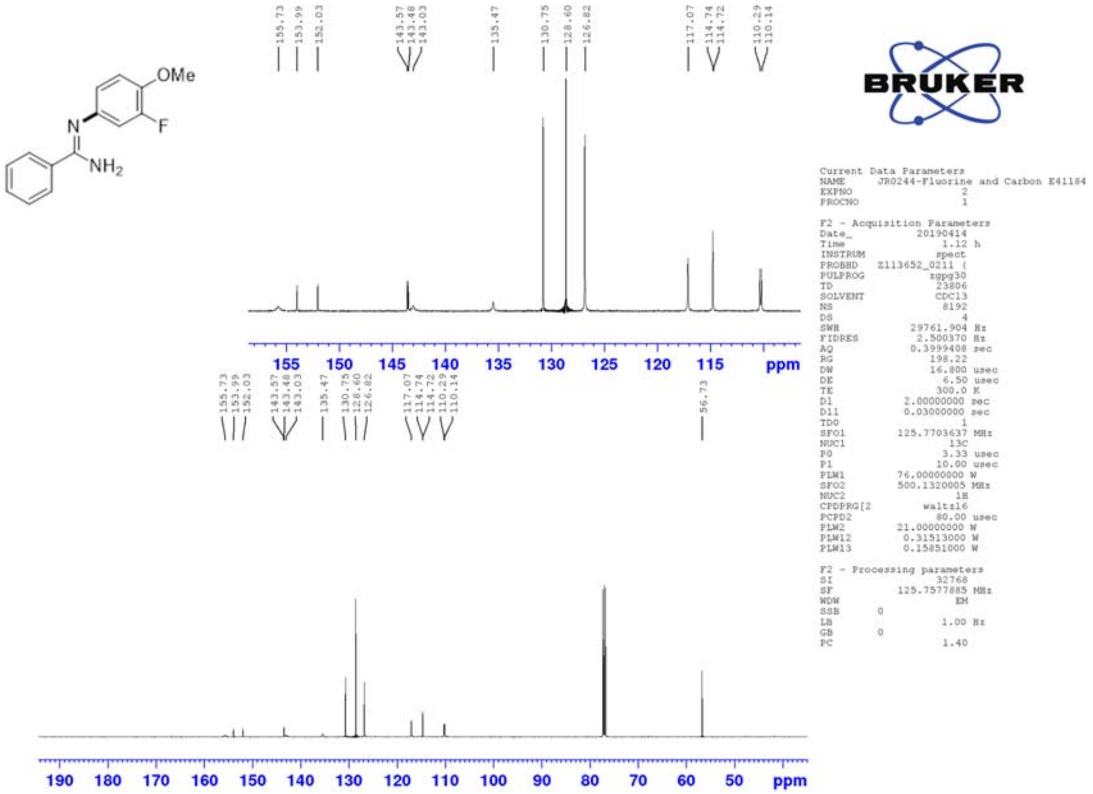

^{19}F NMR

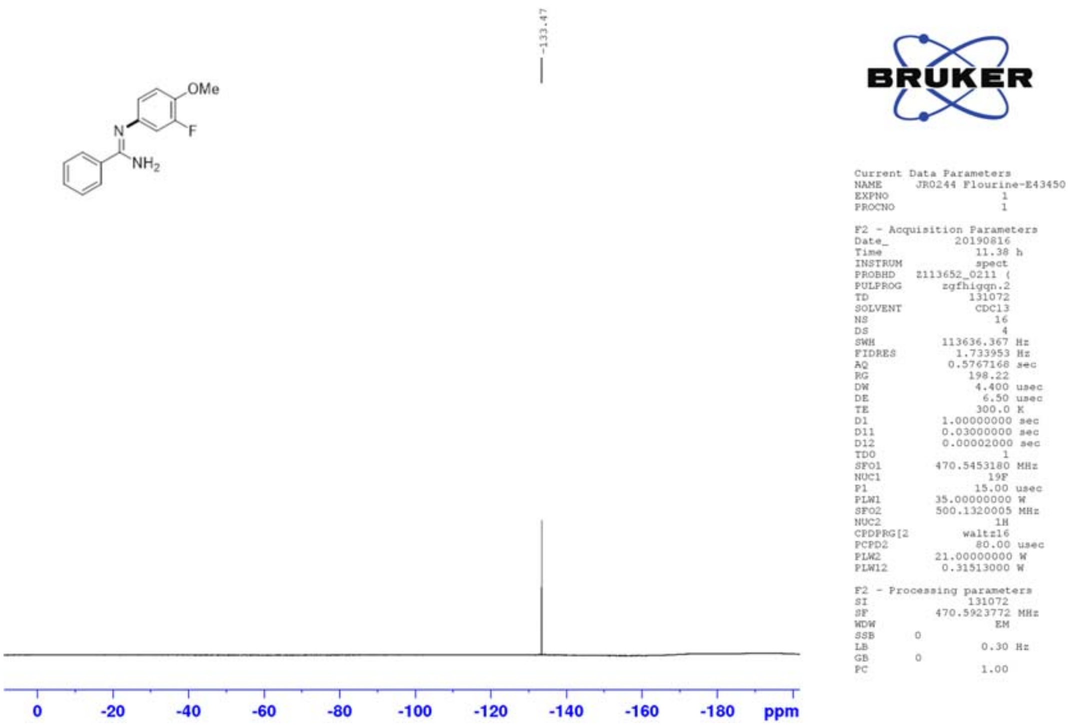

(*Z*)-*N'*-(3-methoxyphenyl)benzimidamide (**17**) - ^{1}H NMR

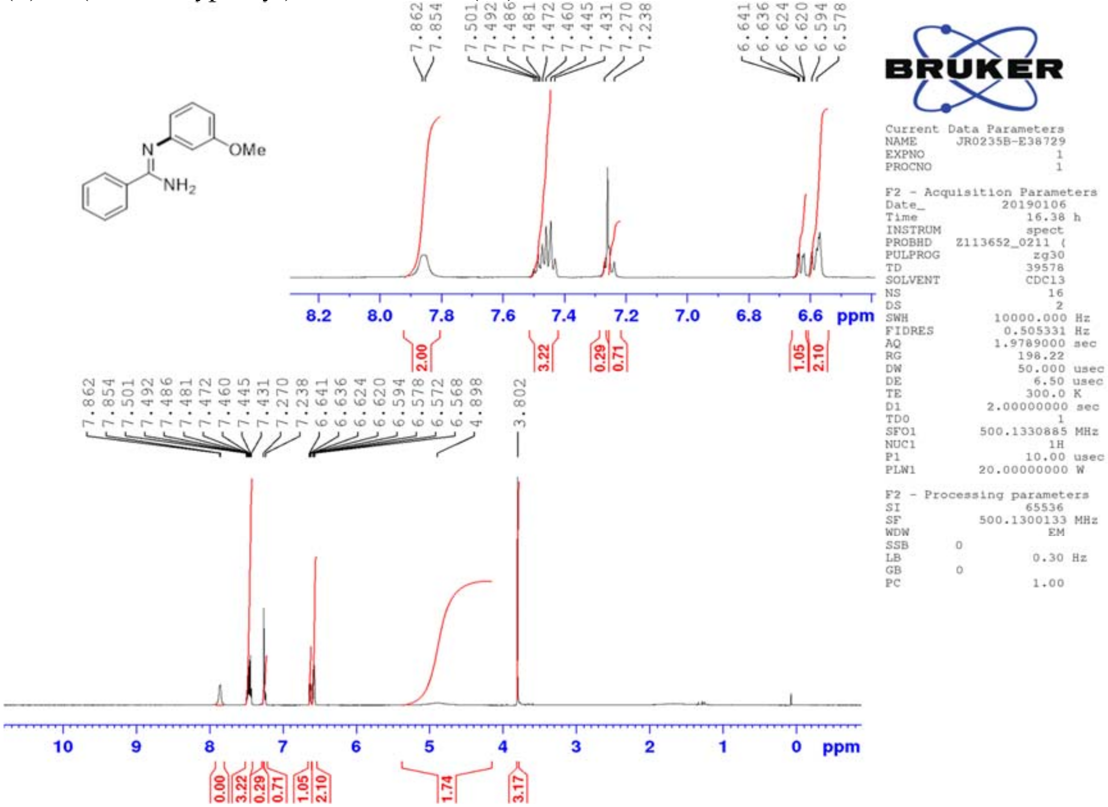

^{13}C NMR

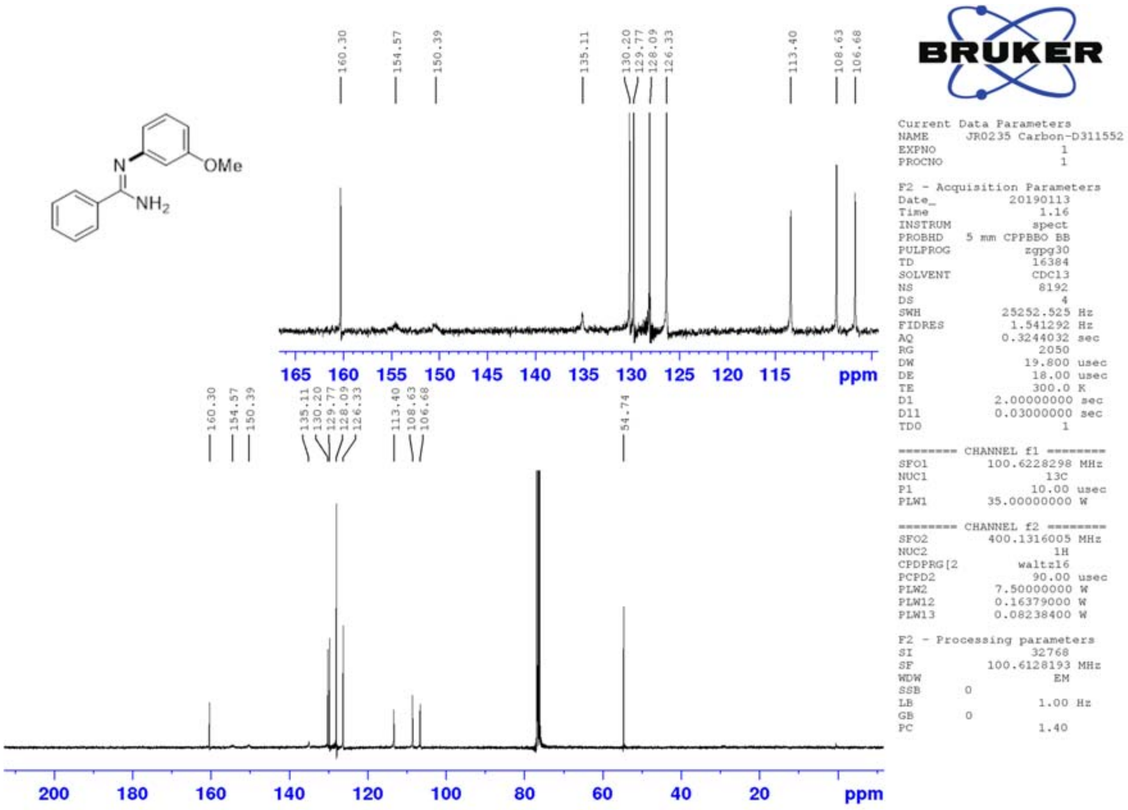

(*Z*)-*N'*-(4-methoxy-3,5-dimethylphenyl)benzimidamide (**18**) - ^{1}H NMR

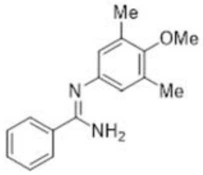

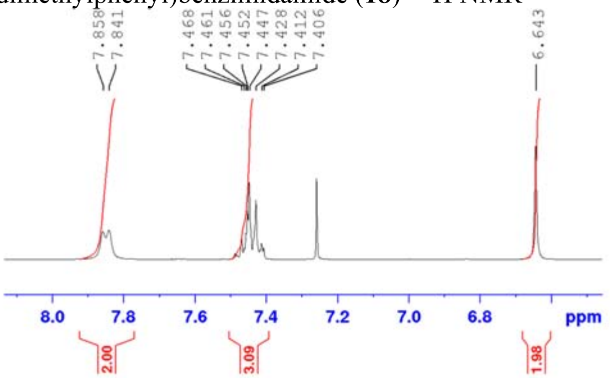

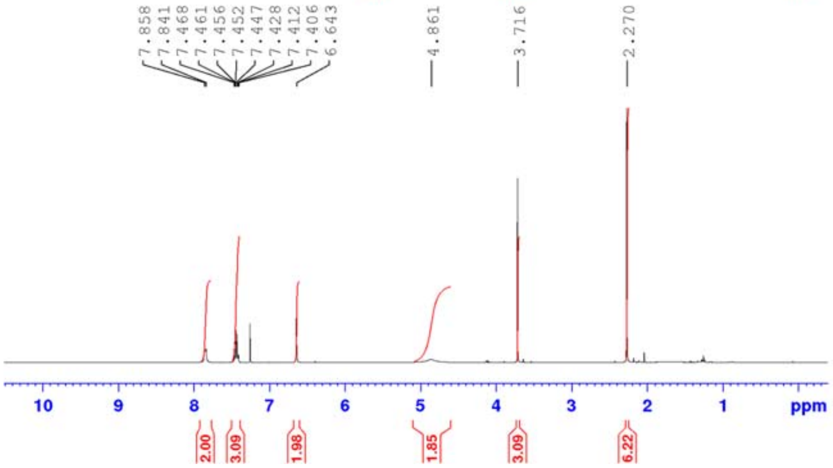


```
Current Data Parameters
NAME              D321451
EXPNO                   1
PROCNO                  1

F2 - Acquisition Parameters
Date_            20190714
Time                19.27 h
INSTRUM             spect
PROBHD   Z122623_0021 (
PULPROG              zg30
TD                  32564
SOLVENT             CDCl3
NS                      4
DS                      2
SWH              8223.685 Hz
FIDRES           0.505078 Hz
AQ              1.9798912 sec
RG                    128
DW                 60.800 usec
DE                  10.00 usec
TE                  300.0 K
D1             2.00000000 sec
TD0                     1
SFO1         400.1324710 MHz
NUC1                   1H
P0                   4.00 usec
P1                  12.00 usec
PLW1           7.19999981 W

F2 - Processing parameters
SI                  32768
SF           400.1300108 MHz
WDW                    EM
SSB      0
LB                   0.30 Hz
GB       0
PC                   1.00
```

^{13}C NMR

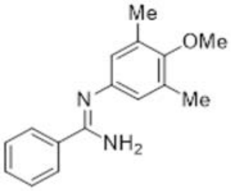

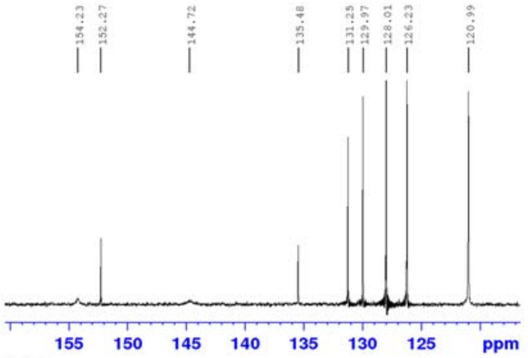

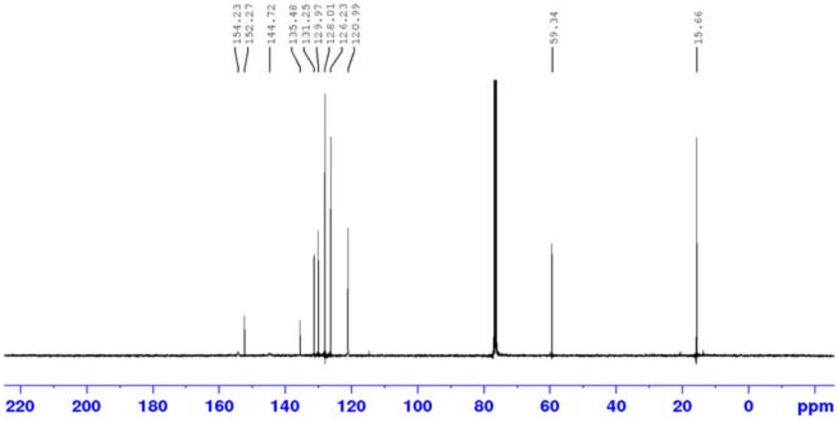


```
Current Data Parameters
NAME              D321451
EXPNO                   3
PROCNO                  1

F2 - Acquisition Parameters
Date_            20190714
Time                23.10 h
INSTRUM             spect
PROBHD   Z122623_0021 (
PULPROG            zgpg30
TD                  16384
SOLVENT             CDCl3
NS                   4096
DS                      4
SWH             25252.525 Hz
FIDRES           3.082584 Hz
AQ              0.3244032 sec
RG                   2050
DW                 19.800 usec
DE                  18.00 usec
TE                  300.0 K
D1             2.00000000 sec
D11            0.03000000 sec
TD0                     1
SFO1         100.6228298 MHz
NUC1                  13C
P0                   3.33 usec
P1                  10.00 usec
PLW1          35.00000000 W
SFO2         400.1316005 MHz
NUC2                   1H
CPDPRG[2          waltz16
PCPD2               90.00 usec
PLW2           7.50000000 W
PLW12          0.16379000 W
PLW13          0.08238400 W

F2 - Processing parameters
SI                  32768
SF           100.6128193 MHz
WDW                    EM
SSB      0
LB                   1.00 Hz
GB       0
PC                   1.40
```

(Z)-*N'*-(3,4,5-trifluorophenyl)benzimidamide (**19**) - ^{1}H NMR

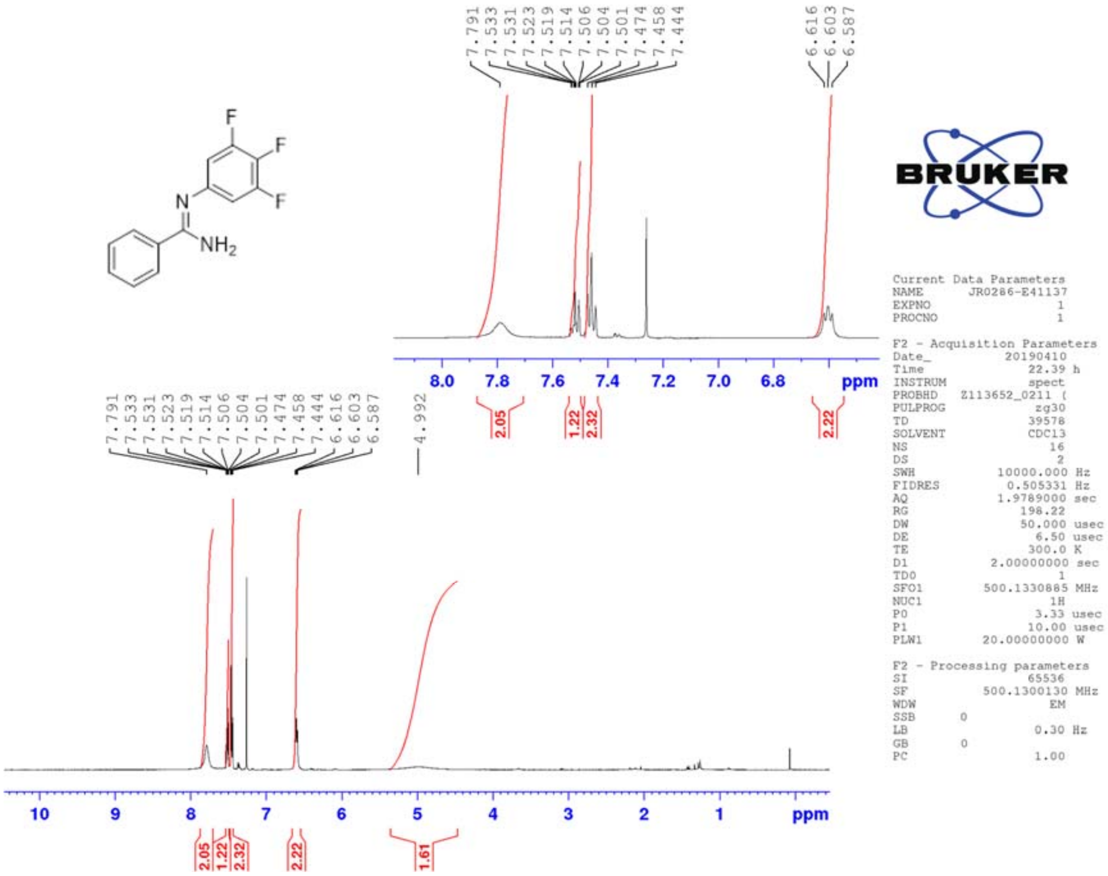

^{13}C NMR

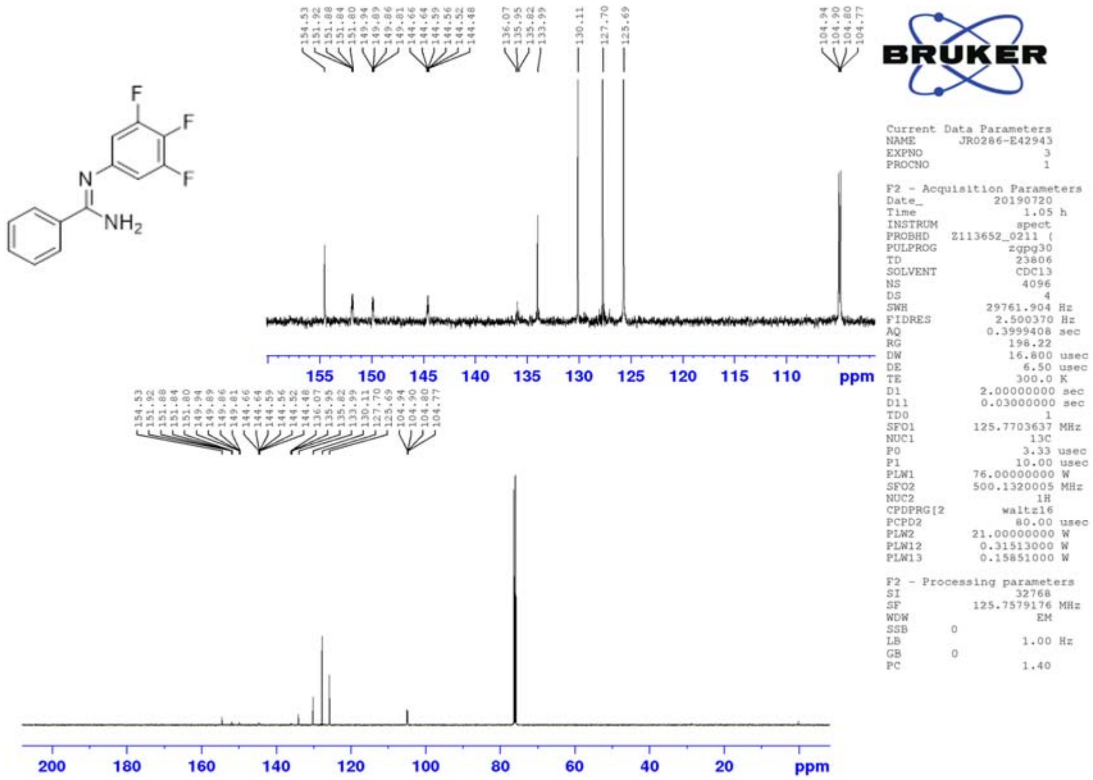

^{19}F NMR

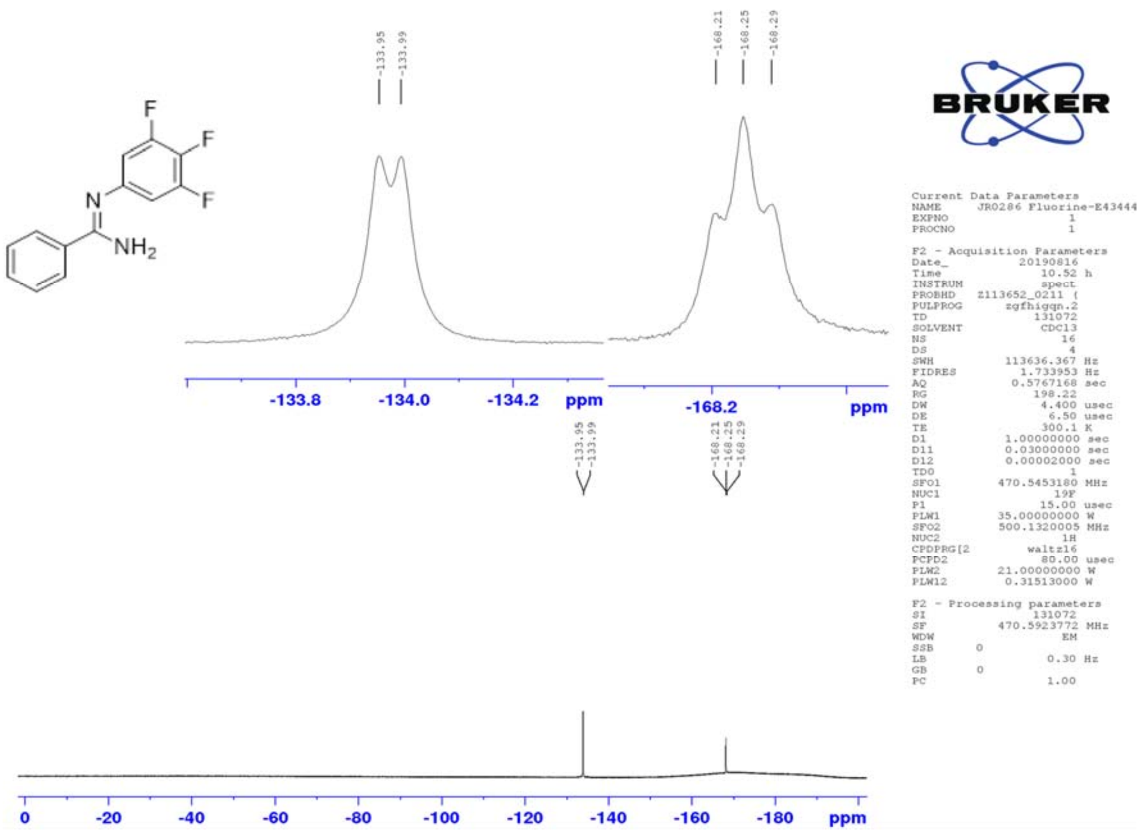

(*Z*)-*N'*-((*E*)-styryl)benzimidamide (**20**) - ^{1}H NMR

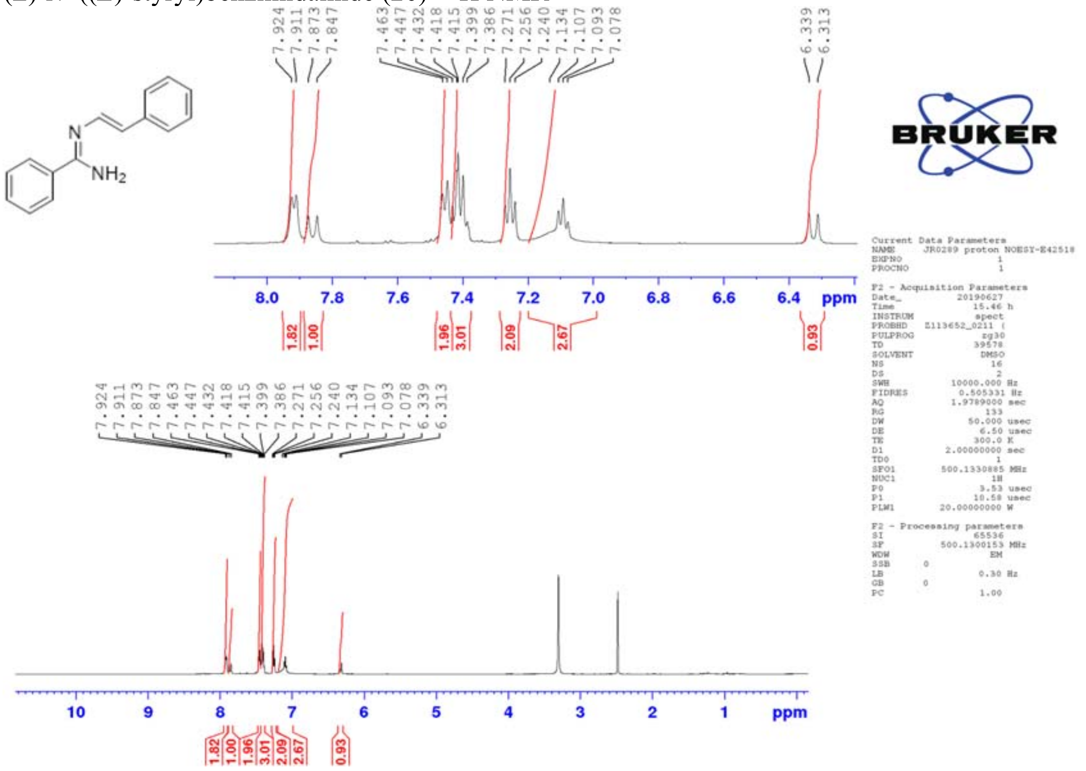

^{13}C NMR

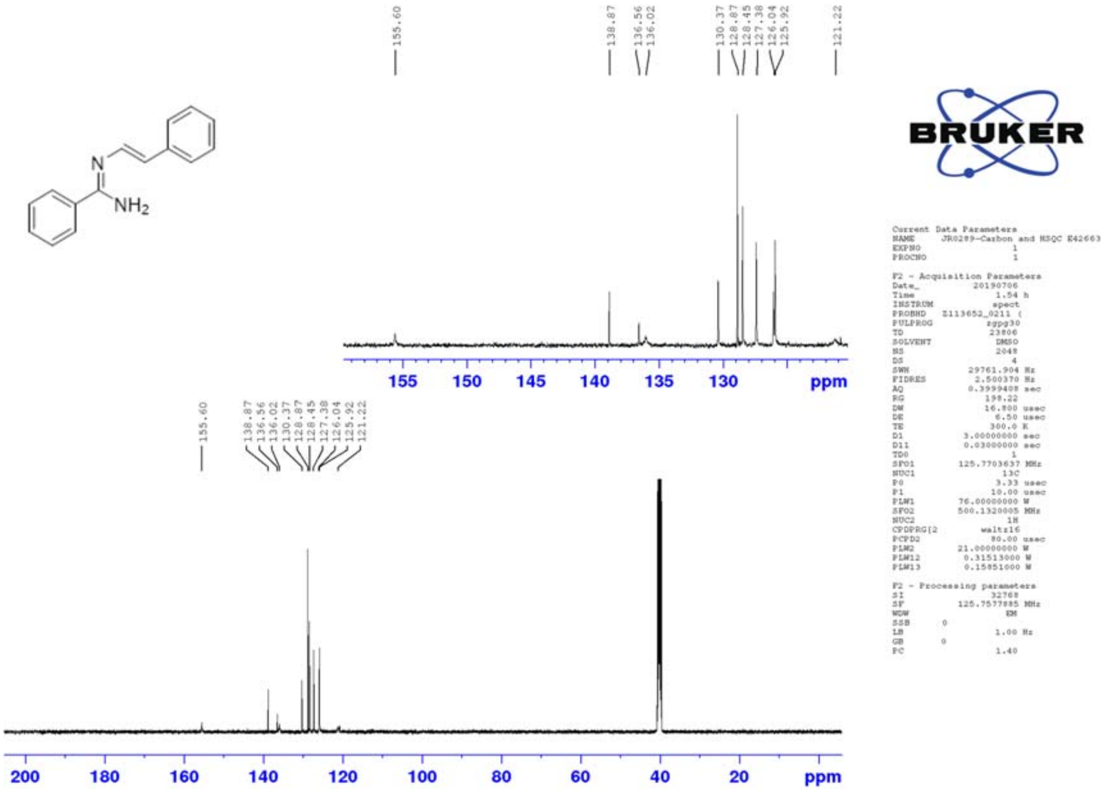

(*Z*)-*N'*-(cyclohex-1-en-1-yl)benzimidamide (**21**) - ^{1}H NMR

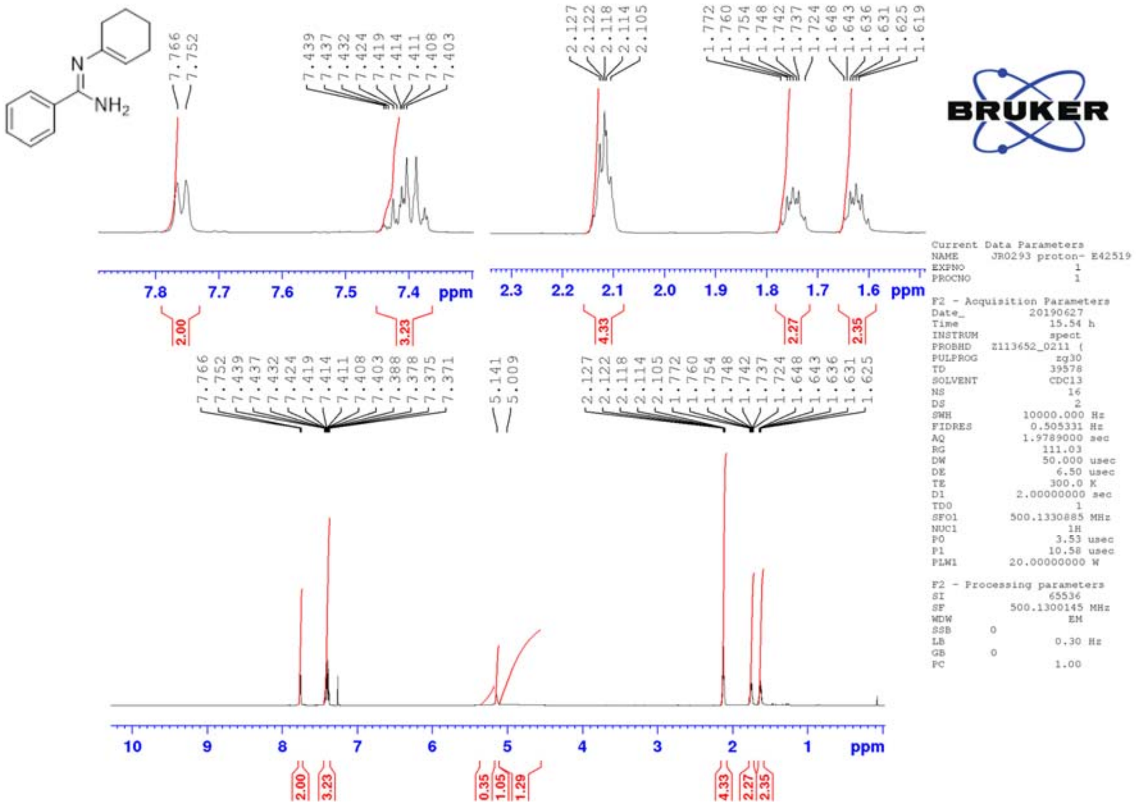

^{13}C NMR

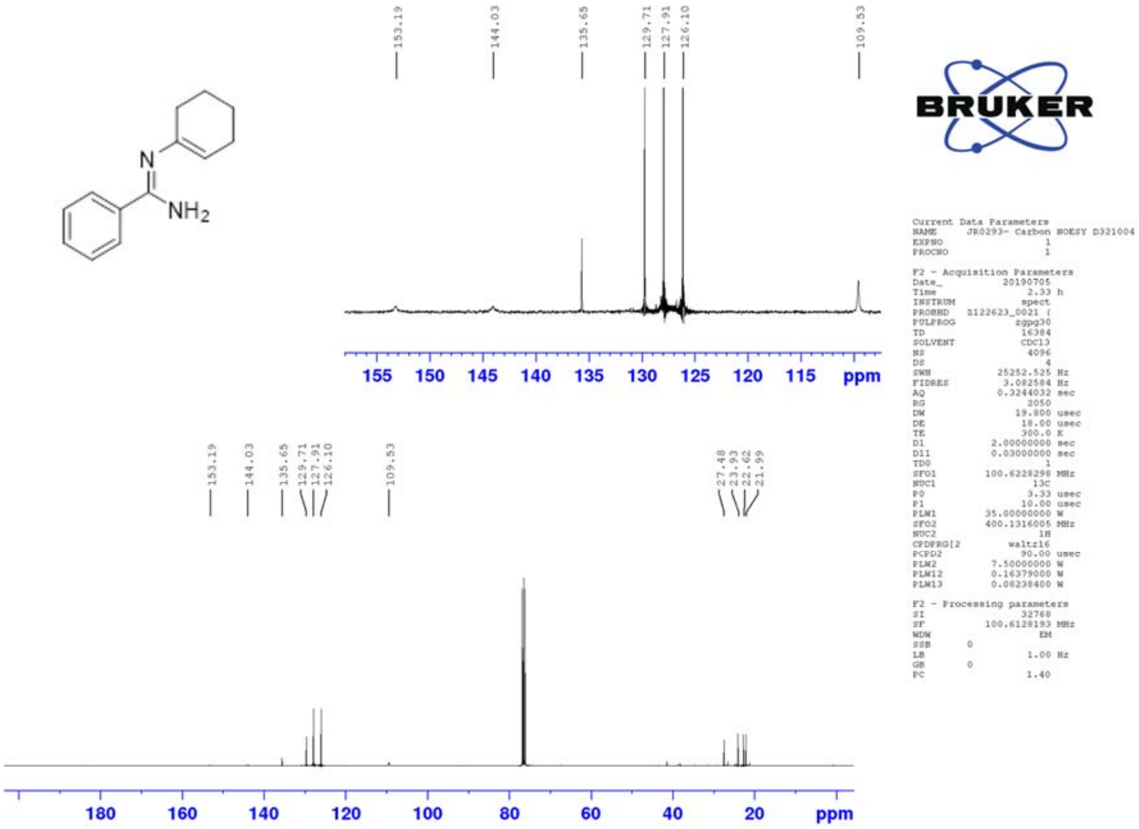

(Z)-4-chloro-*N'*-phenylbenzimidamide (**24**) - ^{1}H NMR

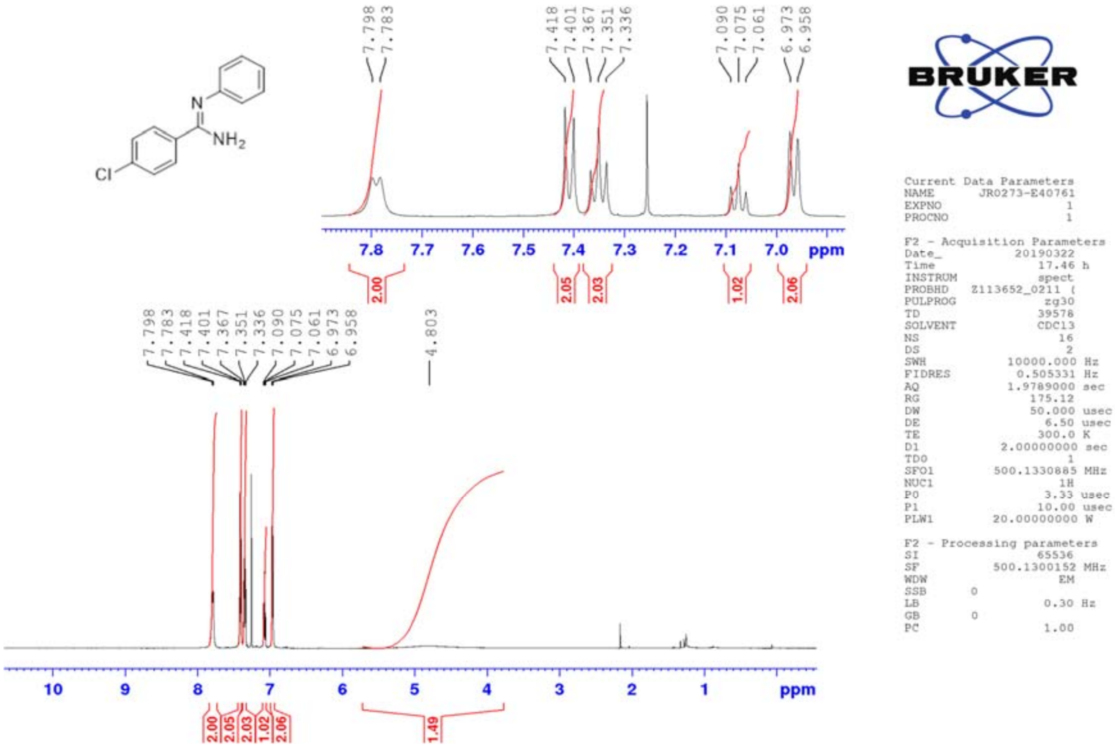

^{13}C NMR

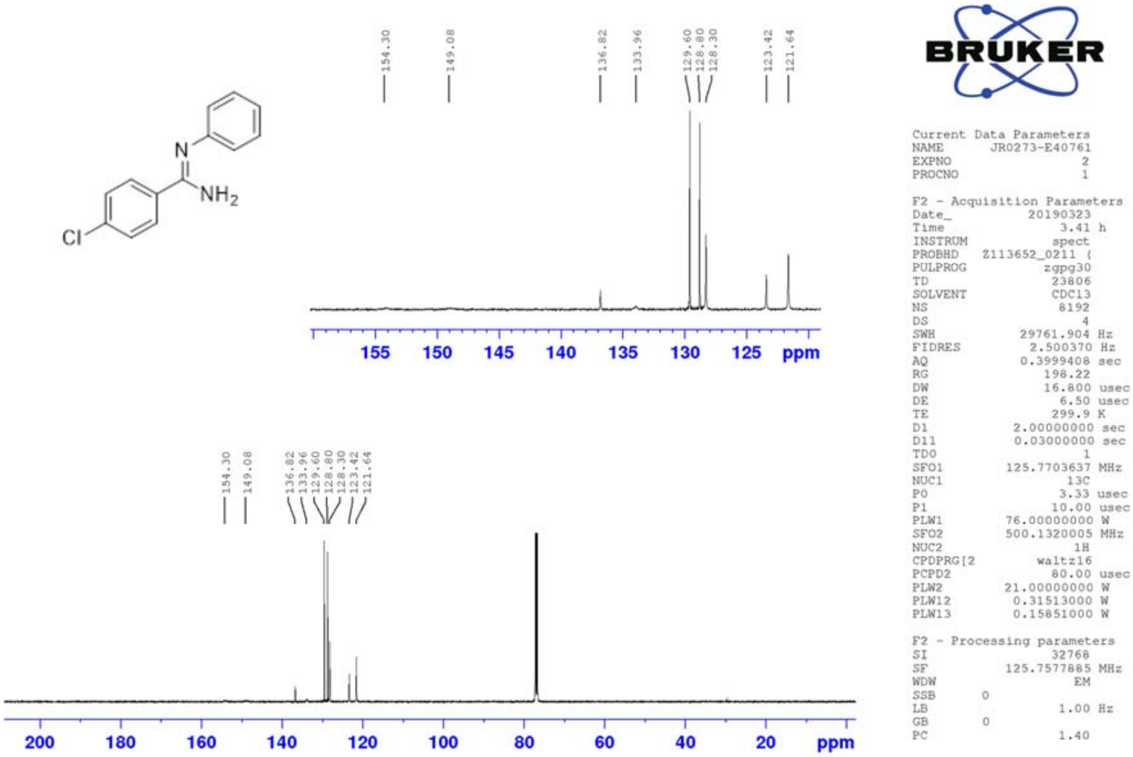

(*Z*)-3-methoxy-*N*'-phenylbenzimidamide (**25**) - ^{1}H NMR

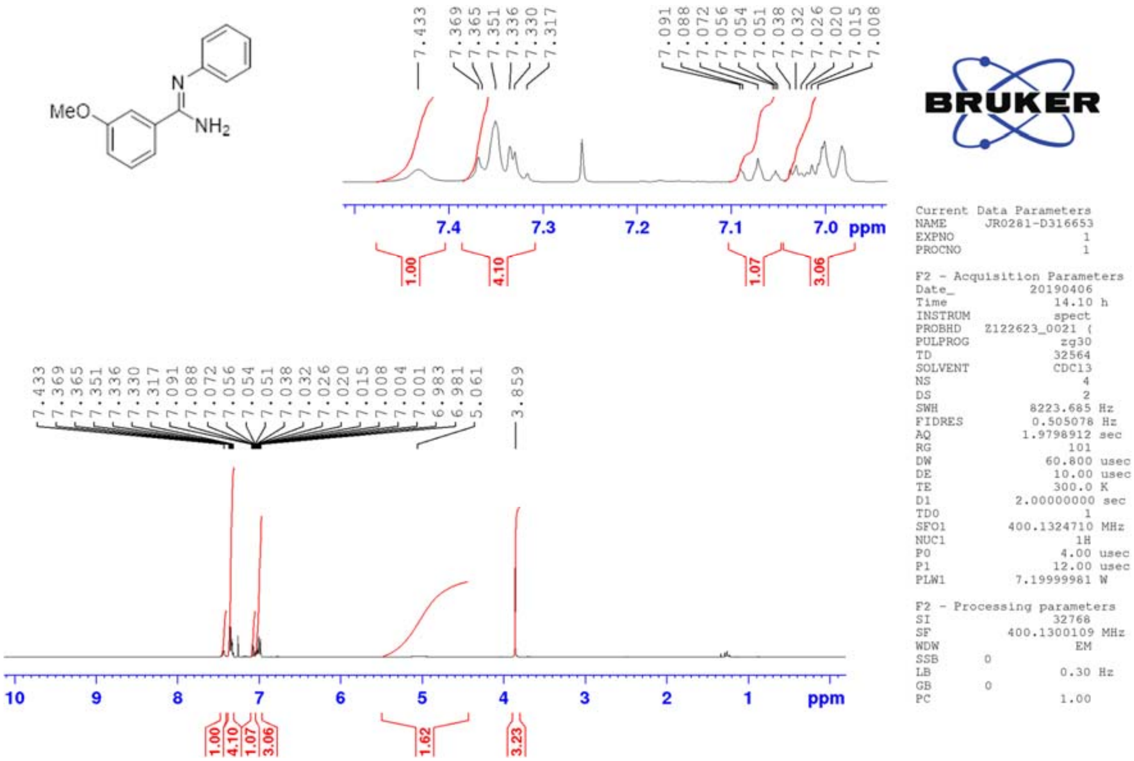

^{13}C NMR

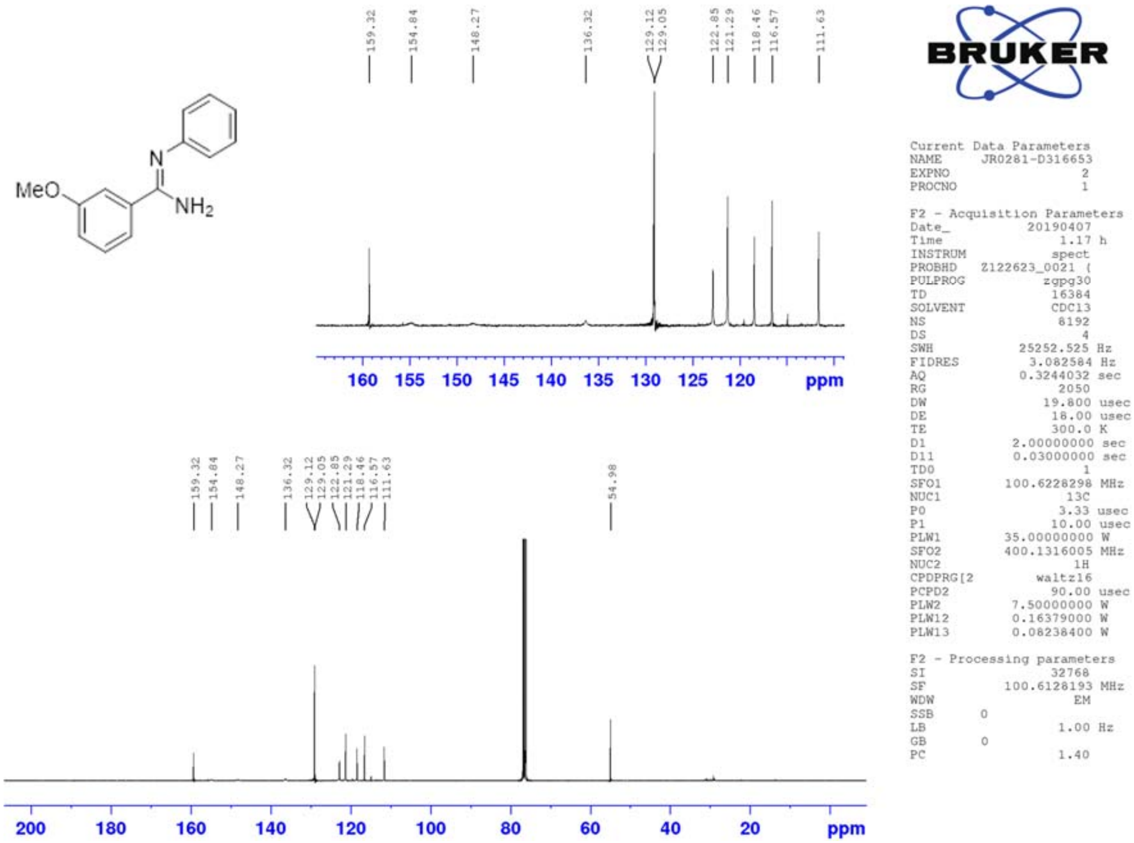

(Z)-2-fluoro-N'-phenylbenzimidamide (**26**) - ^{1}H NMR

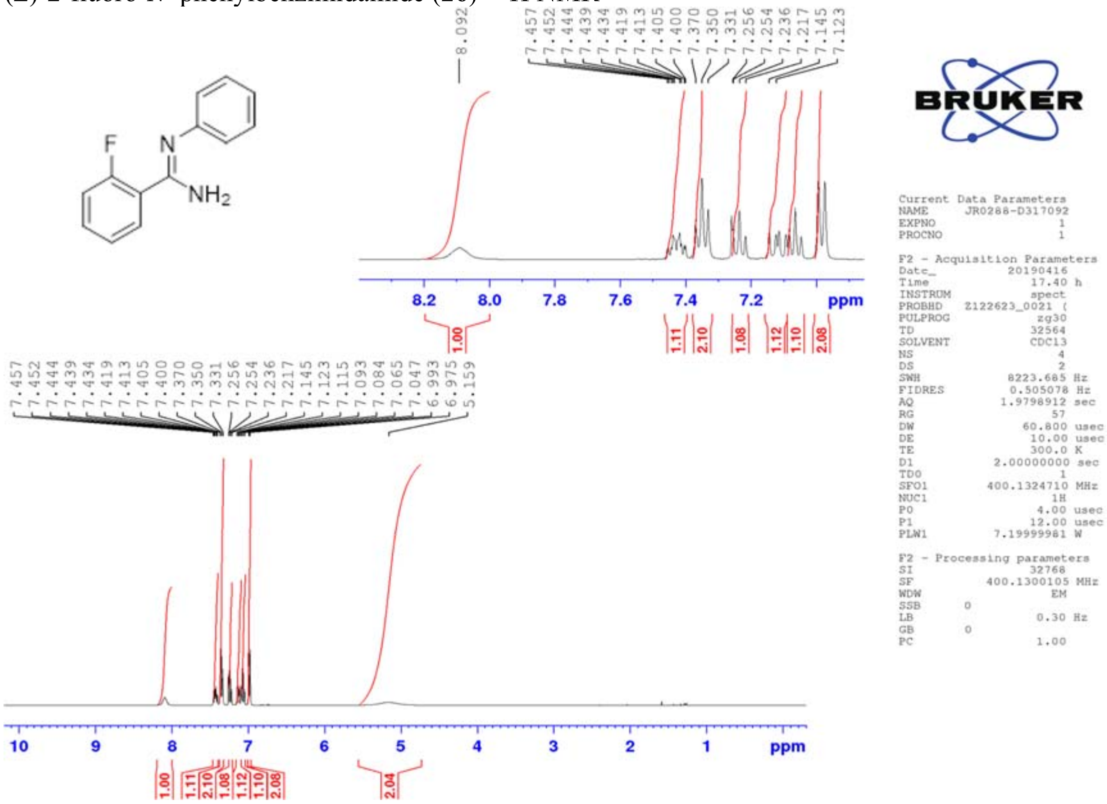

^{13}C NMR

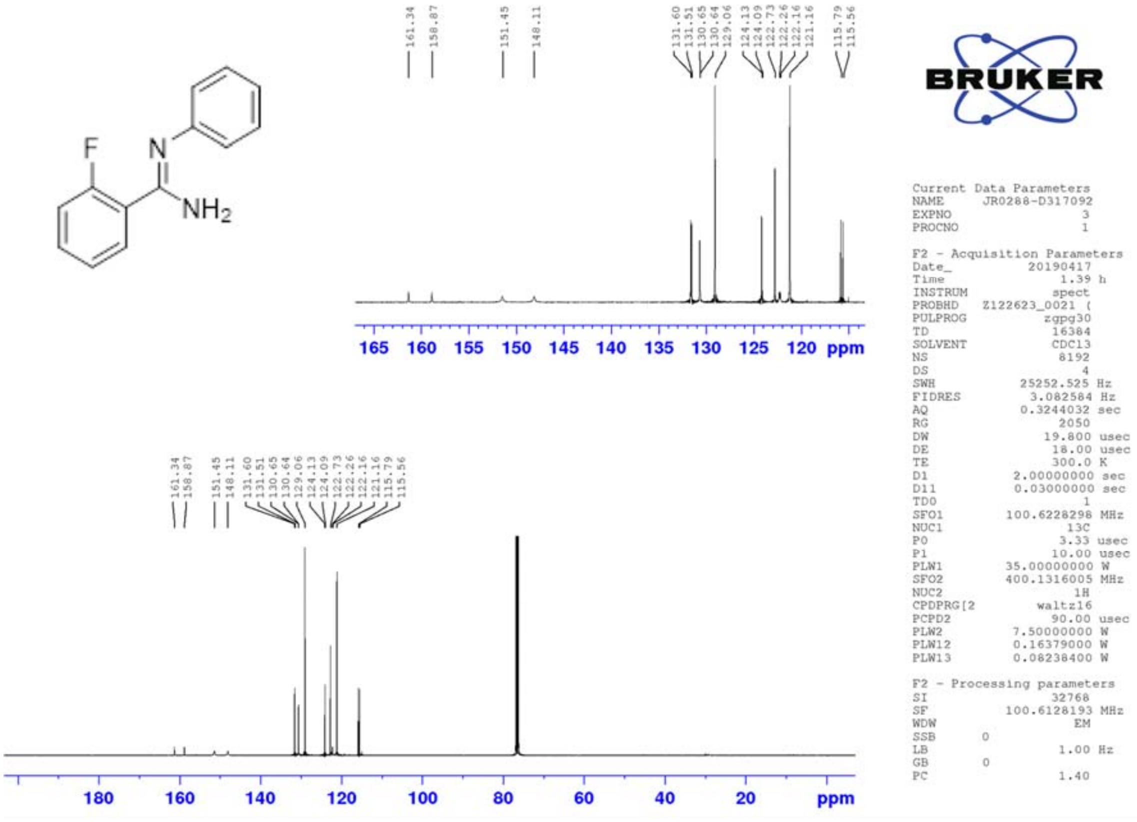

^{19}F NMR

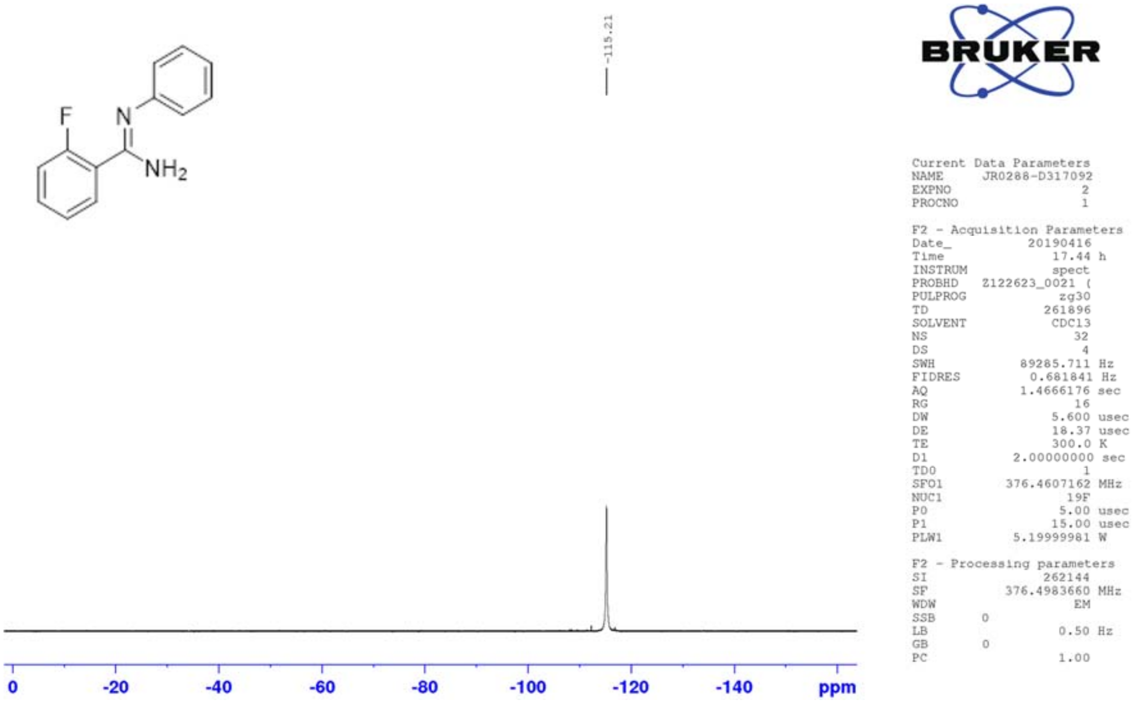

(*Z*)-3-bromo-*N*'-phenylbenzimidamide (**27**) - ^{1}H NMR

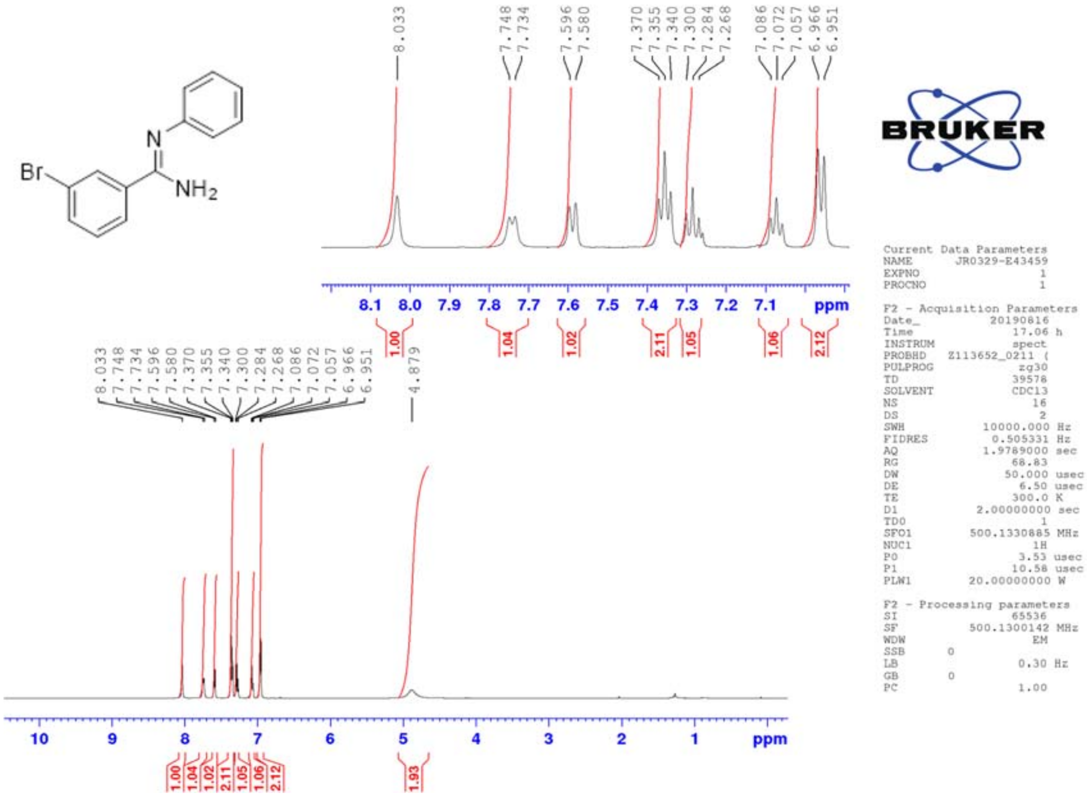

^{13}C NMR

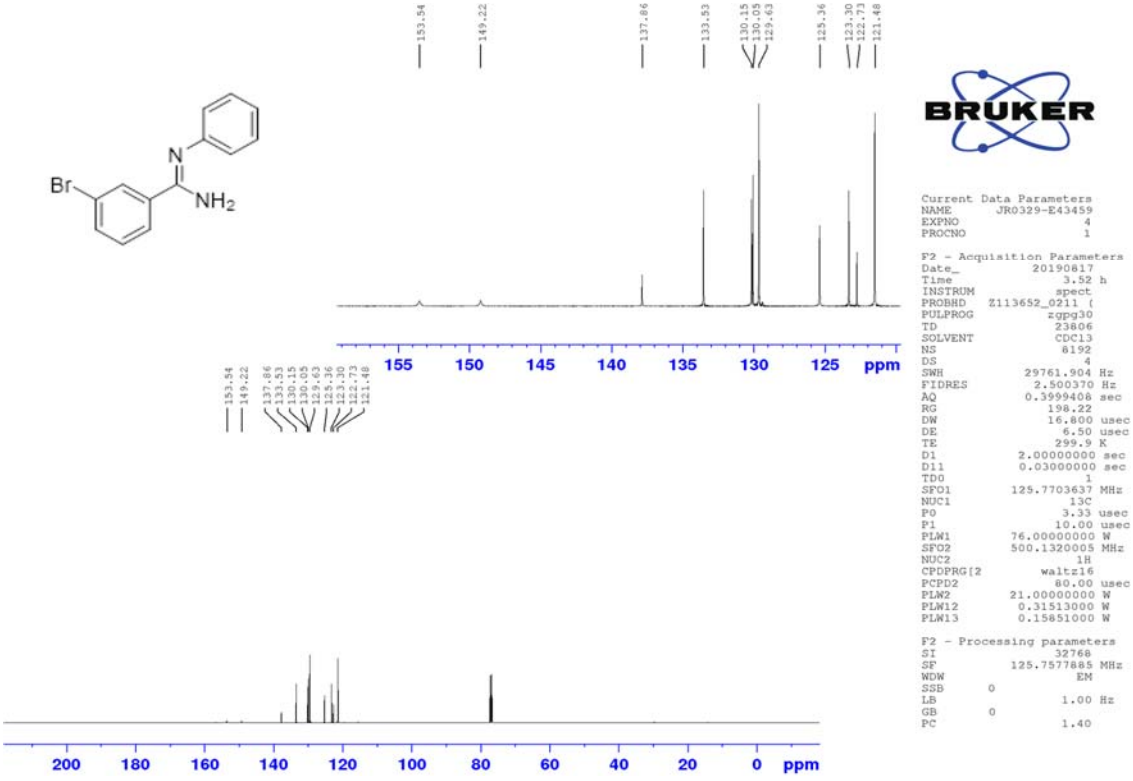

(*Z*)-4-methyl-*N'*-phenylbenzimidamide (**28**) - ^{1}H NMR

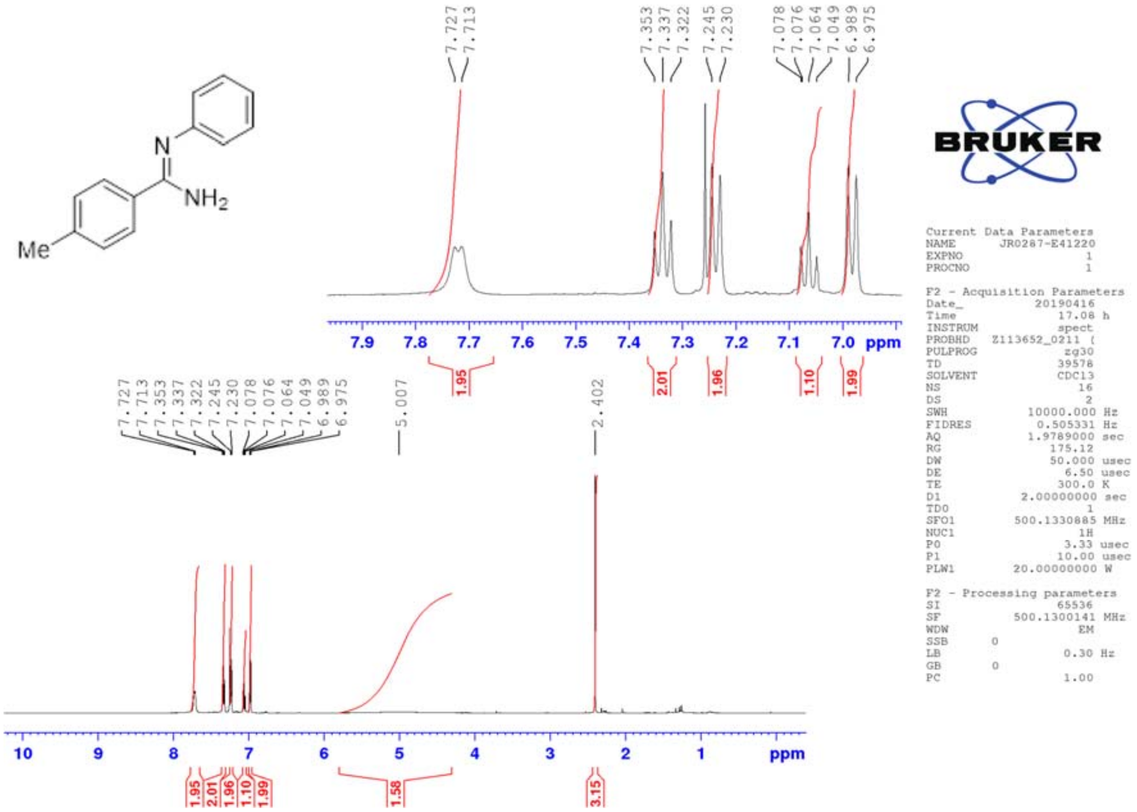

^{13}C NMR

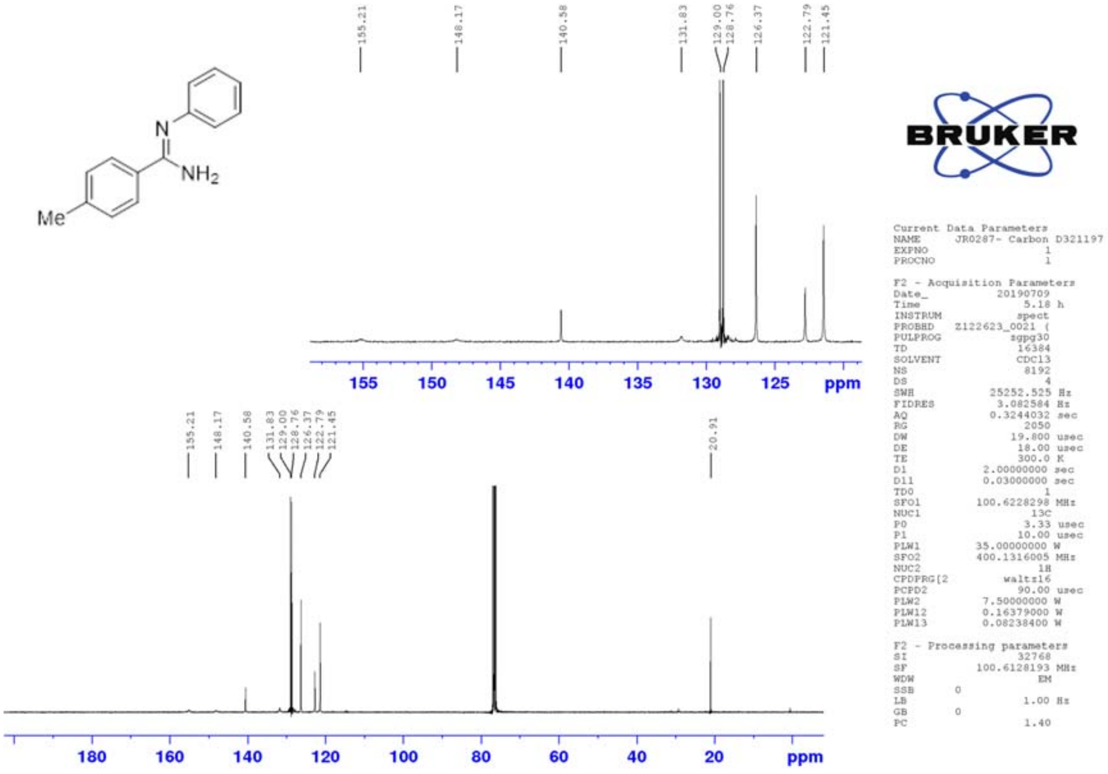

(*Z*)-3-cyano-*N*'-phenylbenzimidamide (**29**) - ^{1}H NMR

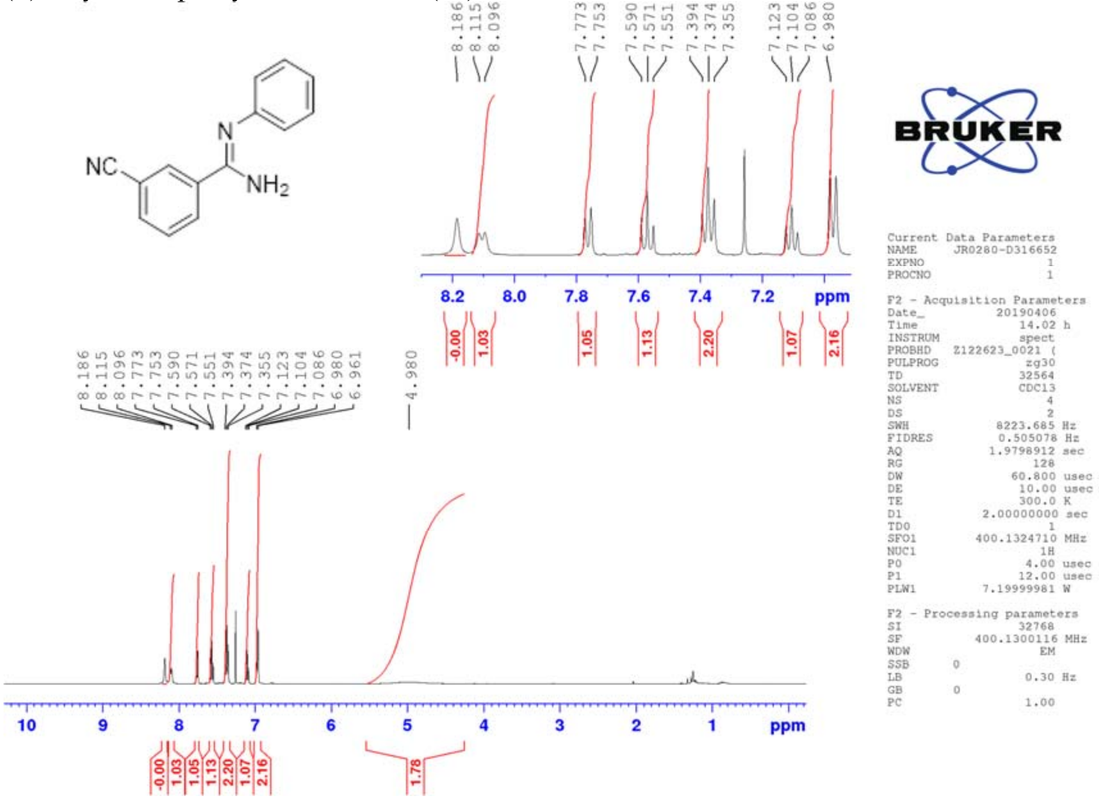

^{13}C NMR

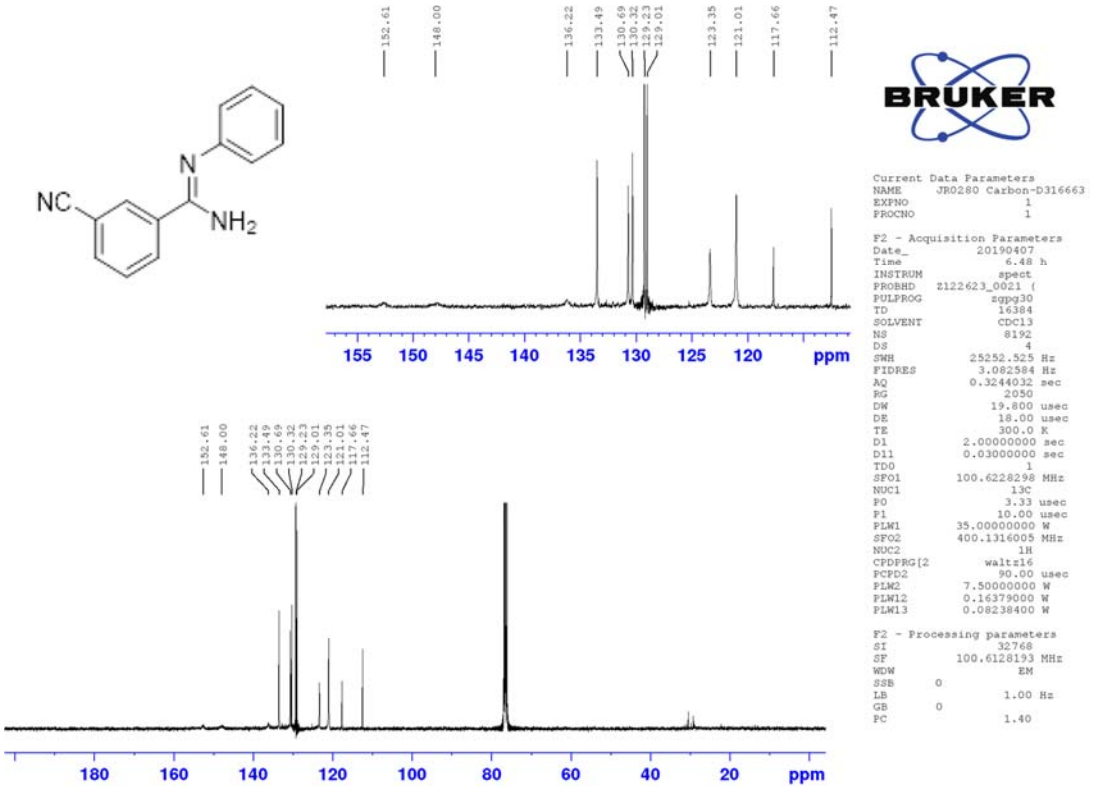

(*Z*)-4-((5-(4-carbamimidoylphenoxy)pentyl)oxy)-*N*'-phenylbenzimidamide (**30**) - ^{1}H NMR

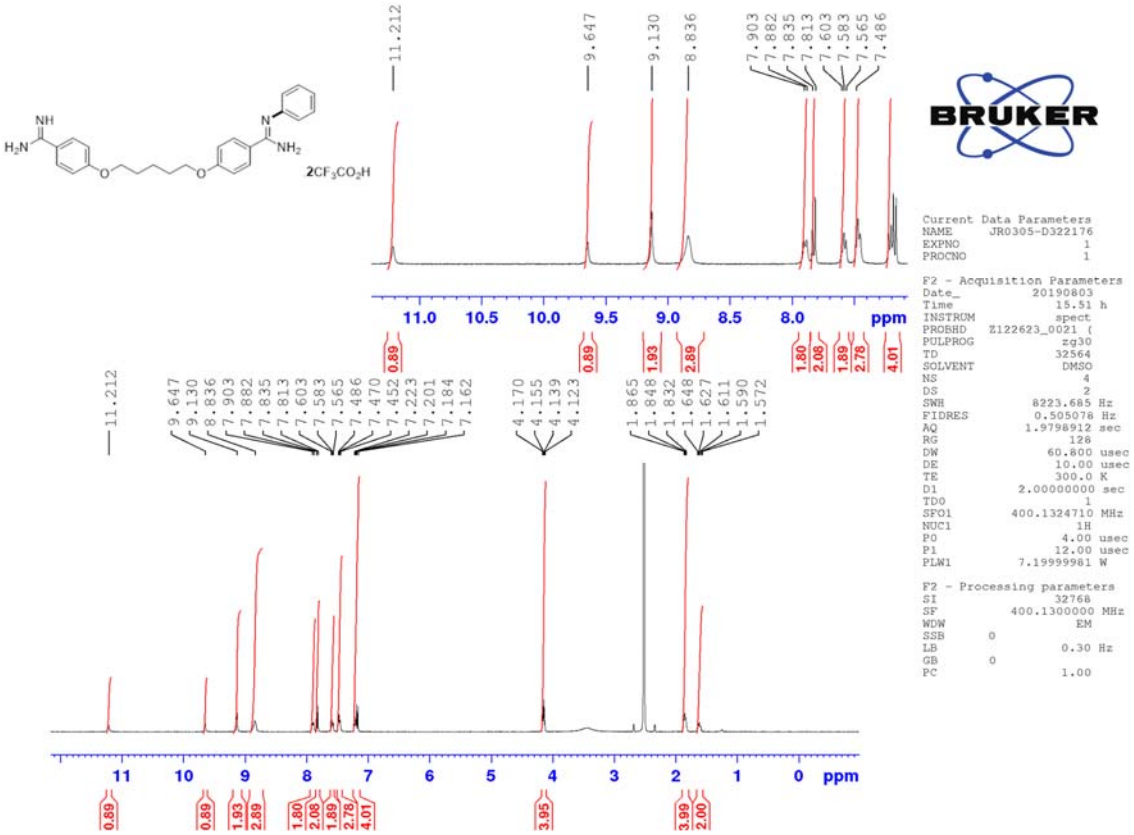

^{13}C NMR

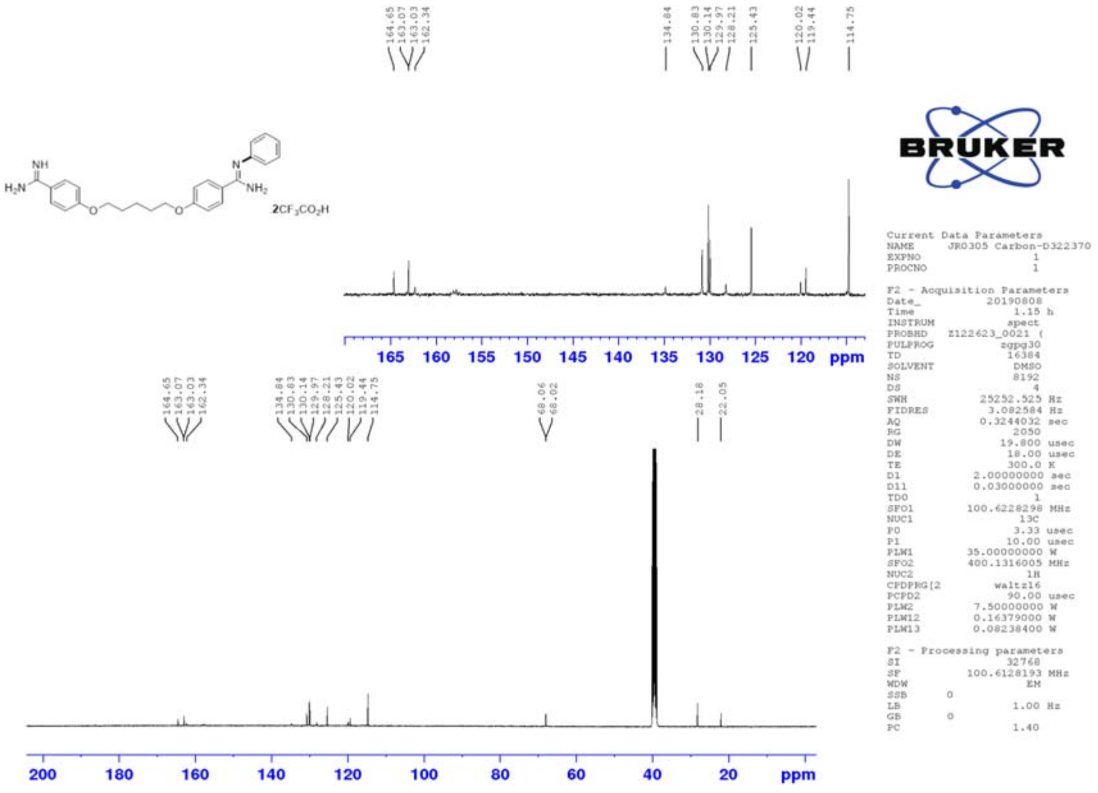

^{19}F NMR

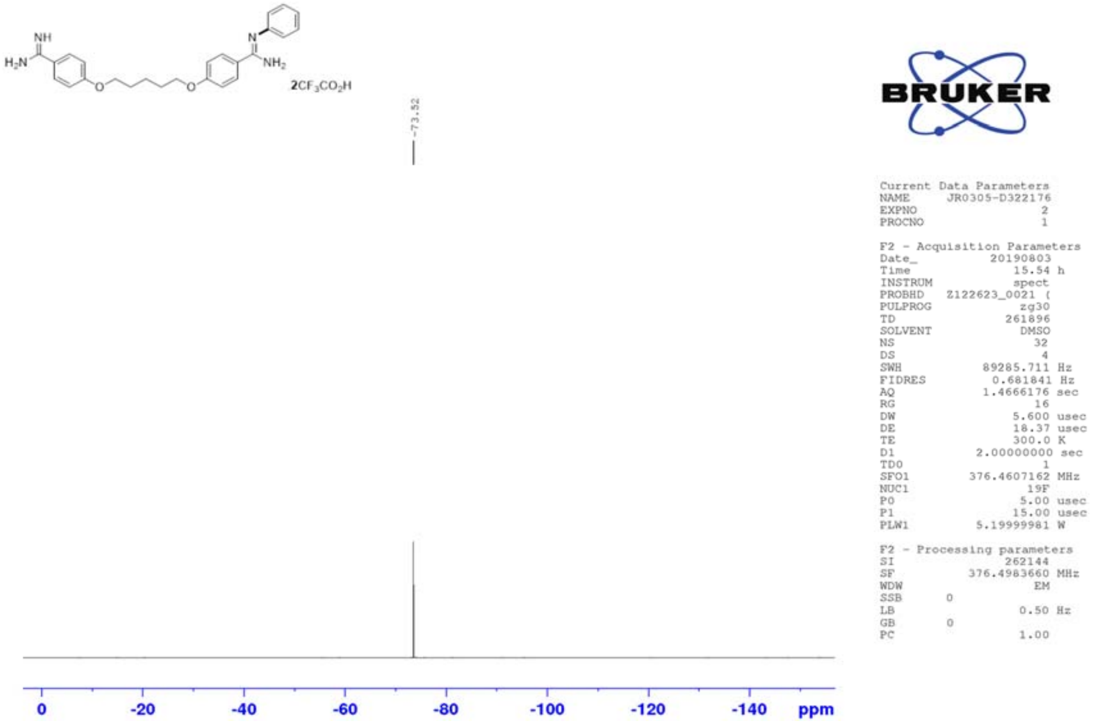

(Z)-*N'*-([1,1'-biphenyl]-4-yl)-4-((5-(4-carbamimidoylphenoxy)pentyl)oxy)benzimidamide (**31**) - ^{1}H NMR

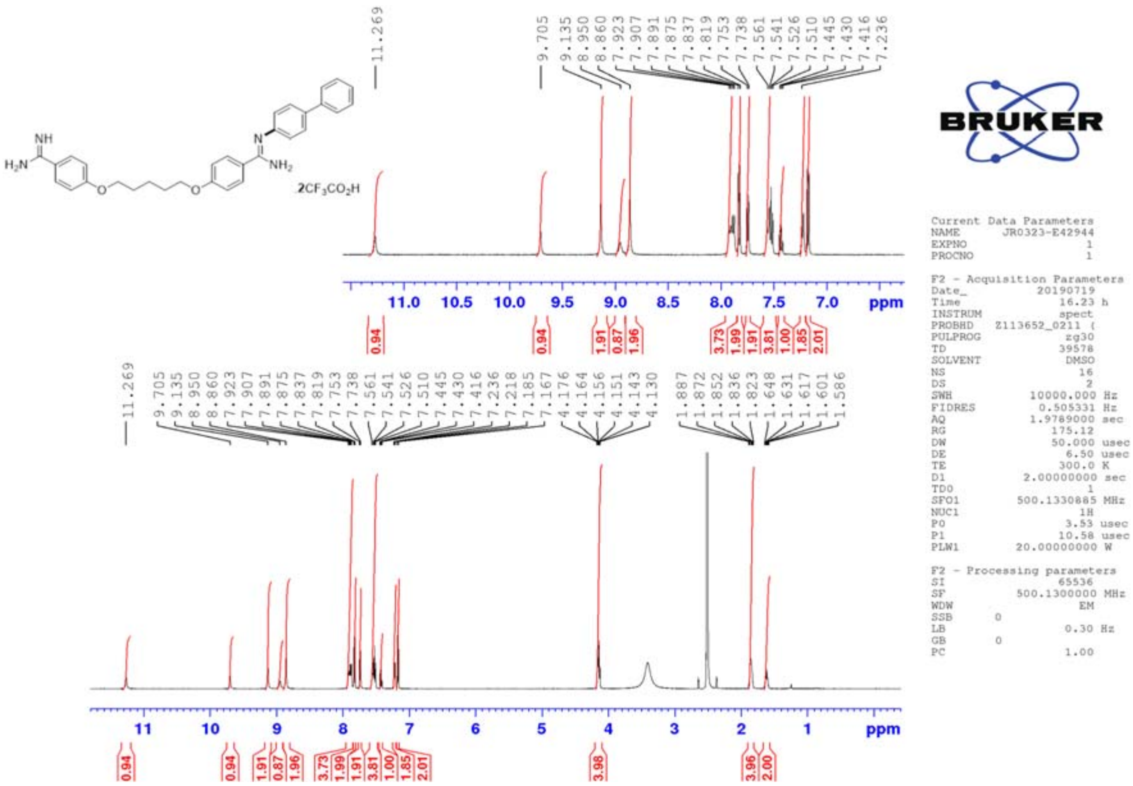

^{13}C NMR

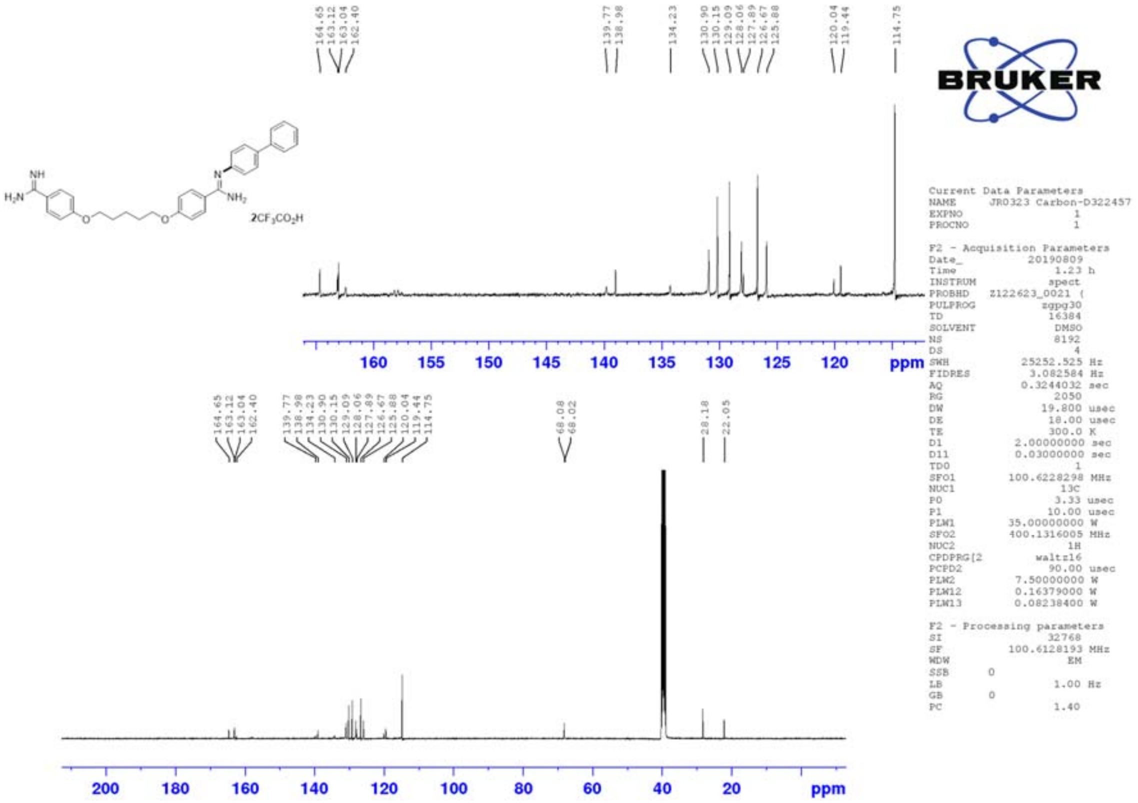

^{19}F NMR

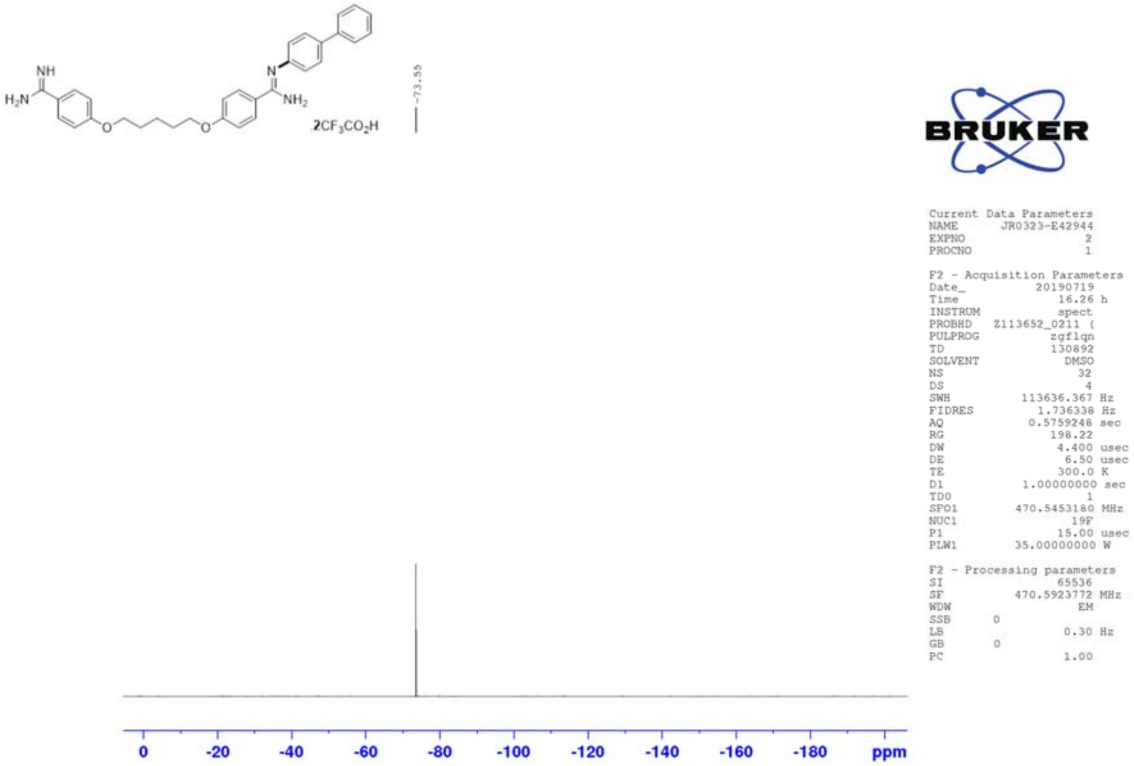

(*Z*)-4-((5-(4-carbamimidoylphenoxy)pentyl)oxy)-*N'*-(4-(trifluoromethyl)phenyl)benzimidamide (**32**) - ^{1}H NMR

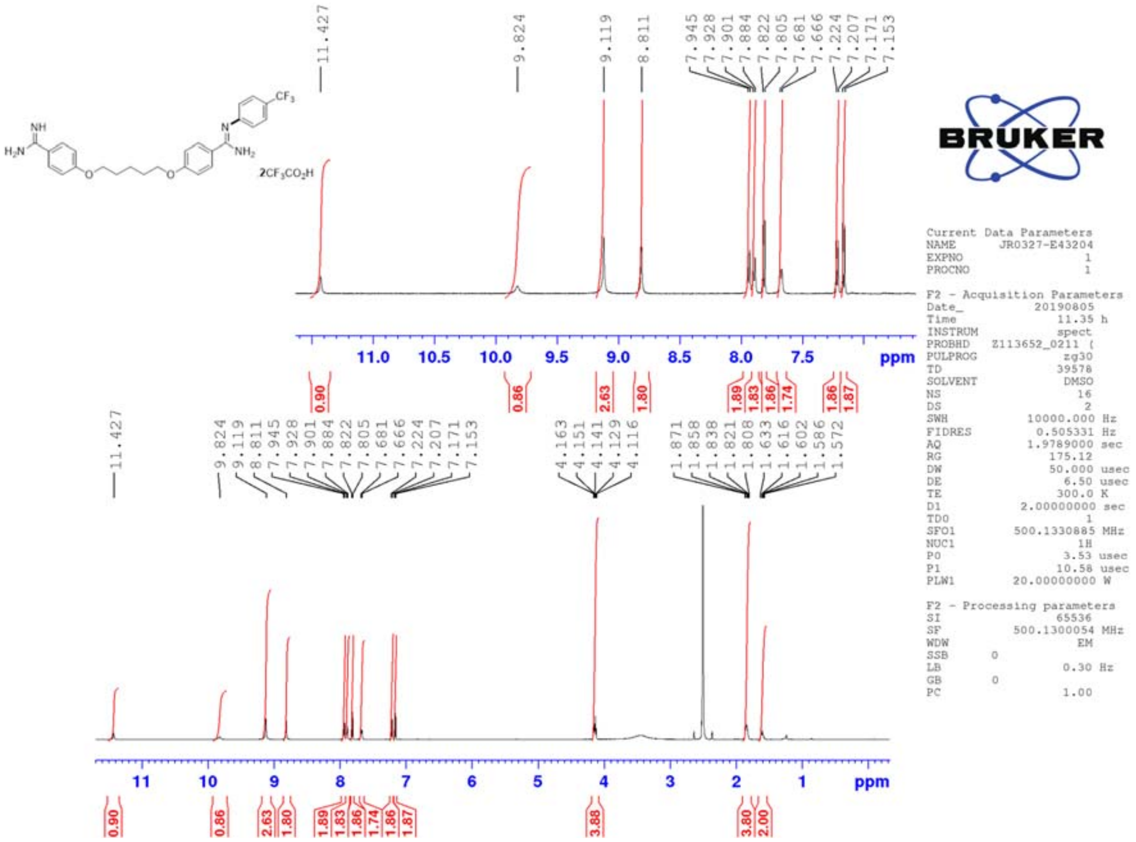

^{13}C NMR

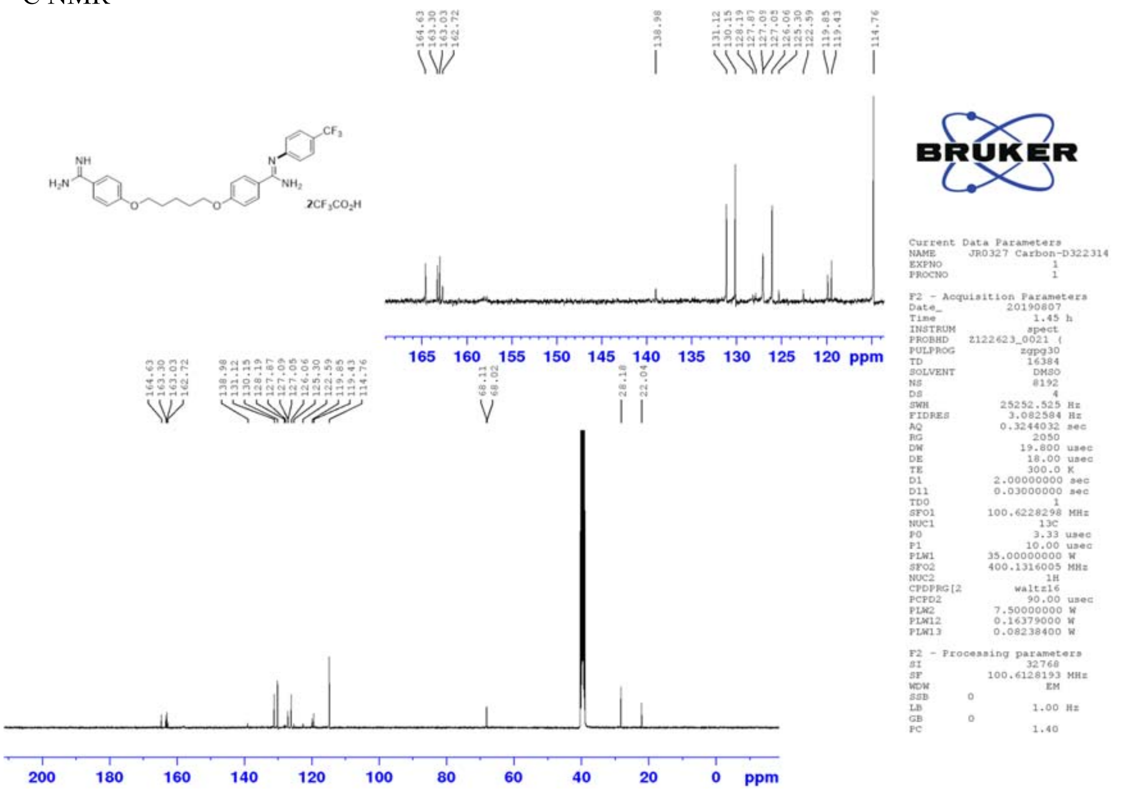

^{19}F NMR

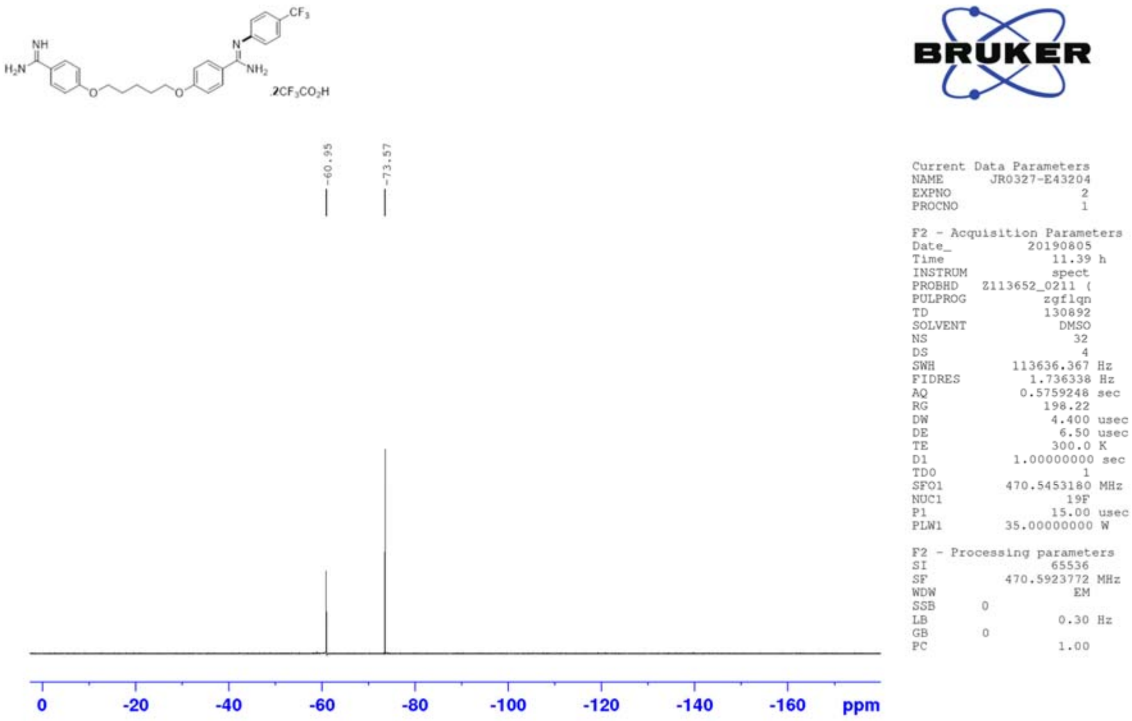

(*Z*)-4-((5-(4-carbamimidoylphenoxy)pentyl)oxy)-N'-(4-(dimethylamino)phenyl)benzimidamide (**33**) - ^{1}H NMR

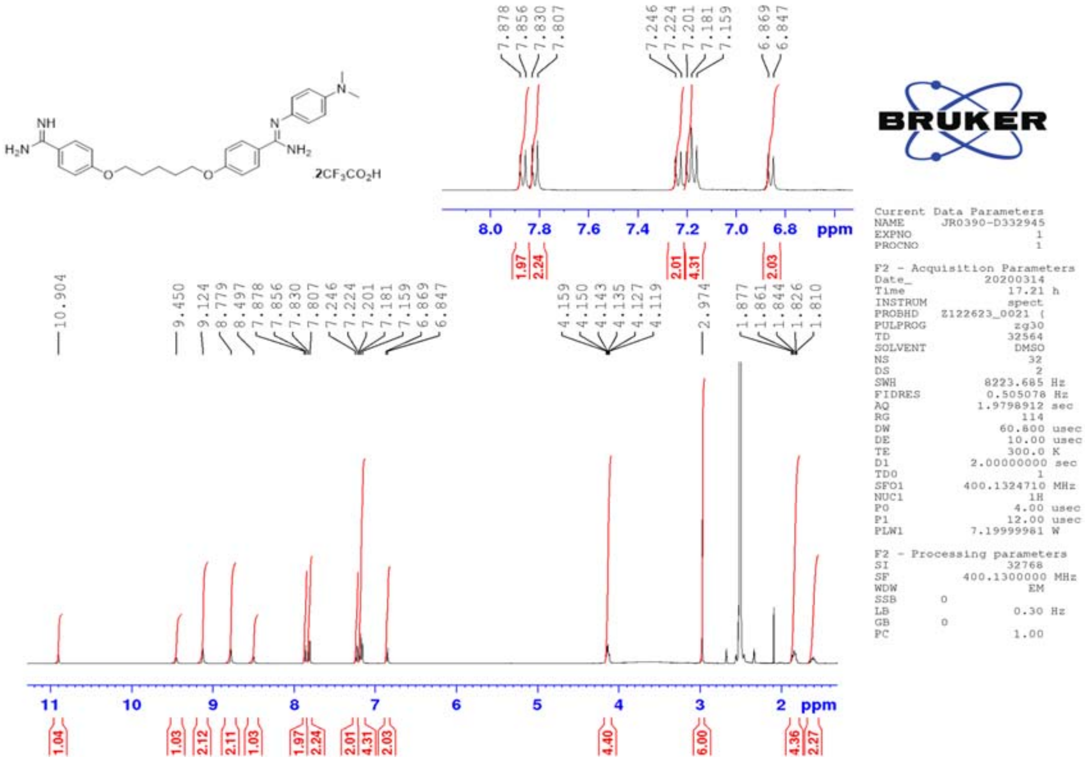

^{13}C NMR

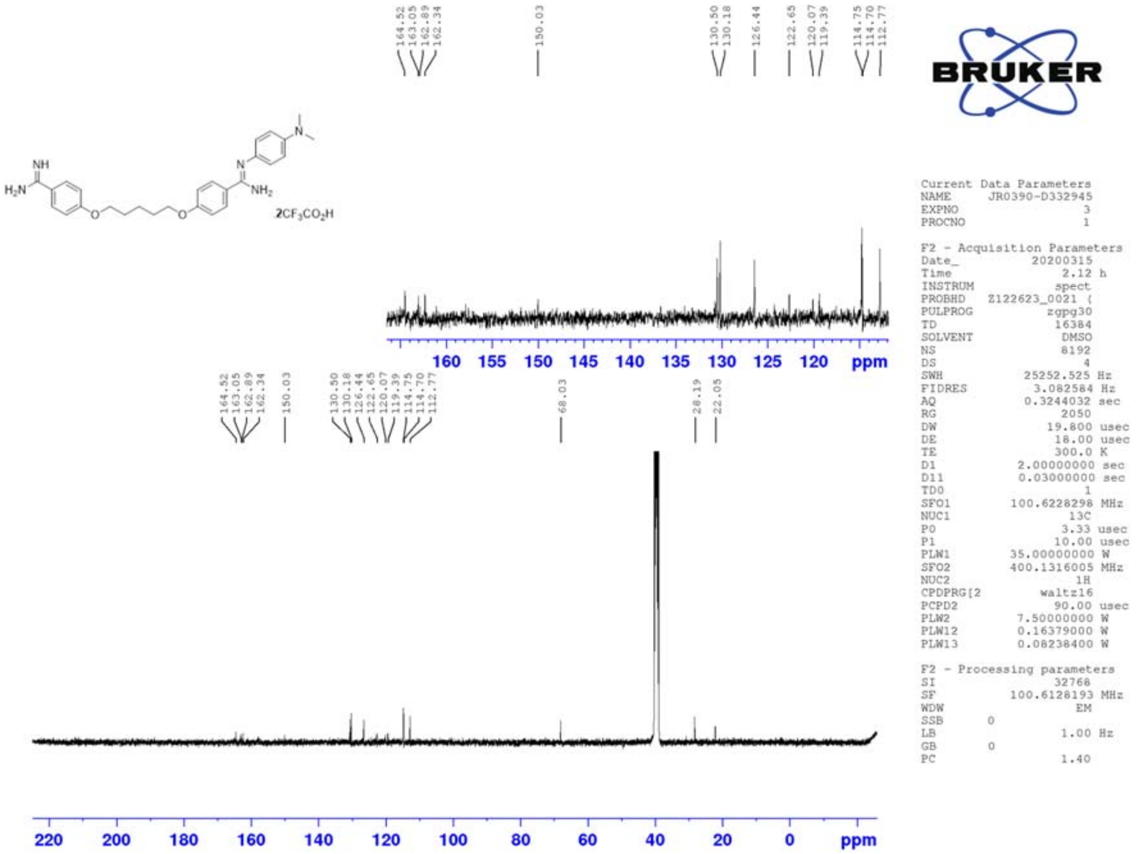

^{19}F NMR

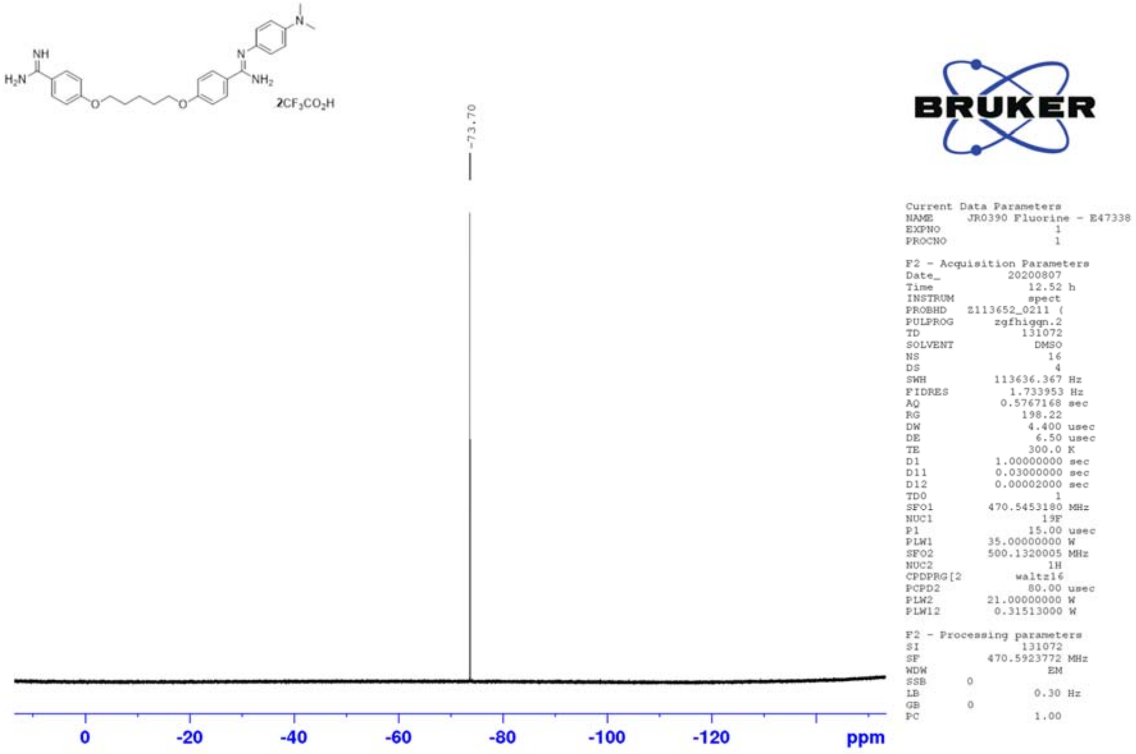

(*Z*)-4-((5-(4-carbamimidoylphenoxy)pentyl)oxy)-*N'*-(3-fluoro-4-methoxyphenyl)benzimidamide (**34**) - ^{1}H NMR

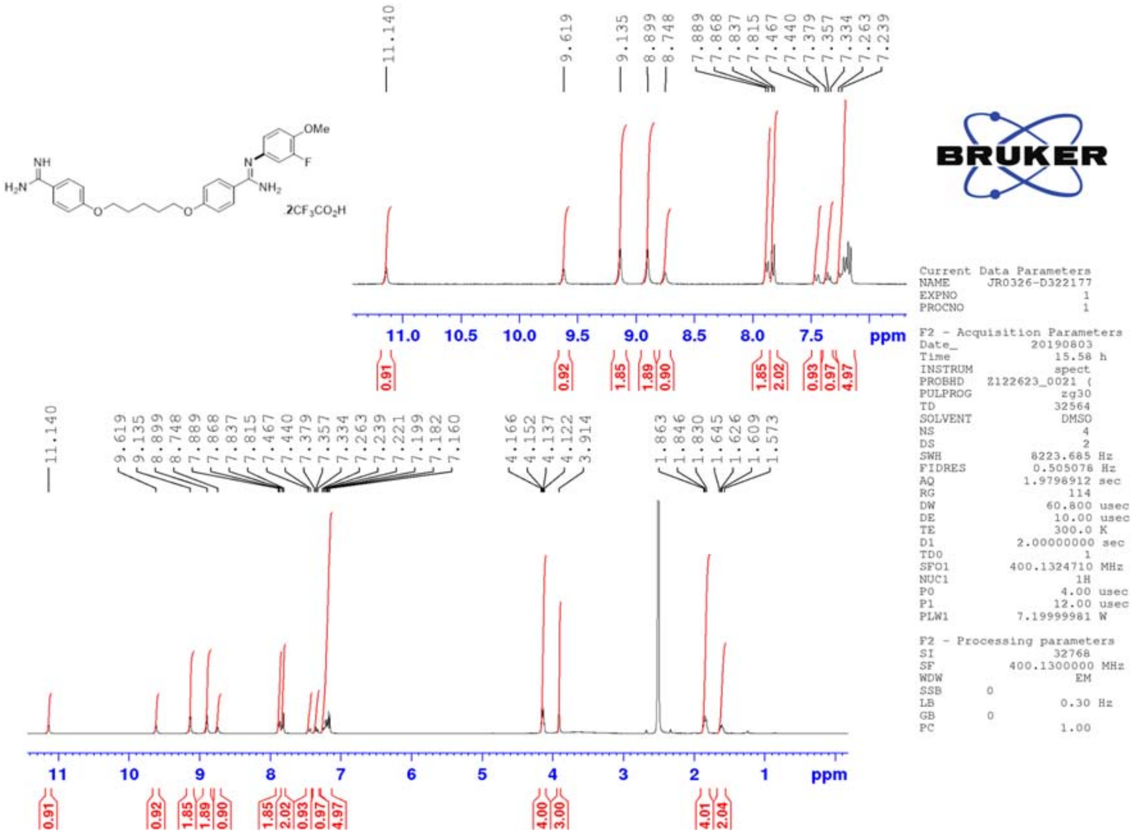

^{13}C NMR

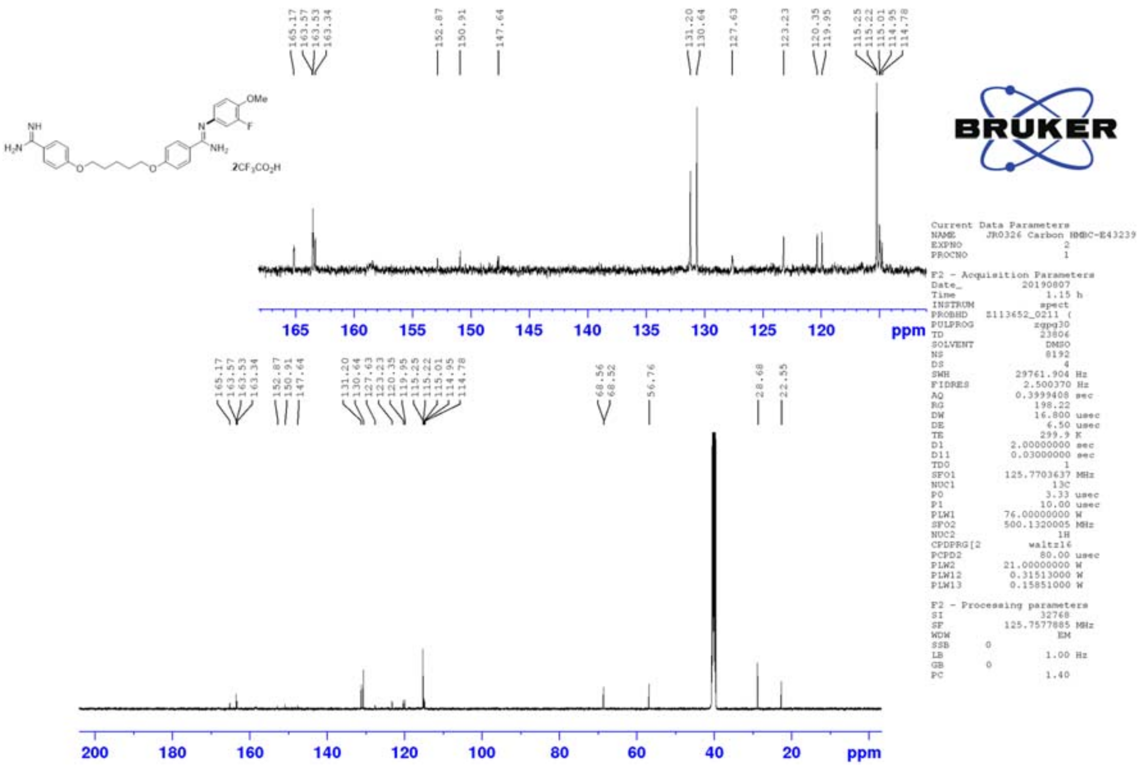

^{19}F NMR

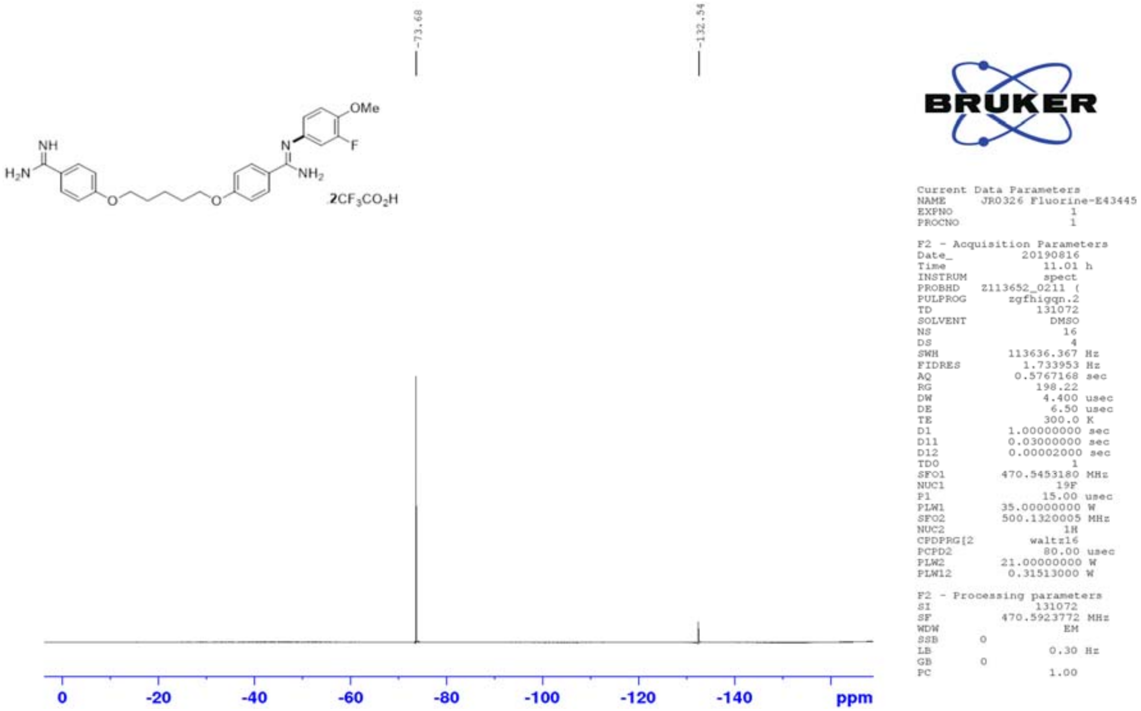

(*Z*)-4-((5-(4-carbamimidoylphenoxy)pentyl)oxy)-*N*'-(quinolin-3-yl)benzimidamide (**35**) - ^{1}H NMR

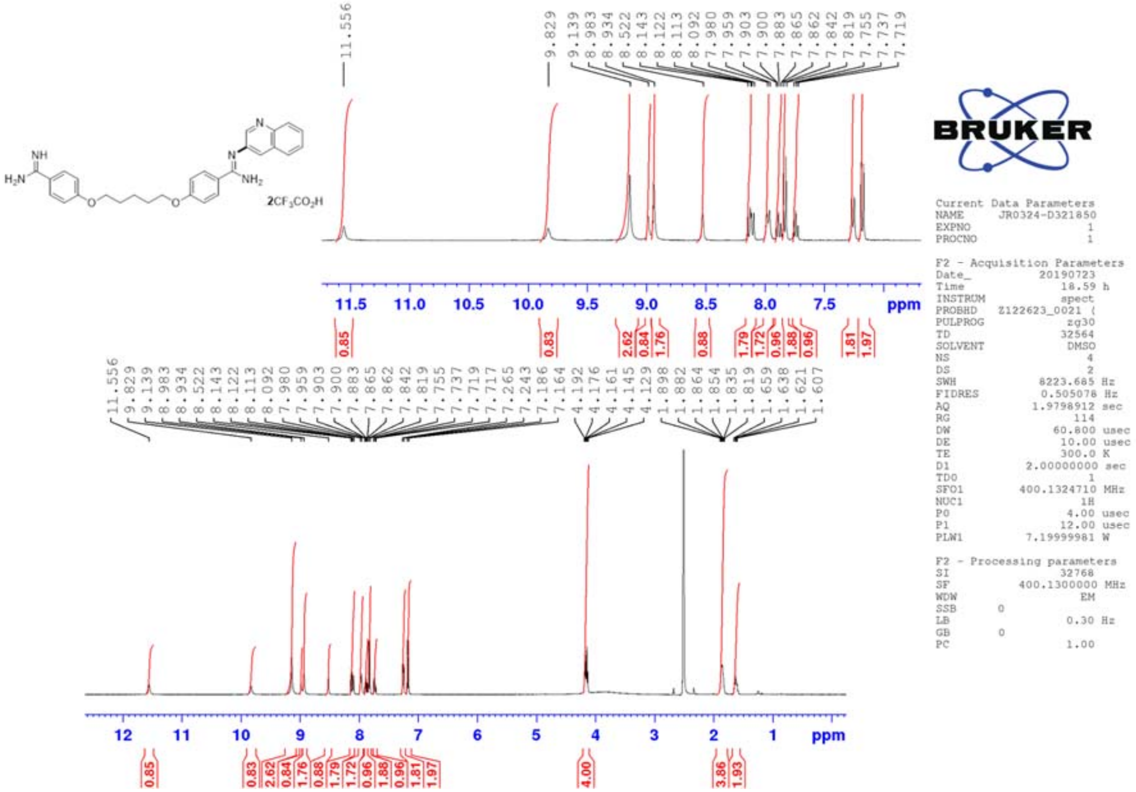

^{13}C NMR

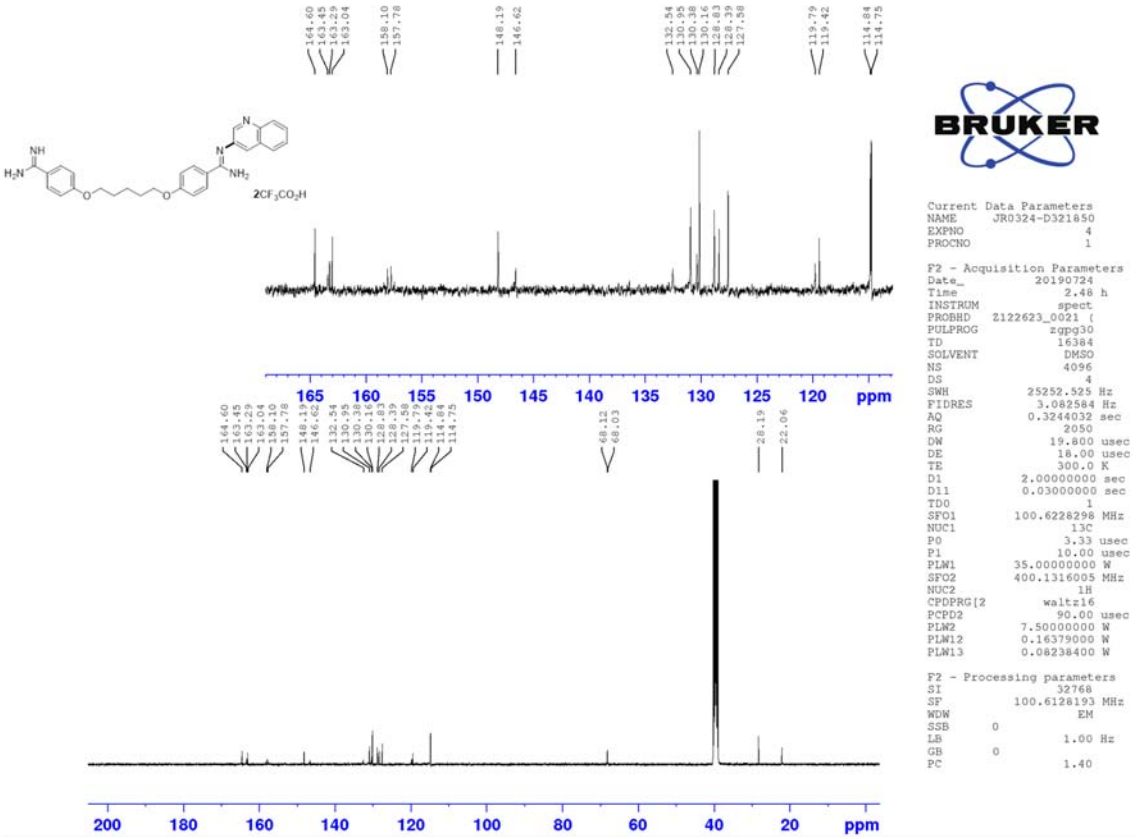

^{19}F NMR

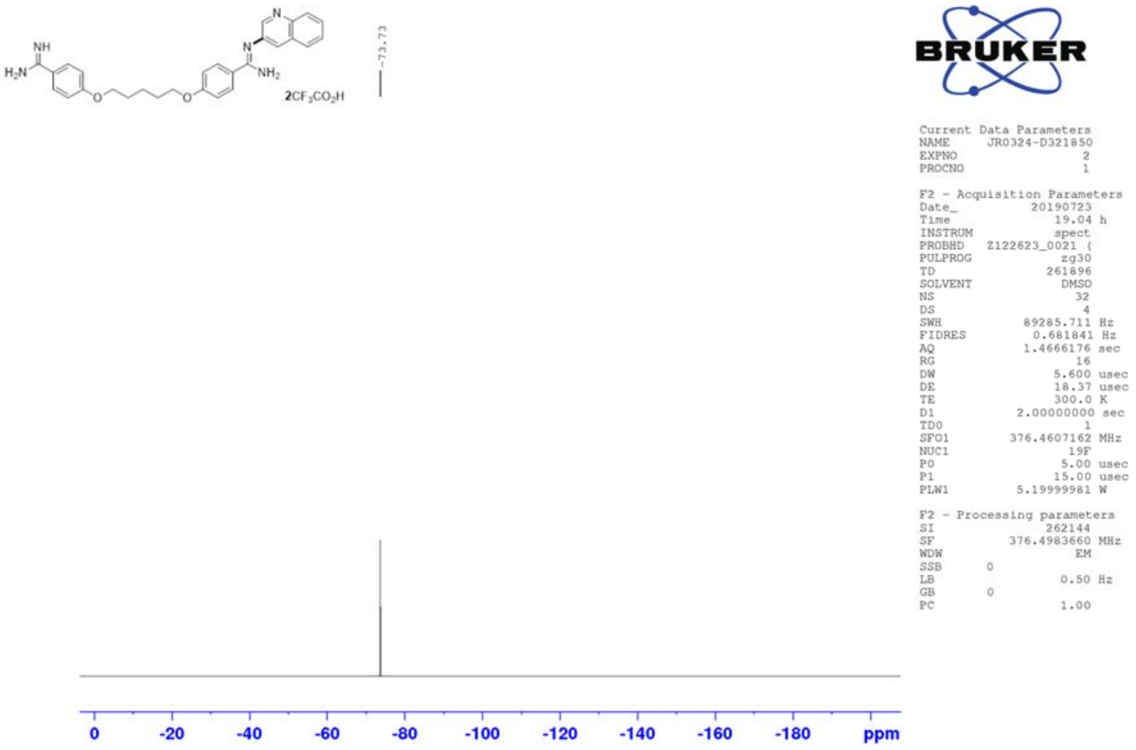

(*Z*)-4-((5-(4-carbamimidoylphenoxy)pentyl)oxy)-N'-(3-((5-(dimethylamino)naphthalene)-1-sulfonamido)phenyl)benzimidamidem (**36**) - ^{1}H NMR

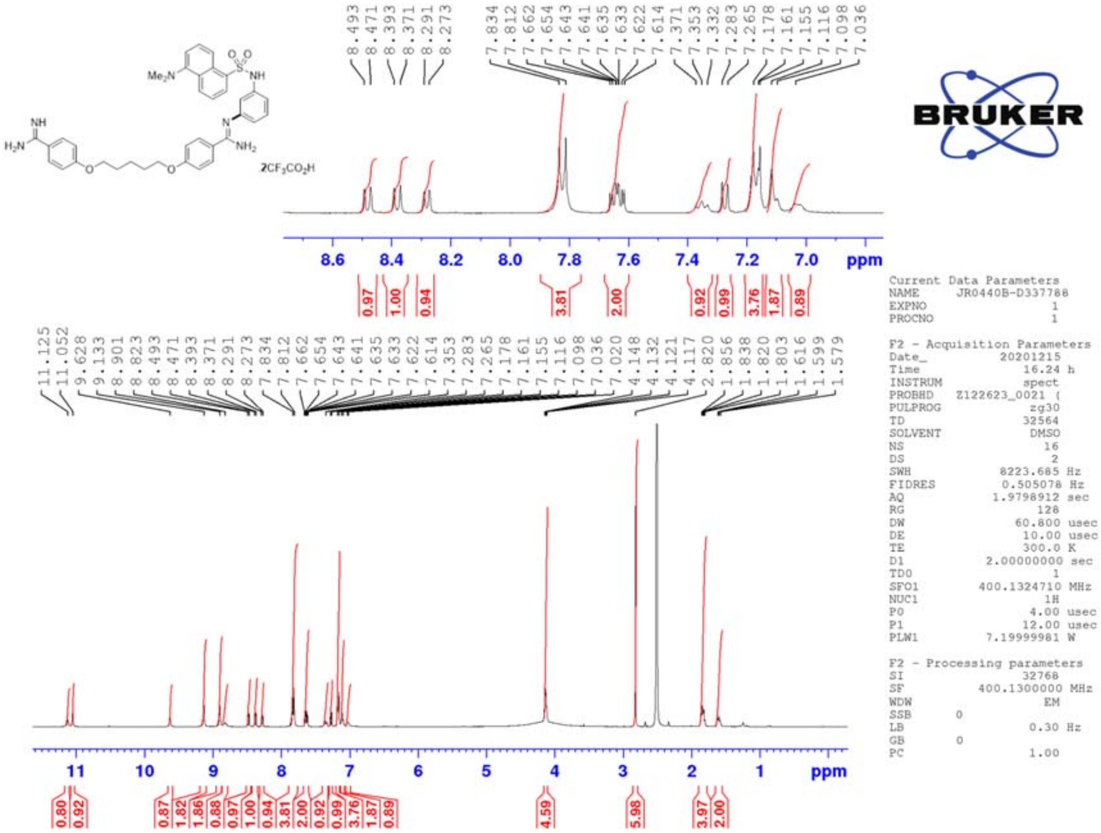

^{13}C NMR

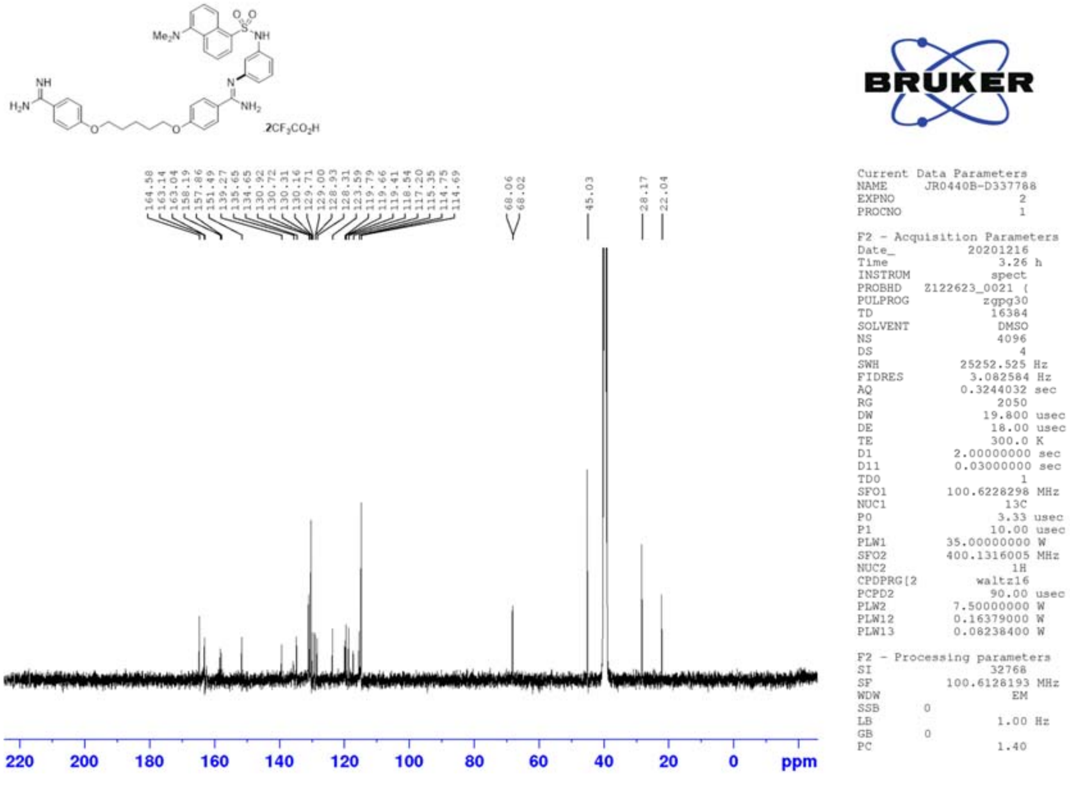

^{19}F NMR

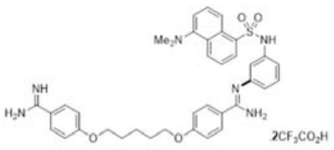

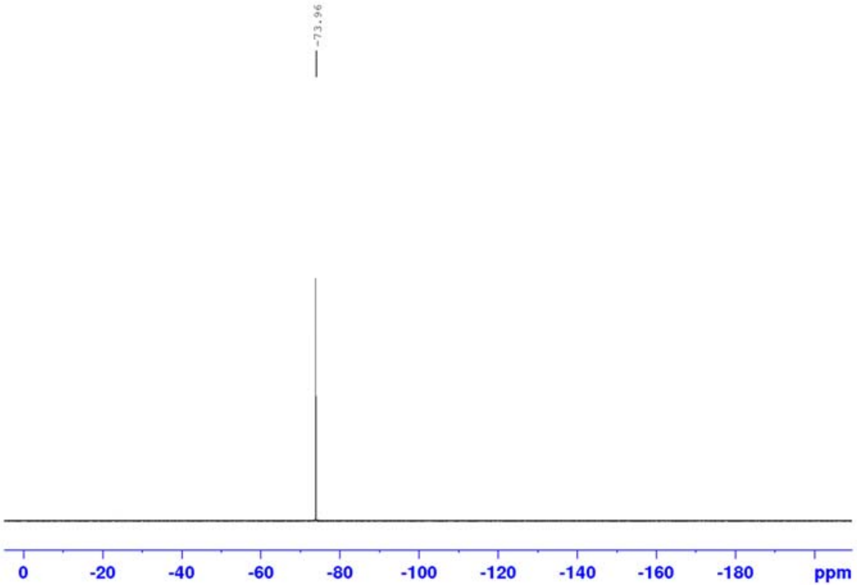


```
Current Data Parameters
NAME      JR0440-D337424
EXPNO                  2
PROCNO                 1

F2 - Acquisition Parameters
Date_           20201207
Time               18.16 h
INSTRUM            spect
PROBHD    Z122623_0021 (
PULPROG             zg30
TD                261896
SOLVENT             DMSO
NS                    32
DS                     4
SWH            89285.711 Hz
FIDRES          0.681841 Hz
AQ             1.4666176 sec
RG                  20.2
DW                 5.600 usec
DE                 18.37 usec
TE                 300.0 K
D1            2.00000000 sec
TD0                    1
SFO1         376.4607162 MHz
NUC1                 19F
P0                  5.00 usec
P1                 15.00 usec
PLW1          5.19999981 W

F2 - Processing parameters
SI                262144
SF           376.4983660 MHz
WDW                   EM
SSB       0
LB                  0.50 Hz
GB        0
PC                  1.00
```

Crystallographic data tables

Complex **4**

Table S8. Crystal data and structure refinement for complex **4**.

Empirical formula	C15 H16 Cu N4 O3	
Formula weight	363.86	
Temperature	123(2) K	
Wavelength	1.54184 Å	
Crystal system	Orthorhombic	
Space group	P 2_1 2_1 2_1	
Unit cell dimensions	a = 9.9702(5) Å	α= 90°.
	b = 11.3577(4) Å	β= 90°.
	c = 13.1156(5) Å	γ = 90°.
Volume	1485.19(11) $Å^3$	
Z	4	
Density (calculated)	1.627 Mg/m^3	
Absorption coefficient	2.271 mm^{-1}	
F(000)	748	
Crystal size	0.40 x 0.08 x 0.03 mm^3	
Theta range for data collection	5.151 to 73.200°.	
Index ranges	-12<=h<=11, -7<=k<=13, -15<=l<=16	
Reflections collected	5091	
Independent reflections	2919 [R(int) = 0.0301]	
Completeness to theta = 70.000°	100.0 %	
Absorption correction	Semi-empirical from equivalents	
Max. and min. transmission	1.00000 and 0.22234	
Refinement method	Full-matrix least-squares on F^2	
Data / restraints / parameters	2919 / 4 / 230	
Goodness-of-fit on F^2	1.030	
Final R indices [I>2sigma(I)]	R1 = 0.0337, wR2 = 0.0886	
R indices (all data)	R1 = 0.0360, wR2 = 0.0908	
Absolute structure parameter	-0.05(2)	
Extinction coefficient	n/a	
Largest diff. peak and hole	0.471 and -0.286 e.$Å^{-3}$	

Table S9. Atomic coordinates (x 10^4) and equivalent isotropic displacement parameters (Å^{2}x 10^3) for complex **4**. U(eq) is defined as one third of the trace of the orthogonalized U^{ij} tensor.

	x	y	z	U(eq)
Cu(1)	1414(1)	85(1)	3900(1)	15(1)
O(1)	1043(3)	1082(2)	2701(2)	16(1)
O(2)	1353(3)	-815(2)	2613(2)	17(1)
O(3)	903(3)	187(2)	1176(2)	19(1)
N(1)	1264(4)	1237(2)	4984(2)	18(1)
N(2)	163(4)	2737(3)	4191(3)	21(1)
N(3)	1928(3)	-1217(3)	4780(2)	16(1)
N(4)	618(4)	-2638(3)	4024(3)	24(1)
C(1)	659(4)	2263(3)	5031(3)	15(1)
C(2)	481(3)	2899(3)	6019(3)	15(1)
C(3)	-276(4)	3937(3)	6063(3)	20(1)
C(4)	-420(4)	4547(4)	6973(3)	23(1)
C(5)	177(4)	4121(4)	7853(3)	24(1)
C(6)	928(4)	3100(3)	7816(3)	24(1)
C(7)	1086(4)	2501(3)	6902(3)	20(1)
C(8)	1517(4)	-2306(3)	4715(3)	15(1)
C(9)	2058(4)	-3256(3)	5395(3)	18(1)
C(10)	2296(4)	-3030(4)	6422(3)	21(1)
C(11)	2817(4)	-3913(4)	7029(4)	29(1)
C(12)	3132(4)	-4998(4)	6630(4)	31(1)
C(13)	2909(4)	-5217(4)	5598(4)	29(1)
C(14)	2360(4)	-4345(3)	4991(3)	22(1)
C(15)	1090(3)	154(3)	2117(3)	16(1)

Table S10. Bond lengths [Å] and angles [°] for complex **4**.

Cu(1)-N(1)	1.938(3)
Cu(1)-N(3)	1.945(3)
Cu(1)-O(1)	1.973(3)
Cu(1)-O(2)	1.974(2)
Cu(1)-C(15)	2.362(4)
O(1)-C(15)	1.304(4)
O(2)-C(15)	1.305(4)
O(3)-C(15)	1.249(4)
N(1)-C(1)	1.314(5)
N(1)-H(1N)	0.88(5)
N(2)-C(1)	1.322(5)
N(2)-H(2N)	0.874(14)
N(2)-H(3N)	0.884(14)
N(3)-C(8)	1.306(5)
N(3)-H(4N)	0.80(5)
N(4)-C(8)	1.329(5)
N(4)-H(5N)	0.869(14)
N(4)-H(6N)	0.874(14)
C(1)-C(2)	1.494(5)
C(2)-C(7)	1.382(5)
C(2)-C(3)	1.402(5)
C(3)-C(4)	1.388(5)
C(3)-H(3)	0.9500
C(4)-C(5)	1.386(6)
C(4)-H(4)	0.9500
C(5)-C(6)	1.382(6)
C(5)-H(5)	0.9500
C(6)-C(7)	1.387(5)
C(6)-H(6)	0.9500
C(7)-H(7)	0.9500
C(8)-C(9)	1.500(5)
C(9)-C(14)	1.379(5)
C(9)-C(10)	1.391(6)
C(10)-C(11)	1.382(6)
C(10)-H(10)	0.9500
C(11)-C(12)	1.376(7)

C(11)-H(11)	0.9500
C(12)-C(13)	1.393(7)
C(12)-H(12)	0.9500
C(13)-C(14)	1.384(6)
C(13)-H(13)	0.9500
C(14)-H(14)	0.9500

N(1)-Cu(1)-N(3)	95.64(13)
N(1)-Cu(1)-O(1)	100.52(11)
N(3)-Cu(1)-O(1)	163.55(12)
N(1)-Cu(1)-O(2)	167.01(12)
N(3)-Cu(1)-O(2)	97.01(12)
O(1)-Cu(1)-O(2)	67.04(10)
N(1)-Cu(1)-C(15)	133.90(13)
N(3)-Cu(1)-C(15)	130.46(13)
O(1)-Cu(1)-C(15)	33.51(11)
O(2)-Cu(1)-C(15)	33.53(11)
C(15)-O(1)-Cu(1)	89.8(2)
C(15)-O(2)-Cu(1)	89.8(2)
C(1)-N(1)-Cu(1)	132.0(3)
C(1)-N(1)-H(1N)	116(3)
Cu(1)-N(1)-H(1N)	112(3)
C(1)-N(2)-H(2N)	119(4)
C(1)-N(2)-H(3N)	124(4)
H(2N)-N(2)-H(3N)	117(5)
C(8)-N(3)-Cu(1)	126.8(3)
C(8)-N(3)-H(4N)	114(3)
Cu(1)-N(3)-H(4N)	118(3)
C(8)-N(4)-H(5N)	124(3)
C(8)-N(4)-H(6N)	125(4)
H(5N)-N(4)-H(6N)	109(4)
N(1)-C(1)-N(2)	119.6(4)
N(1)-C(1)-C(2)	121.6(3)
N(2)-C(1)-C(2)	118.8(3)
C(7)-C(2)-C(3)	118.4(4)
C(7)-C(2)-C(1)	121.1(3)
C(3)-C(2)-C(1)	120.4(4)
C(4)-C(3)-C(2)	120.8(4)

C(4)-C(3)-H(3)	119.6
C(2)-C(3)-H(3)	119.6
C(5)-C(4)-C(3)	119.9(4)
C(5)-C(4)-H(4)	120.1
C(3)-C(4)-H(4)	120.1
C(6)-C(5)-C(4)	119.7(4)
C(6)-C(5)-H(5)	120.1
C(4)-C(5)-H(5)	120.1
C(5)-C(6)-C(7)	120.3(4)
C(5)-C(6)-H(6)	119.9
C(7)-C(6)-H(6)	119.9
C(2)-C(7)-C(6)	121.0(4)
C(2)-C(7)-H(7)	119.5
C(6)-C(7)-H(7)	119.5
N(3)-C(8)-N(4)	121.6(3)
N(3)-C(8)-C(9)	122.0(3)
N(4)-C(8)-C(9)	116.4(3)
C(14)-C(9)-C(10)	120.1(4)
C(14)-C(9)-C(8)	119.7(4)
C(10)-C(9)-C(8)	120.3(4)
C(11)-C(10)-C(9)	119.2(4)
C(11)-C(10)-H(10)	120.4
C(9)-C(10)-H(10)	120.4
C(12)-C(11)-C(10)	121.1(4)
C(12)-C(11)-H(11)	119.5
C(10)-C(11)-H(11)	119.5
C(11)-C(12)-C(13)	119.5(4)
C(11)-C(12)-H(12)	120.2
C(13)-C(12)-H(12)	120.2
C(14)-C(13)-C(12)	119.7(4)
C(14)-C(13)-H(13)	120.2
C(12)-C(13)-H(13)	120.2
C(9)-C(14)-C(13)	120.4(4)
C(9)-C(14)-H(14)	119.8
C(13)-C(14)-H(14)	119.8
O(3)-C(15)-O(1)	123.4(3)
O(3)-C(15)-O(2)	123.2(3)
O(1)-C(15)-O(2)	113.3(3)

O(3)-C(15)-Cu(1)	179.3(3)
O(1)-C(15)-Cu(1)	56.65(17)
O(2)-C(15)-Cu(1)	56.69(17)

Symmetry transformations used to generate equivalent atoms:

Table S11. Anisotropic displacement parameters (Å^{2}x 10^3) for complex **4**. The anisotropic displacement factor exponent takes the form: $-2\pi^2[h^2a^{*2}U^{11} + ... + 2 h k a^* b^* U^{12}]$

	U^{11}	U^{22}	U^{33}	U^{23}	U^{13}	U^{12}
Cu(1)	20(1)	10(1)	14(1)	0(1)	-2(1)	1(1)
O(1)	24(1)	11(1)	15(1)	1(1)	-1(1)	1(1)
O(2)	25(1)	10(1)	16(1)	-2(1)	-1(1)	2(1)
O(3)	27(1)	15(1)	15(1)	0(1)	1(1)	-3(1)
N(1)	27(2)	12(1)	13(1)	-2(1)	-1(2)	3(1)
N(2)	34(2)	14(2)	15(2)	-1(1)	0(1)	7(1)
N(3)	22(2)	12(1)	16(2)	3(1)	-6(1)	1(1)
N(4)	30(2)	16(2)	26(2)	7(1)	-10(2)	-6(1)
C(1)	18(2)	10(2)	17(2)	1(1)	-1(2)	-4(1)
C(2)	17(2)	12(2)	18(2)	0(1)	2(2)	-4(1)
C(3)	19(2)	18(2)	23(2)	1(2)	-3(2)	5(1)
C(4)	19(2)	24(2)	25(2)	-8(2)	3(2)	5(2)
C(5)	25(2)	26(2)	21(2)	-7(2)	3(2)	-5(2)
C(6)	33(2)	24(2)	16(2)	3(2)	-3(2)	-4(2)
C(7)	28(2)	12(2)	21(2)	0(1)	1(2)	0(1)
C(8)	17(2)	12(2)	16(2)	0(1)	0(2)	3(1)
C(9)	14(2)	16(2)	22(2)	6(2)	-3(2)	-3(1)
C(10)	18(2)	18(2)	26(2)	3(1)	-3(2)	-5(1)
C(11)	23(2)	35(2)	29(2)	12(2)	-10(2)	-9(2)
C(12)	17(2)	28(2)	47(2)	23(2)	-7(2)	-4(2)
C(13)	20(2)	15(2)	53(3)	9(2)	4(2)	2(1)
C(14)	22(2)	16(2)	30(2)	4(2)	6(2)	0(1)
C(15)	15(1)	12(2)	20(2)	1(1)	2(1)	-2(1)

Table S12. Hydrogen coordinates (x 10^4) and isotropic displacement parameters ($Å^2$x 10^3) for complex **4**.

	x	y	z	U(eq)
H(1N)	1650(50)	1000(40)	5560(30)	21
H(4N)	2530(50)	-1120(40)	5170(40)	20
H(3)	-696	4227	5463	24
H(4)	-926	5255	6993	27
H(5)	71	4530	8480	29
H(6)	1338	2806	8418	29
H(7)	1617	1807	6883	24
H(10)	2102	-2278	6702	25
H(11)	2960	-3768	7734	35
H(12)	3499	-5594	7054	37
H(13)	3134	-5960	5314	35
H(14)	2190	-4498	4290	27
H(2N)	310(60)	2390(40)	3610(20)	41(16)
H(3N)	-220(60)	3440(30)	4170(40)	49(17)
H(5N)	280(40)	-2170(30)	3570(30)	18(12)
H(6N)	160(50)	-3290(30)	4040(40)	40(15)

Table S13. Torsion angles [°] for complex **4**.

Cu(1)-N(1)-C(1)-N(2)	-9.3(6)
Cu(1)-N(1)-C(1)-C(2)	168.8(3)
N(1)-C(1)-C(2)-C(7)	7.9(5)
N(2)-C(1)-C(2)-C(7)	-173.9(3)
N(1)-C(1)-C(2)-C(3)	-174.0(4)
N(2)-C(1)-C(2)-C(3)	4.2(5)
C(7)-C(2)-C(3)-C(4)	-0.3(5)
C(1)-C(2)-C(3)-C(4)	-178.4(3)
C(2)-C(3)-C(4)-C(5)	-0.7(6)
C(3)-C(4)-C(5)-C(6)	0.9(6)
C(4)-C(5)-C(6)-C(7)	0.0(6)
C(3)-C(2)-C(7)-C(6)	1.3(6)
C(1)-C(2)-C(7)-C(6)	179.4(3)
C(5)-C(6)-C(7)-C(2)	-1.1(6)
Cu(1)-N(3)-C(8)-N(4)	-2.7(6)
Cu(1)-N(3)-C(8)-C(9)	175.7(3)
N(3)-C(8)-C(9)-C(14)	-137.6(4)
N(4)-C(8)-C(9)-C(14)	40.9(5)
N(3)-C(8)-C(9)-C(10)	40.3(5)
N(4)-C(8)-C(9)-C(10)	-141.2(4)
C(14)-C(9)-C(10)-C(11)	-0.9(6)
C(8)-C(9)-C(10)-C(11)	-178.9(3)
C(9)-C(10)-C(11)-C(12)	1.5(6)
C(10)-C(11)-C(12)-C(13)	-0.7(6)
C(11)-C(12)-C(13)-C(14)	-0.7(6)
C(10)-C(9)-C(14)-C(13)	-0.5(6)
C(8)-C(9)-C(14)-C(13)	177.4(3)
C(12)-C(13)-C(14)-C(9)	1.3(6)
Cu(1)-O(1)-C(15)-O(3)	179.1(3)
Cu(1)-O(1)-C(15)-O(2)	-1.0(3)
Cu(1)-O(2)-C(15)-O(3)	-179.1(3)
Cu(1)-O(2)-C(15)-O(1)	1.0(3)

Symmetry transformations used to generate equivalent atoms:

Table S14. Hydrogen bonds for complex **4** [Å and °].

D-H...A	d(D-H)	d(H...A)	d(D...A)	<(DHA)
N(3)-H(4N)...O(3)#1	0.80(5)	2.30(5)	3.066(4)	161(4)
N(2)-H(2N)...O(1)	0.874(14)	2.04(3)	2.850(4)	154(5)
N(2)-H(3N)...O(3)#2	0.884(14)	2.146(18)	3.017(4)	168(5)
N(4)-H(5N)...O(2)	0.869(14)	2.26(4)	2.872(4)	128(4)
N(4)-H(6N)...O(3)#3	0.874(14)	2.045(16)	2.911(4)	171(5)

Symmetry transformations used to generate equivalent atoms:
#1 -x+1/2,-y,z+1/2 #2 -x,y+1/2,-z+1/2 #3 -x,y-1/2,-z+1/2

(*Z*)-*N'*-(4-(trifluoromethyl)phenyl)benzimidamide (**10**)

Table S15. Crystal data and structure refinement for **10**.

Identification code	bur_aug19	
Empirical formula	C14 H11 F3 N2	
Formula weight	264.25	
Temperature	123(2) K	
Wavelength	1.54184 Å	
Crystal system	Triclinic	
Space group	P -1	
Unit cell dimensions	a = 5.4166(7) Å	α= 76.595(11)°.
	b = 8.4710(11) Å	β= 84.634(11)°.
	c = 13.9435(19) Å	γ = 86.123(11)°.
Volume	618.93(14) Å^3	
Z	2	
Density (calculated)	1.418 Mg/m^3	
Absorption coefficient	1.004 mm^{-1}	
F(000)	272	
Crystal size	0.40 x 0.20 x 0.10 mm^3	
Theta range for data collection	5.623 to 69.996°.	
Index ranges	-6<=h<=4, -10<=k<=10, -16<=l<=16	
Reflections collected	3917	
Independent reflections	2344 [R(int) = 0.0344]	
Completeness to theta = 69.996°	99.4 %	
Absorption correction	Semi-empirical from equivalents	
Max. and min. transmission	1.00000 and 0.30576	

Refinement method	Full-matrix least-squares on F^2
Data / restraints / parameters	2344 / 0 / 180
Goodness-of-fit on F^2	1.044
Final R indices [I>2sigma(I)]	R1 = 0.0601, wR2 = 0.1651
R indices (all data)	R1 = 0.0750, wR2 = 0.1850
Extinction coefficient	n/a
Largest diff. peak and hole	0.381 and -0.318 e.$Å^{-3}$

Table S16. Atomic coordinates (x 10^4) and equivalent isotropic displacement parameters ($Å^2$x 10^3) for **10**. U(eq) is defined as one third of the trace of the orthogonalized U^{ij} tensor.

	x	y	z	U(eq)
F(1)	6528(5)	960(2)	557(1)	82(1)
F(2)	7395(5)	3427(3)	308(2)	88(1)
F(3)	3805(5)	2860(4)	120(2)	100(1)
N(1)	3415(3)	2582(2)	4737(2)	35(1)
N(2)	-897(4)	2437(3)	4734(2)	45(1)
C(1)	5600(6)	2390(4)	697(2)	52(1)
C(2)	4898(5)	2386(3)	1754(2)	40(1)
C(3)	3361(5)	3641(3)	1995(2)	42(1)
C(4)	2818(4)	3707(3)	2974(2)	39(1)
C(5)	3774(4)	2516(3)	3730(2)	34(1)
C(6)	5291(4)	1251(3)	3478(2)	36(1)
C(7)	5858(4)	1193(3)	2498(2)	39(1)
C(8)	1184(4)	2575(3)	5161(2)	35(1)
C(9)	865(4)	2607(3)	6228(2)	35(1)
C(10)	-1061(4)	1825(3)	6850(2)	39(1)
C(11)	-1262(5)	1781(3)	7854(2)	42(1)
C(12)	468(5)	2525(3)	8248(2)	43(1)
C(13)	2373(5)	3329(3)	7633(2)	42(1)
C(14)	2596(4)	3369(3)	6627(2)	38(1)

Table S17. Bond lengths [Å] and angles [°] for **10**.

F(1)-C(1)	1.330(3)
F(2)-C(1)	1.344(4)
F(3)-C(1)	1.302(4)
N(1)-C(8)	1.294(3)
N(1)-C(5)	1.413(3)
N(2)-C(8)	1.347(3)
N(2)-H(1N)	0.86(3)
N(2)-H(2N)	0.88(3)
C(1)-C(2)	1.488(4)
C(2)-C(7)	1.382(4)
C(2)-C(3)	1.389(4)
C(3)-C(4)	1.383(4)
C(3)-H(3)	0.9500
C(4)-C(5)	1.391(3)
C(4)-H(4)	0.9500
C(5)-C(6)	1.396(3)
C(6)-C(7)	1.384(3)
C(6)-H(6)	0.9500
C(7)-H(7)	0.9500
C(8)-C(9)	1.489(3)
C(9)-C(10)	1.392(3)
C(9)-C(14)	1.397(3)
C(10)-C(11)	1.386(4)
C(10)-H(10)	0.9500
C(11)-C(12)	1.383(4)
C(11)-H(11)	0.9500
C(12)-C(13)	1.386(4)
C(12)-H(12)	0.9500
C(13)-C(14)	1.389(4)
C(13)-H(13)	0.9500
C(14)-H(14)	0.9500
C(8)-N(1)-C(5)	119.31(19)
C(8)-N(2)-H(1N)	121.6(19)
C(8)-N(2)-H(2N)	118(2)
H(1N)-N(2)-H(2N)	118(3)

F(3)-C(1)-F(1)	108.0(3)
F(3)-C(1)-F(2)	104.2(3)
F(1)-C(1)-F(2)	104.6(3)
F(3)-C(1)-C(2)	114.2(3)
F(1)-C(1)-C(2)	113.3(2)
F(2)-C(1)-C(2)	111.7(2)
C(7)-C(2)-C(3)	119.8(2)
C(7)-C(2)-C(1)	120.7(2)
C(3)-C(2)-C(1)	119.5(2)
C(4)-C(3)-C(2)	120.1(2)
C(4)-C(3)-H(3)	120.0
C(2)-C(3)-H(3)	120.0
C(3)-C(4)-C(5)	120.8(2)
C(3)-C(4)-H(4)	119.6
C(5)-C(4)-H(4)	119.6
C(4)-C(5)-C(6)	118.5(2)
C(4)-C(5)-N(1)	123.1(2)
C(6)-C(5)-N(1)	118.3(2)
C(7)-C(6)-C(5)	120.8(2)
C(7)-C(6)-H(6)	119.6
C(5)-C(6)-H(6)	119.6
C(2)-C(7)-C(6)	120.1(2)
C(2)-C(7)-H(7)	120.0
C(6)-C(7)-H(7)	120.0
N(1)-C(8)-N(2)	125.5(2)
N(1)-C(8)-C(9)	118.2(2)
N(2)-C(8)-C(9)	116.2(2)
C(10)-C(9)-C(14)	119.1(2)
C(10)-C(9)-C(8)	121.3(2)
C(14)-C(9)-C(8)	119.6(2)
C(11)-C(10)-C(9)	120.9(2)
C(11)-C(10)-H(10)	119.6
C(9)-C(10)-H(10)	119.6
C(12)-C(11)-C(10)	119.8(2)
C(12)-C(11)-H(11)	120.1
C(10)-C(11)-H(11)	120.1
C(11)-C(12)-C(13)	119.8(2)
C(11)-C(12)-H(12)	120.1

C(13)-C(12)-H(12)	120.1
C(12)-C(13)-C(14)	120.7(2)
C(12)-C(13)-H(13)	119.6
C(14)-C(13)-H(13)	119.6
C(13)-C(14)-C(9)	119.6(2)
C(13)-C(14)-H(14)	120.2
C(9)-C(14)-H(14)	120.2

Symmetry transformations used to generate equivalent atoms:

Table S18. Anisotropic displacement parameters ($Å^2$x 10^3) for **10**. The anisotropic displacement factor exponent takes the form: $-2\pi^2[h^2a^{*2}U^{11} + ... + 2 h k a^* b^* U^{12}]$

	U^{11}	U^{22}	U^{33}	U^{23}	U^{13}	U^{12}
F(1)	148(2)	49(1)	49(1)	-20(1)	5(1)	20(1)
F(2)	124(2)	81(2)	58(1)	-21(1)	32(1)	-30(1)
F(3)	105(2)	152(3)	52(1)	-43(1)	-29(1)	42(2)
N(1)	39(1)	30(1)	38(1)	-10(1)	-4(1)	-2(1)
N(2)	36(1)	61(1)	40(1)	-16(1)	-2(1)	-6(1)
C(1)	71(2)	41(1)	44(2)	-12(1)	-6(1)	8(1)
C(2)	47(1)	33(1)	42(1)	-13(1)	-4(1)	-3(1)
C(3)	54(1)	31(1)	38(1)	-4(1)	-6(1)	2(1)
C(4)	44(1)	29(1)	44(1)	-11(1)	-2(1)	3(1)
C(5)	35(1)	30(1)	40(1)	-11(1)	-5(1)	-6(1)
C(6)	40(1)	28(1)	41(1)	-7(1)	-6(1)	-3(1)
C(7)	44(1)	31(1)	45(1)	-13(1)	-3(1)	0(1)
C(8)	38(1)	25(1)	42(1)	-8(1)	-5(1)	-1(1)
C(9)	36(1)	27(1)	42(1)	-11(1)	-4(1)	4(1)
C(10)	41(1)	33(1)	44(1)	-11(1)	-6(1)	-1(1)
C(11)	42(1)	36(1)	46(1)	-7(1)	2(1)	-1(1)
C(12)	50(1)	42(1)	39(1)	-14(1)	-5(1)	9(1)
C(13)	44(1)	39(1)	46(1)	-17(1)	-10(1)	4(1)
C(14)	38(1)	33(1)	43(1)	-11(1)	-3(1)	0(1)

Table S19. Hydrogen coordinates (x 10^4) and isotropic displacement parameters ($Å^2$x 10^3) for **10**.

	x	y	z	U(eq)
H(3)	2681	4455	1485	50
H(4)	1781	4576	3132	47
H(6)	5941	419	3986	43
H(7)	6909	332	2336	47
H(10)	-2254	1313	6582	46
H(11)	-2584	1241	8270	50
H(12)	351	2485	8937	52
H(13)	3539	3858	7903	50
H(14)	3919	3912	6213	45
H(1N)	-2340(50)	2490(30)	5040(20)	38(7)
H(2N)	-800(50)	2530(40)	4090(30)	47(8)

Table S20. Torsion angles [°] for **10**.

F(3)-C(1)-C(2)-C(7)	143.4(3)
F(1)-C(1)-C(2)-C(7)	19.2(4)
F(2)-C(1)-C(2)-C(7)	-98.6(3)
F(3)-C(1)-C(2)-C(3)	-39.9(4)
F(1)-C(1)-C(2)-C(3)	-164.0(3)
F(2)-C(1)-C(2)-C(3)	78.1(3)
C(7)-C(2)-C(3)-C(4)	0.8(4)
C(1)-C(2)-C(3)-C(4)	-176.0(2)
C(2)-C(3)-C(4)-C(5)	-0.8(4)
C(3)-C(4)-C(5)-C(6)	-0.1(3)
C(3)-C(4)-C(5)-N(1)	175.6(2)
C(8)-N(1)-C(5)-C(4)	61.6(3)
C(8)-N(1)-C(5)-C(6)	-122.7(2)
C(4)-C(5)-C(6)-C(7)	0.8(3)
N(1)-C(5)-C(6)-C(7)	-175.11(19)
C(3)-C(2)-C(7)-C(6)	-0.1(4)
C(1)-C(2)-C(7)-C(6)	176.7(2)
C(5)-C(6)-C(7)-C(2)	-0.7(3)
C(5)-N(1)-C(8)-N(2)	3.1(3)
C(5)-N(1)-C(8)-C(9)	178.60(19)
N(1)-C(8)-C(9)-C(10)	-147.3(2)
N(2)-C(8)-C(9)-C(10)	28.6(3)
N(1)-C(8)-C(9)-C(14)	29.6(3)
N(2)-C(8)-C(9)-C(14)	-154.5(2)
C(14)-C(9)-C(10)-C(11)	-0.6(4)
C(8)-C(9)-C(10)-C(11)	176.3(2)
C(9)-C(10)-C(11)-C(12)	0.1(4)
C(10)-C(11)-C(12)-C(13)	0.9(4)
C(11)-C(12)-C(13)-C(14)	-1.3(4)
C(12)-C(13)-C(14)-C(9)	0.7(4)
C(10)-C(9)-C(14)-C(13)	0.2(3)
C(8)-C(9)-C(14)-C(13)	-176.7(2)

Symmetry transformations used to generate equivalent atoms:

Table S21. Hydrogen bonds for **10** [Å and °].

D-H...A	d(D-H)	d(H...A)	d(D...A)	<(DHA)
N(2)-H(1N)...N(1)#1	0.86(3)	2.37(3)	3.074(3)	140(2)

Symmetry transformations used to generate equivalent atoms:

#1 x-1,y,z

References

(1) Scala, A.; Piperno, A.; Micale, N.; Mineo, P. G.; Abbadessa, A.; Risoluti, R.; Castelli, G.; Bruno, F.; Vitale, F.; Cascio, A.; Grassi, G. "Click" on PLGA-PEG and Hyaluronic Acid: Gaining Access to Anti-Leishmanial Pentamidine Bioconjugates. *J. Biomed. Mater. Res. - Part B Appl. Biomater.* **2018**, *106*.

(2) Nvau, J. B.; Alenezi, S.; Ungogo, M. A.; Alfayez, I. A. M.; Natto, M. J.; Gray, A. I.; Ferro, V. A.; Watson, D. G.; de Koning, H. P.; Igoli, J. O. Antiparasitic and Cytotoxic Activity of Bokkosin, A Novel Diterpene-Substituted Chromanyl Benzoquinone From Calliandra Portoricensis. *Front. Chem.* **2020**.

(3) Carruthers, L. V.; Munday, J. C.; Ebiloma, G. U.; Steketee, P.; Jayaraman, S.; Campagnaro, G. D.; Ungogo, M. A.; Donachie, A. M.; Rowan, T. G.; Peter, R.; Morrison, L. J.; Barrett, M. P.; de Koning, H. P. Diminazene Resistance in Trypanosoma Congolense Is Linked to Reduced Mitochondrial Membrane Potential and Not to Reduced Transport Capacity. *bioRxiv*. **2020**.

(4) Coustou, V.; Guegan, F.; Plazolles, N.; Baltz, T. Complete In Vitro Life Cycle of Trypanosoma Congolense: Development of Genetic Tools. *PLoS Negl. Trop. Dis.* **2010**, *4*, e618.

(5) Al-Salabi, M. I.; Wallace, L. J. M.; De Koning, H. P. A Leishmania Major Nucleobase Transporter Responsible for Allopurinol Uptake Is a Functional Homolog of the Trypanosoma Brucei H2 Transporter. *Mol. Pharmacol.* **2003**, *63* (4), 814–820.

(6) Cueto-Díaz, E. J.; Ebiloma, G. U.; Alfayez, I. A.; Ungogo, M. A.; Lemgruber, L.; González-García, M. C.; Giron, M. D.; Salto, R.; Fueyo-González, F. J.; Shiba, T.; González-Vera, J. A.; Ruedas Rama, M. J.; Orte, A.; de Koning, H. P.; Dardonville, C. Synthesis, Biological, and Photophysical Studies of Molecular Rotor-Based Fluorescent Inhibitors of the Trypanosome Alternative Oxidase. *Eur. J. Med. Chem.* **2021**, *220*, 113470.
